# The relation between specific motor skills and daily living skills in autistic children and adolescents

**DOI:** 10.3389/fnint.2024.1334241

**Published:** 2024-05-22

**Authors:** Emily C. Skaletski, Sailery Cortes Cardona, Brittany G. Travers

**Affiliations:** ^1^Occupational Therapy Program, Department of Kinesiology, University of Wisconsin-Madison, Madison, WI, United States; ^2^Waisman Center, University of Wisconsin-Madison, Madison, WI, United States

**Keywords:** motor skills, autism, daily living skills, cognition, age

## Abstract

**Introduction:**

Motor skill difficulties are common in autistic children and are related to daily living skills (DLS). However, it remains unclear which specific motor tasks are most likely to impact overall DLS. This study sought to fill this gap.

**Methods and results:**

In 90 autistic children and adolescents (ages 6–17 years), we found that fine/manual motor tasks, like drawing or folding, demonstrated significant medium-sized relations with DLS, even after accounting for IQ and sensory features, whereas tasks in the areas of bilateral coordination, upper-limb coordination, and balance only related to DLS (small effect sizes) prior to accounting for IQ and sensory features. When looking at an overall balance score, we found that IQ significantly interacted on the relation between overall balance and DLS.

**Discussion:**

These results further demonstrate the particular importance of fine/manual motor skills for DLS in autistic youth, even when accounting for IQ and sensory features. Indeed, accounting for sensory features strengthened the relations between fine/manual motor skills and DLS. Our findings provide evidence of the impact of cognitive factors on the relation between balance and DLS, indicating that it may be that autistic individuals with lower IQs experience relations between balance and DLS that are different than their peers with higher IQs. Our findings support the benefit of considering individual motor skills rather than domain-level information when assessing ways to promote DLS in autistic youth. The results further shed light on the importance of fine motor skills, as well as the unique relationship of balance and DLS in autistic individuals with lower IQs.

## Introduction

Numerous studies converge to suggest that motor and daily living skills are associated in autistic and non-autistic individuals (Travers et al., 2017, 2022; Bremer and Cairney, 2018; Fisher et al., 2018; Fears et al., 2022), such that higher motor performance maps onto higher daily living skill (DLS) performance. Moreover, motor skills may help explain why DLS performance is often discrepant from IQ in autistic individuals (Fears et al., 2022). As such, motor skills and DLS appear to be importantly interlinked, but we are only beginning to understand which specific motor skills (i.e., bilateral coordination, strength, balance, fine motor precision/integration) are most likely to impact overall DLS in autistic children and adolescents. Gaining this information is of critical importance because it may help guide collaborative approaches that target motor skills in autistic individuals with the goal of eliciting the DLS changes that the individual desires. Therefore, the present study set out to fill this key gap.

Motor skills are a common area of difficulty for autistic individuals (Licari et al., 2020; Miller et al., 2024), impacting both general (Purpura et al., 2020; Mohd Nordin et al., 2021; Kilroy et al., 2022) and specific areas of motor functioning, including coordination (Fournier et al., 2010; Bhat et al., 2011), postural stability (Bhat et al., 2011; Fisher et al., 2018), and imitation and praxis (Bhat et al., 2011; Kilroy et al., 2022). Motor skills are also related to sensory features (Surgent et al., 2021), IQ (Fulceri et al., 2019; Surgent et al., 2021; Ramos-Sánchez et al., 2022), and DLS (Jasmin et al., 2009; MacDonald et al., 2013; Mattard-Labrecque et al., 2013; MacDonald et al., 2017; Travers et al., 2017; Bremer and Cairney, 2018; Fears et al., 2022; Travers et al., 2022). Sensory features (Jasmin et al., 2009; Mattard-Labrecque et al., 2013; Williams et al., 2018; Travers et al., 2022) and IQ (Kenworthy et al., 2010; Pugliese et al., 2015) have also been found to relate to DLS. Taken together, these findings suggest that motor difficulties are prevalent in autistic individuals and that specific motor difficulties, in combination with other factors like sensory features and IQ, may impact DLS to differing degrees.

Because DLS encompasses a wide variety of tasks (e.g., from toileting to banking), a recent study from our group investigated which specific DLS tasks were best predicted by a composite, summary measure of motor performance in autistic and non-autistic children (Travers et al., 2022). We found that personal, domestic, and community domains of DLS were all associated with individual differences in general motor performance, and we found that specific household tasks (clean up and dressing), educational tasks (calendaring), and safety tasks (thermometer use and crossing the street) were specifically predicted by motor performance. While this study showed which specific DLS tasks may be most associated with general motor features, the reverse (i.e., which specific motor tasks predict general DLS) remains unclear. Indeed, the majority of studies have used overall motor scores (Travers et al., 2022) or gross and fine motor skill scores (MacDonald et al., 2013; Günal et al., 2019) to understand the relation between motor skills and DLS in autistic individuals. However, a handful of studies have explored more specific areas of motor performance. For example, finger tapping speed and grip strength were found to predict concurrent and future DLS in a large, longitudinal sample of autistic and non-autistic individuals ages 5–40 years (Travers et al., 2017). In addition, two studies (Bremer and Cairney, 2018; Fears et al., 2022) used Movement-ABC (M-ABC) subscales to examine how manual dexterity (i.e., moving or placing coins or pegs, threading items, or drawing a path), aiming and catching (i.e., catching or throwing objects), and balance (i.e., balancing on one leg or on a board, walking in different ways, or hopping) related to DLS and adaptive behavior in autistic children and adolescents. Both studies converged to find that manual dexterity was the only motor subdomain of the M-ABC that significantly correlated with DLS or adaptive behavior. Together, these findings suggest that motor tasks that engage the fingers and hands may be particularly related to DLS in autistic individuals. However, while the M-ABC is commonly used, it is not all-encompassing, and therefore we do not know how other motor domains, such as bilateral coordination, running speed and agility, and strength, relate to DLS. Further, as motor skill assessments like the M-ABC or Bruininks-Oseretsky Test of Motor Proficiency, 2nd edition (BOT-2) (Bruininks and Bruininks, 2005) differ in how tasks are divided into categories, exploring task-level relations will provide insight across measures, increasing clinical utility.

Many daily activities, such as getting dressed or household cleaning tasks, have inherent balance demands, such as the postural stability required to put on pants or the weight-shifting required to move laundry from a washing machine to a dryer. Intriguingly, past studies involving autistic children and adolescents have not found balance to be a predictor of DLS (Bremer and Cairney, 2018; Fisher et al., 2018; Fears et al., 2022). However, if balance truly does not relate to DLS, addressing balance with the end goal of improving DLS may be an ineffective intervention target. One possibility is that there might be additional factors at play that moderate the relation between balance and DLS, such as IQ. A prior study from our group (Fisher et al., 2018) found a significant interaction effect between IQ and balance, such that only autistic children and adolescents with lower IQs showed a relation between balance and DLS. Autistic children and adolescents with higher IQs did not show this relation, even though the ranges of balance scores were similar. However, to our knowledge, this is the only study to have examined IQ as a moderator of the relation between balance and DLS in autistic individuals, and further research is needed to clarify if there truly is no relation between balance and DLS across autistic individuals, or if there are additional factors, such as IQ, at play.

Based on the reviewed literature, the first aim of this study was to determine how specific motor skills on the BOT-2 Short Form relate to a summary measure of DLS in autistic children. These BOT-2 Short Form items include tasks in the areas of fine motor precision, fine motor integration, manual dexterity, upper-limb coordination, bilateral coordination, balance, running speed and agility, and strength. To our knowledge, this is the first study in autistic individuals to examine the domains of bilateral coordination, running speed and agility, and strength in relation to DLS. Given the associations between sensory features and IQ on both motor skills and DLS in the aforementioned literature, we then completed a follow-up analysis of the relation between the BOT-2 Short Form items and DLS, first covarying for IQ and then covarying for sensory features. The second aim of this study was to expand upon prior findings (Fisher et al., 2018) to understand if IQ moderates the relation between balance and DLS in autistic children and adolescents.

## Materials and methods

### Design and participants

This retrospective cross-sectional study included 90 autistic children and adolescents ages 6.22 to 17.85 years old who initially participated in one of three studies. See Table 1 for additional participant demographics. Participants were recruited through Waisman Center registries of eligible participants, emails to the university community, as well as flyers posted at community organizations and relevant clinics. Participants had prior autism diagnoses and met criteria on the Autism Diagnostic Observation Schedule-2nd edition (ADOS-2), with supplementary gathered through the Social Responsiveness Scale-2nd edition (SRS-2) and Social Communication Questionnaire (SCQ). The SCQ was unavailable for one participant due to age of adoption, but the participant’s ADOS-2 and SRS-2 scores exceeded the cutoff for an autism diagnosis. Participants needed to be English-speaking and could not have a prior diagnosis of tuberous sclerosis, Down syndrome, fragile X syndrome, or hypoxia-ischemia; history of a severe head injury; significant uncorrected hearing or vision loss; inability to provide assent; or contraindication to magnetic resonance imaging. This study was performed in line with the principles of the 1964 Declaration of Helsinki and later amendments. Approval was granted by the Institutional Review Board (#2014–1,248, #2014–1,499 #2016–0441, #2022–0413). Parents or legal guardians provided informed consent, and participants provided either verbal or written assent.

**Table 1:** Demographic characteristics.

	Mean(SD)	Range
Sex (% female)	12.22%	
Age (in years)	11.58(3.46)	6.22–17.85
WASI-2 IQ	102.29 (16.72)	62–136
ADOS-2 Module 3 overall total	12.64 (5.17)	3–27
ADOS-2 Module 4 overall total	13.43 (5.48)	1–28
SRS-2 T-score	75.17 (10.42)	48–90
SCQ Total raw	18.96 (7.27)	6–37
BOT-2 SF Standard score	38.45 (6.94)	24–68
BOT-2 Balance T-score	10.20 (4.66)	2–24
VABS-II DLS Standard score	88.64 (16.11)	54–121
Hispanic	6.67%	
Race		
White	81.11%	
More than one	5.56%	
Asian	5.56%	
Black/African American	6.67%	
American Indian/Alaskan Native	1.11%	
Grade		
Kindergarten	3.33%	
1st	8.89%	
2nd	8.89%	
3rd	8.89%	
4th	17.78%	
5th	10.00%	
6th	0.00%	
7th	6.67%	
8th	6.67%	
9th	6.67%	
10th	14.44%	
11th	5.56%	
12th	2.22%	

IQ = intelligence quotient; WASI-2 = Wechsler Abbreviated Scales of Intelligence, 2nd edition; ADOS-2 = Autism Diagnostic Observation Schedule-2nd edition; SRS-2 = Social Responsiveness Scale-2nd edition; SCQ = Social Communication Questionnaire; BOT-2 = Bruininks-Oseretsky Test of Motor Proficiency, 2nd edition; VABS-II = Vineland Adaptive Behavior Scales, 2_nd_ edition.

### Measures

Bruininks-Oseretsky Test of Motor Proficiency, 2nd edition (BOT-2) Short Form and balance subscale T-score (Bruininks and Bruininks, 2005). Motor skills were assessed using items from the BOT-2 Short Form (BOT-2 SF) and balance subscale. The BOT-2 SF is a reliable and valid abbreviated screening measure that demonstrates reliability and validity (Bruininks and Bruininks, 2005). Two items, copying a square and copying a star, were combined to ensure adequate responses for individual point value options and due to the similar nature of these items as components of the fine motor integration section. When two trials were administered for an item, the highest score between the two trials was used for analysis. For the current analyses, we used item-level BOT-2 SF data for Aim 1, along with the balance subscale T-scores for Aim 2. One participant did not complete the balance subscale due to time constraints and is therefore only included in analyses for Aim 1. A separate participant only completed the balance subscale and is therefore only included in analyses for Aim 2.

Vineland Adaptive Behavior Scales, 2nd edition (VABS-II) (Sparrow et al., 2005). DLS were assessed using the VABS-II. The VABS-II is a reliable and valid caregiver report measure that evaluates social, communication, and DLS across development, along with motor skills in young children. For the current analyses, we used the DLS standard scores.

Wechsler Abbreviated Scales of Intelligence, 2nd edition (WASI-2) (Wechsler, 2011). IQ was measured using the WASI-2, which consists of subtests in the areas of block design, vocabulary, matrix reasoning, and similarities. The WASI-2 demonstrates good reliability and validity (Wechsler, 2011). Either the full-scale IQ from two tests (FSIQ-2) or full-scale IQ from four tests (FSIQ-4) was used. To obtain a score for the FSIQ-4, all four subtests are used, while the vocabulary and matrix reasoning subtests are used to calculate the FSIQ-2. For cases in which the participant completed all four subtests, we used the higher of the two IQ scores (FSIQ-2 or FSIQ-4). We use IQ as a continuous variable as it is highly recommended to use continuous variables in their continuous form (DeCoster et al., 2009).

Sensory Experiences Questionnaire, Version 3.0 (SEQ) (Baranek, 2009). Sensory features were measured using the SEQ, a caregiver report measure with strong test–retest reliability and internal consistency (Little et al., 2011). The SEQ includes questions related to sensory seeking, hyporesponsiveness, hyperresponsiveness, and enhanced perception across multiple areas of sensory processing.

### Data analysis

Analyses were performed in R version 4.1.0 (R Core Team, 2023). Alpha was set at 0.05 for all analyses. Prior to performing analyses, we performed data visualization to check the assumptions of our statistical approach. We observed that two of the BOT-2 SF items, tapping feet and fingers-same sides and walking forward on a line, had more than 80% of observations at the ceiling, and we therefore excluded them from further analyses. Next, to make sure that all BOT-2 items were on the same scale, we converted BOT-2 item raw scores to z-scores. Because the BOT-2 item scores are not age-normed and may have non-linear relationships with age, we used a sample of 352 autistic and non-autistic participants available to us (90 of whom are included in this study), and we examined age trajectories on these BOT-2 SF items. We found that the age effects were best represented by a log fit line, which was then used to create a z-scored age-normed variable for each of the BOT-2 SF items. To assess Aim 1, Spearman correlations (due to non-linear relations in the scatterplots) were performed examining each of the z-scored, age-normed BOT-2 SF items in relation to DLS standard scores. False discovery rate (FDR) (Benjamini and Hochberg, 1995) was used to correct for the 11 comparisons. In light of associations between sensory features and IQ with both motor skills (Fulceri et al., 2019; Surgent et al., 2021; Ramos-Sánchez et al., 2022) and DLS (Jasmin et al., 2009; Kenworthy et al., 2010; Mattard-Labrecque et al., 2013; Pugliese et al., 2015; Williams et al., 2018; Travers et al., 2022), follow-up analyses examined whether the relations between BOT-2 items and DLS remained when accounting for IQ and sensory features. As three participants did not have SEQ data due to time of participation, the follow-up analyses included 86 participants.

To assess Aim 2’s hypothesized interaction between IQ and BOT-2 balance subscale T-scores on DLS, we used multiple regression. As a portion of the autistic participants in this current study were included as a part of that prior study (Fisher et al., 2018), we ran regressions both with (*n* = 89) and without (*n* = 35) these participants. We performed the following regression: DLS Standard Score ~ Balance T-score + IQ + Balance T-score*IQ.

## Results

**Figure 1:**
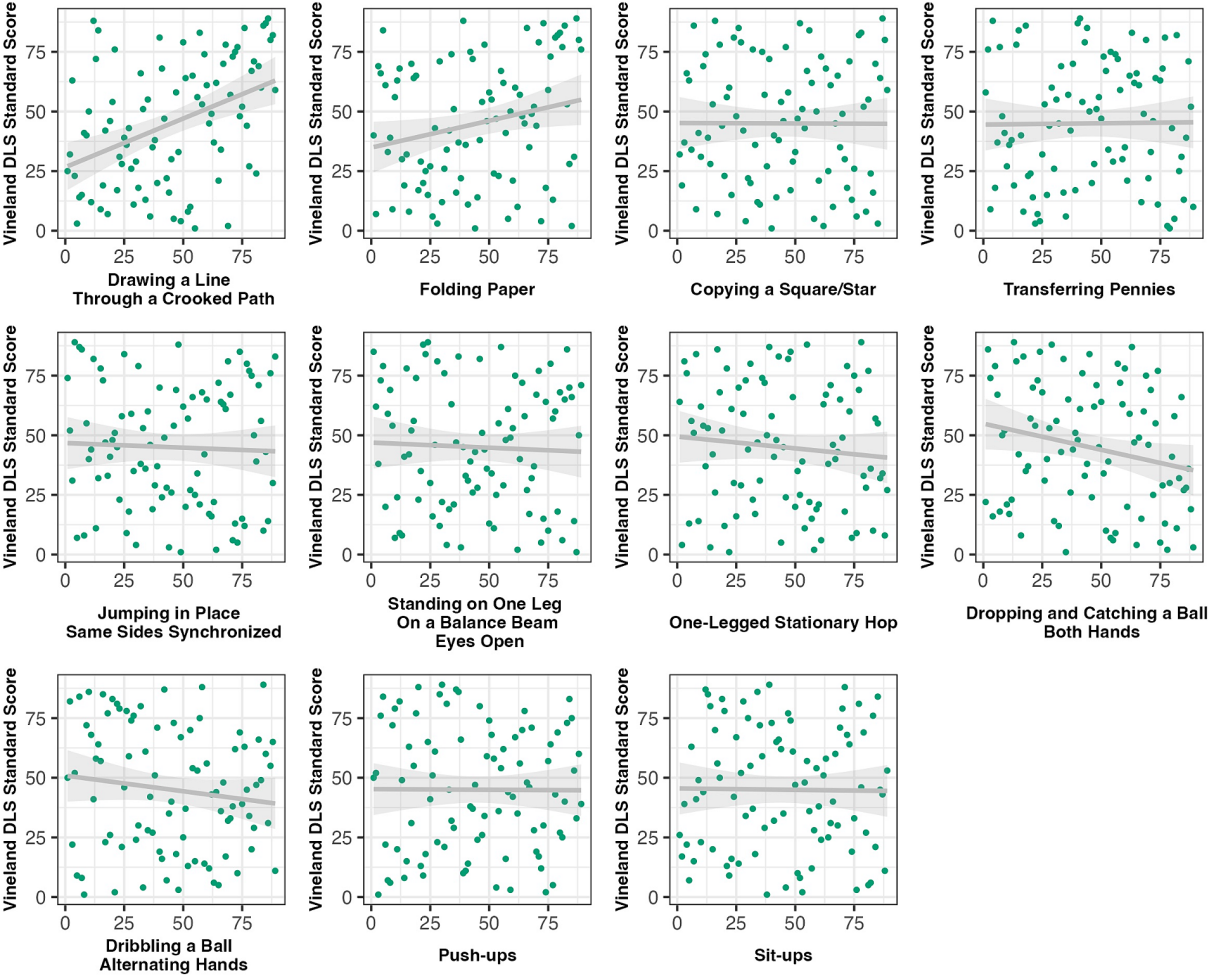
Scatterplots of the relations between items on the Bruininks-Oseretsky Test of Motor Proficiency, 2nd edition (BOT-2) Short Form (accounting for age) and the Vineland Adaptive Behavior Scales, 2nd (VABS-II) edition Daily Living Skills (DLS) domain standard scores, in z-scores (standard deviation units) and Spearman ranked.

As can be seen in Table 2 and Figure 1, DLS demonstrated significant medium-sized effects with items in the areas of fine motor precision (i.e., drawing a line through a crooked path and folding paper) and bilateral coordination (i.e., jumping in place-same sides synchronized). Tasks in the areas of fine motor integration (i.e., copying a square/star), manual dexterity (i.e., transferring pennies), upper-limb coordination (i.e., dropping and catching a ball with both hands, dribbling a ball-alternating hands), and balance (standing on one leg on a balance beam with eyes open) demonstrated significant small-sized effects. Correlations between DLS and tasks in the areas of running speed and agility (i.e., one-legged stationary hop) and strength (i.e., push-ups, sit-ups) were non-significant (*r*’s ≤ 0.10). Follow-up analyses accounting for IQ and sensory features can be found in Supplementary Table 1. Accounting for IQ, only fine motor precision tasks significantly related to DLS with small-sized effects. Accounting for sensory features, only fine motor precision, fine motor integration, and manual dexterity tasks significantly related to DLS with medium-sized effects.

**Table 2:** Results of the Spearman correlations between Bruininks-Oseretsky Test of Motor Proficiency, 2nd edition (BOT-2) Short Form item z-scores, accounting for age, and Vineland Adaptive Behavior Scales, 2nd edition Daily Living Skills standard scores (DLS).

	*r*	*p*	*p.adj*
Fine motor precision			
Drawing a line through a crooked path	0.40	<0.001	<0.001
Folding paper	0.39	<0.001	<0.001
Fine motor integration			
Copying a square/star	0.26	0.01	0.02
Manual dexterity			
Transferring pennies	0.28	0.01	0.02
Upper-limb coordination			
Dropping and catching a ball-both hands	0.23	0.03	0.04
Dribbling a ball-alternating hands	0.24	0.02	0.03
Bilateral coordination			
Jumping in place-same sides synchronized	0.31	0.003	0.01
Balance			
Standing on one leg on a balance beam-eyes open	0.27	0.01	0.02
Running speed & agility			
One-legged stationary hop	0.10	0.36	0.43
Strength			
Push-ups	0.02	0.83	0.85
Sit-ups	0.07	0.52	0.57

**Figure 2:**
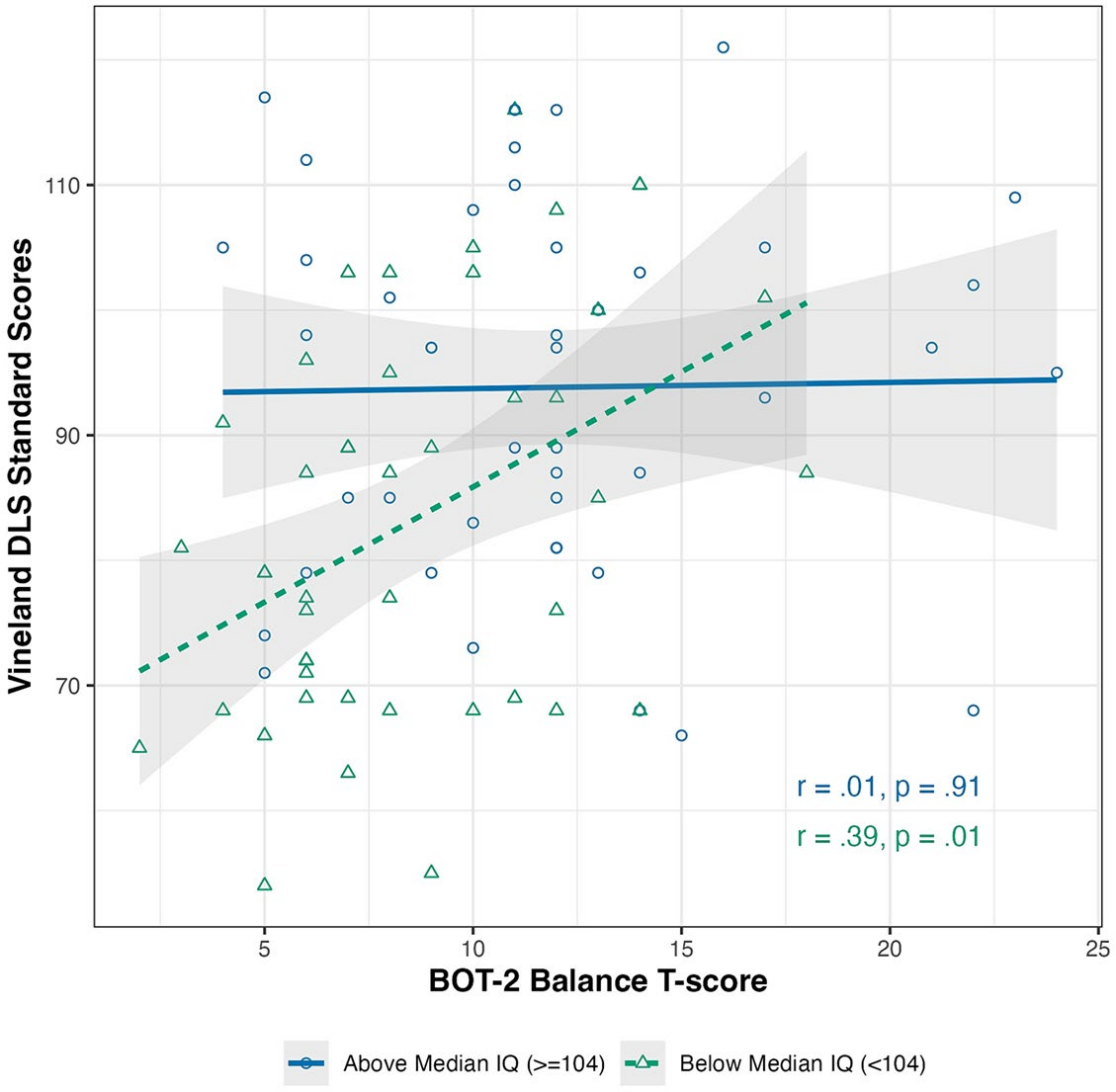
Scatterplot of the relation between Bruininks-Oseretsky Test of Motor Proficiency, 2nd edition (BOT-2) Balance subscale T-scores and the Vineland Adaptive Behavior Scales, 2nd (VABS-II) edition Daily Living Skills domain standard score. Graphs were created using a median split to visualize the results in light of IQ.

There was a significant small-sized interaction of IQ on the relation between balance and DLS in the full sample of autistic participants (which included participants from Fisher et al., 2018). When looking solely at participants unique to the current study (*n* = 35), a similar effect size was found (Cohen’s *d* = 0.46 and *d* = 0.53, respectively) (see Table 3 and Figure 2), but the effect in the smaller group was not significant.

**Table 3:** Results of regression looking at the interaction effect of IQ on the relation between BOT-2 Balance subscale T-scores and DLS.

	*b*	*SE*	*t*	*p*	*d*
Full sample	*b*	*SE*	*t(85)*	*p*	*d*
IQ interaction	−0.05	0.02	−2.11	0.04	0.46
Unique participants	*b*	*SE*	*t(31)*	*p*	*d*
Balance T-score	5.71	2.94	1.94	0.06	0.70
IQ	0.56	0.32	1.76	0.09	0.63
IQ interaction	−0.04	0.03	−1.47	0.15	0.53

Full sample indicates all eligible participants, whereas unique participants indicates those who were not in the prior analyses performed by Fisher et al. (2018). IQ = intelligence quotient; BOT-2 = Bruininks-Oseretsky Test of Motor Proficiency, 2nd edition; DLS = Vineland Adaptive Behavior Scales, 2nd edition Daily Living Skills standard scores.

## Discussion

In this study, we explored how different motor skill tasks related to DLS in autistic children. First, we looked at how individual items on the BOT-2 SF related to DLS. Similar to past studies (Bremer and Cairney, 2018; Fears et al., 2022), we found medium-sized effects of tasks in the areas of fine motor precision and bilateral coordination and small-sized effects of tasks in the areas of fine motor integration, manual dexterity, upper-limb coordination, and balance. After accounting for IQ, only tasks in the area of fine motor precision remained significant with a small effect size. When accounting for sensory features, fine motor precision remained significant with a medium effect size, but fine motor integration and manual dexterity increased in effect size. Taken together, these findings affirm the particular importance of fine/manual motor skills for DLS. Previous work suggests that micromovements (i.e., variations in movement during tasks) may impact fine/manual motor skills, such as pointing (Torres et al., 2013). These micromovements may in turn lead to difficulty executing the fine motor movements required to complete DLS, such as manipulating buttons or zippers on clothing, opening containers to prepare or consume a meal, or unlocking a door with a key, all of which are important and frequent tasks across the lifespan. Future research should explore whether the relation between these motor skill areas and DLS is maintained into adulthood, as well as whether these skill areas relate to DLS focused on personal care tasks, such as dressing or bathing, rather than a composite measure of DLS.

Next, we looked at how balance relates to DLS. Similar to past studies that did not find a relationship between balance and DLS (Bremer and Cairney, 2018; Fears et al., 2022), our study only found small-sized relations between balance and DLS that were not significant after accounting for IQ or sensory features. However, when IQ was considered as a moderator (as was done in Fisher et al., 2018), we found that autistic children and adolescents with lower IQs appeared to be driving the relationship between balance and DLS. This information tells us that balance and DLS only appear to relate in autistic children and adolescents with lower IQs. This finding also poses the question of why balance and DLS do not relate in autistic children and adolescents with higher IQs. It may be that autistic children and adolescents with higher and lower IQs demonstrate distinct micromovement profiles in their balance, an area for future study. In addition, it could be that autistic children and adolescents with higher IQs use compensatory strategies for their balance difficulties, diminishing the impact that these balance difficulties have on DLS. Future research should explore what, if any, compensatory strategies are being used; whether IQ moderates balance and DLS relations across the lifespan in autistic individuals; and whether balance might relate to more specific DLS, such as dressing or cooking, which require more postural stability, endurance, and weight-shifting, whereas the current study focused on DLS as a whole.

Overall, these findings suggest that clinicians should pay particular attention to fine motor skills and the functional implications of difficulties with fine motor skills. Whether writing, dressing, or removing money from a wallet, fine motor skills are critical across the lifespan and across environments. However, this work also demonstrates the need to consider other factors, like IQ and sensory features, when considering how to best support autistic individuals. Autistic children and adolescents with lower IQs may require additional support in improving balance with the ultimate goal of improving DLS, whereas autistic children and adolescents with higher IQs may require focus on other areas to improve DLS. As accounting for sensory features maintained or strengthened the relations between fine/manual motor skills and DLS, sensory features must also be considered in order to effectively improve both fine motor skills and DLS in autistic individuals. Our results also highlight the value of using specific item scores, rather than an overall domain score, to clearly define the area of difficulty in motor skills for autistic individuals, particularly when considering how to improve DLS. However, future studies would benefit from the use of a directly observed measure of sensory features that would provide information that is unique or complementary to caregiver report.

The findings of this study should be interpreted in light of the study’s strengths and limitations. The generalizability of this study is impacted by the exclusion of autistic individuals with co-occurring conditions like Down syndrome or fragile X, the requirement of participants to use spoken language, and the majority of the sample being White and male. We also did not collect income or parent education level for the participants in this study, a limitation as income has been found to relate to DLS (Kilincaslan et al., 2019). However, our results are strengthened by our relatively large sample with a wide range of ages and IQs. Future studies would benefit from a larger and more diverse sample, in consideration of sex, race, ethnicity, co-occurring conditions, and use of spoken language. In addition, an even larger sample would allow for the inclusion of more covariates related to motor skills, such as autism features (Travers et al., 2013; MacDonald et al., 2014; Bhat, 2021; Cheung et al., 2021; Fears et al., 2022) and executive function (Pugliese et al., 2015). Lastly, future research should evaluate the relation between DLS and motor skills as a function of experience with fine motor tasks such as gaming on touchscreen devices, which was not measured in the current study.

In all, promotion of DLS is critical given the importance of DLS to quality of life (Bishop-Fitzpatrick et al., 2016; Hong et al., 2016) and intervention priorities of autistic adults (Benevides et al., 2020). This study confirms the role that motor skills play in DLS in autistic children and adolescents. The findings also demonstrate that balance and DLS relations are unique to autistic children and adolescents with IQs below the median. Overall, our findings demonstrate the need to consider motor, sensory, and cognitive factors in the context of DLS in autistic children and adolescents, emphasizing the need for individualized interventions for motor skills and DLS.

## Data availability statement

The raw data supporting the conclusions of this article will be made available by the authors, without undue reservation.

## Ethics statement

The studies involving humans were approved by Institutional Review Board of the University of Wisconsin-Madison. The studies were conducted in accordance with the local legislation and institutional requirements. Written informed consent for participation in this study was provided by the participants’ legal guardians/next of kin.

## Author contributions

ES: Conceptualization, Investigation, Writing – review & editing, Data curation, Formal analysis, Project administration, Visualization, Writing – original draft. SC: Formal analysis, Writing – original draft, Conceptualization. BT: Methodology, Conceptualization, Data curation, Funding acquisition, Investigation, Resources, Supervision, Writing – review & editing.
