# Supplementary material for: The relation between specific motor skills and daily living skills in autistic children and adolescents
Source: Front Integr Neurosci. 2024 May 22;18:1334241. doi: 10.3389/fnint.2024.1334241 (PMC11150622; doi:10.3389/fnint.2024.1334241)
Supplement: Supplementary file 1 [file Table_1.DOCX]

***Supplementary Material***

The Relation Between Specific Motor Skills and Daily Living Skills in Autistic Children and Adolescents

Emily C. Skaletski^1,2^, Sailery Cortes Cardona^1,2^, Brittany G. Travers^1,2*^

***Correspondence:** Brittany G. Travers, PhD: [btravers@wisc.edu](mailto:btravers@wisc.edu)

**Supplemental Table 1.** Results of relations between Bruininks-Oseretsky Test of Motor Proficiency, 2^nd^ edition (BOT-2) Short Form item scores, standardized for age, and Vineland Adaptive Behavior Scales, 2^nd^ edition Daily Living Skills standard scores (DLS), accounting separately for IQ (WASI-2) (Model 1) and sensory features (SEQ) (Model 2).

| **Item** | **Main Effects** | ***b*** | **SE** | ***t*** | ***d*** | ***p*** |
| --- | --- | --- | --- | --- | --- | --- |
| **Drawing a line through a crooked path** | | | | | | |
| Model 1 | **Drawing a line** | **4.14** | **1.92** | **2.16** | **0.47** | **.03** |
|  | **IQ** | **0.30** | **0.11** | **2.90** | **0.64** | **.01** |
| Model 2 | **Drawing a line** | **4.93** | **1.89** | **2.61** | **0.57** | **.01** |
|  | **SEQ** | **-0.08** | **0.03** | **-2.35** | **0.52** | **.02** |
| **Folding Paper** | | | | | | |
| Model 1 | **Folding paper** | **3.76** | **1.81** | **2.08** | **0.46** | **.04** |
|  | **IQ** | **0.31** | **0.10** | **3.00** | **0.66** | **.004** |
| Model 2 | **Folding paper** | **4.65** | **1.77** | **2.63** | **0.58** | **.01** |
|  | **SEQ** | **-0.09** | **0.03** | **-2.57** | **0.56** | **.01** |
| **Copying a square/star** | | | | | | |
| Model 1 | Copying shapes | 2.64 | 1.81 | 1.46 | 0.32 | .15 |
|  | **IQ** | **0.32** | **0.11** | **2.88** | **0.63** | **.01** |
| Model 2 | **Copying shapes** | **4.19** | **1.68** | **2.50** | **0.55** | **.01** |
|  | **SEQ** | **-0.09** | **0.03** | **-2.75** | **0.60** | **.01** |
| **Transferring pennies** | | | | | | |
| Model 1 | Transferring pennies | 2.56 | 1.92 | 1.33 | 0.29 | .19 |
|  | **IQ** | **0.32** | **0.11** | **2.92** | **0.64** | **.01** |
| Model 2 | **Transferring pennies** | **4.21** | **1.78** | **2.36** | **0.52** | **.02** |
|  | **SEQ** | **-0.09** | **0.03** | **-2.73** | **0.60** | **.01** |
| **Dropping and catching a ball-both hands** | | | | | | |
| Model 1 | Dropping & Catching | 1.10 | 1.74 | 0.63 | 0.14 | .53 |
|  | **IQ** | **0.37** | **0.10** | **3.61** | **0.79** | **<.001** |
| Model 2 | Dropping & Catching | 1.80 | 1.76 | 1.03 | 0.23 | .31 |
|  | **SEQ** | **-0.10** | **0.03** | **-2.92** | **0.64** | **.004** |
| **Dribbling a ball-alternating hands** | | | | | | |
| Model 1 | Dribbling a ball | 2.37 | 2.08 | 1.14 | 0.25 | .26 |
|  | **IQ** | **0.34** | **0.11** | **3.22** | **0.71** | **.002** |
| Model 2 | Dribbling a ball | 3.48 | 2.05 | 1.70 | 0.37 | .09 |
|  | **SEQ** | **-0.09** | **0.03** | **-2.65** | **0.58** | **.01** |
| **Jumping in place-same sides synchronized** | | | | | | |
| Model 1 | Jumping in place | 0.42 | 1.58 | 0.27 | 0.06 | .79 |
|  | **IQ** | **0.37** | **0.11** | **3.39** | **0.74** | **.001** |
| Model 2 | Jumping in place | 1.16 | 1.58 | 0.74 | 0.16 | .46 |
|  | **SEQ** | **-0.10** | **0.04** | **-2.63** | **0.58** | **.01** |
| **Standing on one leg on a balance beam-eyes open** | | | | | | |
| Model 1 | Standing on one leg | 2.73 | 1.62 | 1.69 | 0.37 | .10 |
|  | **IQ** | **0.34** | **0.10** | **3.35** | **0.74** | **.001** |
| Model 2 | Standing on one leg | 2.96 | 1.66 | 1.78 | 0.39 | .08 |
|  | **SEQ** | **-0.09** | **0.04** | **-2.53** | **0.56** | **.01** |
| **One-legged stationary hop** | | | | | | |
| Model 1 | Hopping-one leg | -0.07 | 1.53 | -0.05 | 0.01 | .96 |
|  | **IQ** | **0.38** | **0.10** | **3.70** | **0.81** | **<.001** |
| Model 2 | Hopping-one leg | 0.03 | 1.58 | 0.02 | <.001 | .99 |
|  | **SEQ** | **-0.10** | **0.04** | **-2.90** | **0.64** | **.01** |
| **Push-ups** | | | | | | |
| Model 1 | Push-ups | 0.38 | 2.19 | 0.17 | 0.04 | .86 |
|  | **IQ** | **0.38** | **0.10** | **3.66** | **0.80** | **<.001** |
| Model 2 | Push-ups | 1.97 | 2.20 | 0.89 | 0.20 | .37 |
|  | **SEQ** | **-0.10** | **0.03** | **-3.01** | **0.66** | **.003** |
| **Sit-ups** | | | | | | |
| Model 1 | Sit-ups | -0.96 | 1.84 | -0.52 | 0.11 | .60 |
|  | **IQ** | **0.39** | **0.10** | **3.81** | **0.84** | **<.001** |
| Model 2 | Sit-ups | -0.08 | 1.87 | -0.04 | 0.01 | .97 |
|  | **SEQ** | **-0.10** | **0.03** | **-2.99** | **0.66** | **.004** |

Note: IQ = intelligence quotient; WASI-2 = Wechsler Abbreviated Scales of Intelligence, 2^nd^ edition; SEQ = Sensory Experiences Questionnaire; BOT-2 = Bruininks-Oseretsky Test of Motor Proficiency, 2^nd^ edition.
