# Supplementary material for: The relation between specific motor skills and daily living skills in autistic children and adolescents
Source: Front Integr Neurosci. 2024 May 22;18:1334241. doi: 10.3389/fnint.2024.1334241 (PMC11150622; doi:10.3389/fnint.2024.1334241)
Supplement: Supplementary file 2 [file Data_Sheet_1.PDF]

The Relation Between Specific Motor Skills and Daily Living Skills in Autistic Children and Adolescents

Emily Skaletski

2024-02-12

```
library(psych)
library(corrplot)
```

```
## corrplot 0.92 loaded
```

```
library(Hmisc)
```

```
##
## Attaching package: 'Hmisc'
```

```
## The following object is masked from 'package:psych':
##
##     describe
```

```
## The following objects are masked from 'package:base':
##
##     format.pval, units
```

```
library(patchwork)
library(ggplot2)
```

```
##
## Attaching package: 'ggplot2'
```

```
## The following objects are masked from 'package:psych':
##
##     %+%, alpha
```

```
library(effectsize)
```

```
##
## Attaching package: 'effectsize'
```

```
## The following object is masked from 'package:psych':
##
##     phi
```

```
MotorDLS$BOT2_shorttotal_mot_sscore[MotorDLS$subject=="H020"] <- MotorDLS$BOT2_shorttotal_mot_sscore[MotorDLS$subject=="B009"]

MotorDLS_ASD <- subset(MotorDLS, MotorDLS$ASDGroup=="1" & MotorDLS$VinelandVersion=="VABS-2")

#Removing participants who were in two studies less than two months apart or did not complete measures and therefore cannot be imputed.
MotorDLS_ASD2 <- subset(MotorDLS_ASD, MotorDLS_ASD$subject!="VGML_28" & MotorDLS_ASD$subject!="H028" & MotorDLS_ASD$subject!="VGML_1" &MotorDLS_ASD$subject!="H026" & MotorDLS_ASD$subject!="H027" & MotorDLS_ASD$subject!="H088" & MotorDLS_ASD$subject!="H094" & MotorDLS_ASD$subject!="N047" & MotorDLS_ASD$subject!="VGML_2" & MotorDLS_ASD$subject!="VGML_24" & MotorDLS_ASD$subject!="N019")

#Uniformly coding push-up types.
MotorDLS_ASD$Pushup_type[MotorDLS_ASD$Pushups=="Full" | MotorDLS_ASD$Pushups=="full push-ups" | MotorDLS_ASD$Pushups=="Full Push-Ups" | MotorDLS_ASD$Pushups=="Full Push-ups"]<-"Full"
MotorDLS_ASD$Pushup_type[MotorDLS_ASD$Pushups=="Knee" | MotorDLS_ASD$Pushups=="knee push-ups" | MotorDLS_ASD$Pushups=="Knee Push-Ups" | MotorDLS_ASD$Pushups=="Knee Push-ups"]<-"Knee"

#The following lines of code are to address that some items have two trials. We want to use the highest score.
MotorDLS_ASD2$Penny_max <- pmax(MotorDLS_ASD2$Penny_Trial1_Raw_Score, MotorDLS_ASD2$Penny_Trial2_Raw_Score, na.rm=TRUE)
MotorDLS_ASD2$Jumping_max <- pmax(MotorDLS_ASD2$Jumping_Trial1_Raw_Score, MotorDLS_ASD2$Jumping_Trial2_Raw_Score, na.rm=TRUE)
MotorDLS_ASD2$Tapping_max <- pmax(MotorDLS_ASD2$Tapping_Trial1_Raw_Score, MotorDLS_ASD2$Tapping_Trial2_Raw_Score, na.rm=TRUE)
MotorDLS_ASD2$Walking_max <- pmax(MotorDLS_ASD2$Walking_forward_Trial1_Raw, MotorDLS_ASD2$Walking_forward_Trial2_Raw, na.rm=TRUE)
MotorDLS_ASD2$Standing_max <- pmax(MotorDLS_ASD2$Standing_One_Leg_Beam_Eyes_Open_Trial1_Raw, MotorDLS_ASD2$Standing_One_Leg_Beam_Eyes_Open_Trial2_Raw, na.rm=TRUE)
MotorDLS_ASD2$Hopping_max <- pmax(MotorDLS_ASD2$Hopping_Trial1_Raw_Score, MotorDLS_ASD2$Hopping_Trial2_Raw_Score, na.rm=TRUE)
MotorDLS_ASD2$Dribbling_max <- pmax(MotorDLS_ASD2$Dribbling_Trial1_Raw_Score, MotorDLS_ASD2$Dribbling_Trial2_Raw_Score, na.rm=TRUE)
MotorDLS_ASD2$IQ<- pmax(MotorDLS_ASD2$Composite_FSIQ.2, MotorDLS_ASD2$Composite_FSIQ.4, MotorDLS_ASD2$kbit_iqstand_score, na.rm=TRUE)
MotorDLS_ASD2$CopySquareStarSum<-MotorDLS_ASD2$CopySquare_Raw_Score+MotorDLS_ASD2$CopyStar_Raw_Score
MotorDLS_ASD2$Tapping_max <- as.numeric(MotorDLS_ASD2$Tapping_max)

#Organizing ADOS variables for demographics table.
MotorDLS_ASD2$ADOS_RRB_Total <- as.numeric(MotorDLS_ASD2$ADOS_RRB_Total)
```

```
## Warning: NAs introduced by coercion
```

```
MotorDLS_ASD2$ADOS2_M3_Total <- MotorDLS_ASD2$ADOS_SA_Total + MotorDLS_ASD2$ADOS_RRB_Total
MotorDLS_ASD2$ADOS2_M4_Total <- MotorDLS_ASD2$ADOS_Comm_Total + MotorDLS_ASD2$ADOS_Social_Interaction_Total + MotorDLS_ASD2$ADOS_Stereotyped_Bx_and_RR_Total

###Subset just of these columns.
MotorDLS_ASD3<-MotorDLS_ASD2[,c("subject", "Longitudinal_ID", "Grade", "Age_Acc","Sex..b.1..g.0.", "Hispanic", "New_ethnicity","IQ",  "SRSTscore", "SCQ_Total_Raw_Score", "ADOS2_M3_Total", "ADOS2_M4_Total", "BOT2_shorttotal_mot_sscore", "Daily_Live_Skills_standard", "Drawing_Point_Score", "Folding_Raw_Score", "CopySquareStarSum", "Penny_max", "Jumping_max", "Tapping_max", "Walking_max", "Standing_max", "Hopping_max", "Catching_Raw_Score", "Dribbling_max", "Pushup_Raw_Score", "Situps_Raw_Score", "BalanceTscore")]
```

```
table(MotorDLS_ASD3$Sex..b.1..g.0.)
```

```
##
##  0  1
## 11 79
```

```
table(MotorDLS_ASD3$Hispanic)
```

```
##
##  0  1
## 84  6
```

```
table(MotorDLS_ASD3$New_ethnicity)
```

```
##
##  1  2  4  5  6
##  1  5  6 73  5
```

```
describe(MotorDLS_ASD3$Age_Acc)
```

```
## MotorDLS_ASD3$Age_Acc
##        n  missing distinct     Info     Mean      Gmd      .05      .10
##       90        0       89        1    11.58    3.983    6.491    7.199
##      .25      .50      .75      .90      .95
##    8.910   10.488   14.840   16.204   17.037
##
## lowest : 6.22142 6.39357 6.39909 6.41273 6.4769
## highest: 17.1381 17.2379 17.3267 17.5082 17.847
```

```
describe(MotorDLS_ASD3$IQ)
```

```
## MotorDLS_ASD3$IQ
##        n  missing distinct     Info     Mean      Gmd      .05      .10
##       90        0       41    0.999    102.3    18.99     73.0     77.8
##      .25      .50      .75      .90      .95
##     94.0    104.5    113.5    121.0    128.3
##
## lowest :  62  67  72  73  75, highest: 123 125 131 135 136
```

```
describe(MotorDLS_ASD3$SRSTscore)
```

```
## MotorDLS_ASD3$SRSTscore
##        n  missing distinct     Info     Mean      Gmd      .05      .10
##       90        0       33    0.997    75.17    11.74    54.45    62.00
##      .25      .50      .75      .90      .95
##    68.50    76.00    83.75    90.00    90.00
##
## lowest : 48 51 53 54 55, highest: 86 87 88 89 90
```

```
describe(MotorDLS_ASD3$SCQ_Total_Raw_Score) #1 missing is due to age of adoption
```

```
## MotorDLS_ASD3$SCQ_Total_Raw_Score
##        n  missing distinct     Info     Mean      Gmd      .05      .10
##       89        1       25    0.998    18.96    8.344      8.0      8.8
##      .25      .50      .75      .90      .95
##     15.0     19.0     24.0     28.2     30.0
##
## lowest :  6  7  8  9 10, highest: 28 29 30 33 37
```

```
describe(MotorDLS_ASD3$BOT2_shorttotal_mot_sscore) #1 missing, did not complete short form, just Balance.
```

```
## MotorDLS_ASD3$BOT2_shorttotal_mot_sscore
##        n  missing distinct     Info     Mean      Gmd      .05      .10
##       89        1       25    0.996    38.45    7.254     30.0     31.0
##      .25      .50      .75      .90      .95
##     34.0     38.0     41.0     45.2     51.2
##
## lowest : 24 27 28 30 31, highest: 50 52 53 61 68
```

```
describe(MotorDLS_ASD3$BalanceTscore) #1 missing, did not complete Balance section, just short form.
```

```
## MotorDLS_ASD3$BalanceTscore
##        n  missing distinct     Info     Mean      Gmd      .05      .10
##       89        1       21    0.992     10.2    5.121      4.4      5.0
##      .25      .50      .75      .90      .95
##      6.0     10.0     12.0     16.2     19.8
##
## lowest :  2  3  4  5  6, highest: 18 21 22 23 24
```

```
describe(MotorDLS_ASD3$Daily_Live_Skills_standard)
```

```
## MotorDLS_ASD3$Daily_Live_Skills_standard
##        n  missing distinct     Info     Mean      Gmd      .05      .10
##       90        0       39    0.998    88.64    18.61     66.0     68.0
##      .25      .50      .75      .90      .95
##     76.0     89.0    101.8    110.0    114.6
##
## lowest :  54  55  63  65  66, highest: 112 113 116 117 121
```

```
describe(MotorDLS_ASD3$ADOS2_M3_Total)
```

```
## MotorDLS_ASD3$ADOS2_M3_Total
##        n  missing distinct     Info     Mean      Gmd      .05      .10
##       55       35       21    0.994    12.64    5.809      4.7      6.4
##      .25      .50      .75      .90      .95
##      9.0     12.0     16.0     18.6     21.3
##
## lowest :  3  4  5  6  7, highest: 19 21 22 25 27
```

```
describe(MotorDLS_ASD3$ADOS2_M4_Total)
```

```
## MotorDLS_ASD3$ADOS2_M4_Total
##        n  missing distinct     Info     Mean      Gmd      .05      .10
##       35       55       16    0.993    13.43    6.094      5.2      8.4
##      .25      .50      .75      .90      .95
##     10.0     13.0     17.0     20.0     20.6
##
## Value          1     7     8     9    10    11    12    13    15    16    17
## Frequency      2     1     1     2     5     2     3     4     3     2     3
## Proportion 0.057 0.029 0.029 0.057 0.143 0.057 0.086 0.114 0.086 0.057 0.086
##
## Value         18    19    20    22    28
## Frequency      1     1     3     1     1
## Proportion 0.029 0.029 0.086 0.029 0.029
##
## For the frequency table, variable is rounded to the nearest 0
```

```
t.test(MotorDLS_ASD2$BOT2_short_total~MotorDLS_ASD2$Sex..b.1..g.0.)
```

```
##
##  Welch Two Sample t-test
##
## data:  MotorDLS_ASD2$BOT2_short_total by MotorDLS_ASD2$Sex..b.1..g.0.
## t = 0.5256, df = 22.544, p-value = 0.6043
## alternative hypothesis: true difference in means between group 0 and group 1 is not equal to 0
## 95 percent confidence interval:
##  -3.961432  6.656071
## sample estimates:
## mean in group 0 mean in group 1
##        58.09091        56.74359
```

```
describeBy(MotorDLS_ASD2$BOT2_short_total, group=MotorDLS_ASD2$Sex..b.1..g.0.)
```

```
##
##  Descriptive statistics by group
## group: 0
##    vars  n  mean   sd median trimmed  mad min max range  skew kurtosis   se
## X1    1 11 58.09 6.88     60   58.56 5.93  46  66    20 -0.53    -1.35 2.07
## ---------------------------------------------------------
## group: 1
##    vars  n  mean    sd median trimmed   mad min max range  skew kurtosis   se
## X1    1 78 56.74 13.31     59   57.53 11.12  19  86    67 -0.61     0.17 1.51
```

```
t.test(MotorDLS_ASD2$Daily_Live_Skills_standard~MotorDLS_ASD2$Sex..b.1..g.0.)
```

```
##
##  Welch Two Sample t-test
##
## data:  MotorDLS_ASD2$Daily_Live_Skills_standard by MotorDLS_ASD2$Sex..b.1..g.0.
## t = -1.5082, df = 12.281, p-value = 0.1568
## alternative hypothesis: true difference in means between group 0 and group 1 is not equal to 0
## 95 percent confidence interval:
##  -20.752528   3.749075
## sample estimates:
## mean in group 0 mean in group 1
##        81.18182        89.68354
```

```
t.test(MotorDLS_ASD2$BOT2_short_total~MotorDLS_ASD2$Hispanic)
```

```
##
##  Welch Two Sample t-test
##
## data:  MotorDLS_ASD2$BOT2_short_total by MotorDLS_ASD2$Hispanic
## t = -1.8217, df = 6.7304, p-value = 0.113
## alternative hypothesis: true difference in means between group 0 and group 1 is not equal to 0
## 95 percent confidence interval:
##  -15.900924   2.125823
## sample estimates:
## mean in group 0 mean in group 1
##        56.44578        63.33333
```

```
chisq.test(MotorDLS_ASD2$BOT2_short_total, y=MotorDLS_ASD2$Ethnicity)
```

```
## Warning in chisq.test(MotorDLS_ASD2$BOT2_short_total, y =
## MotorDLS_ASD2$Ethnicity): Chi-squared approximation may be incorrect
```

```
##
##  Pearson's Chi-squared test
##
## data:  MotorDLS_ASD2$BOT2_short_total and MotorDLS_ASD2$Ethnicity
## X-squared = 250.22, df = 320, p-value = 0.9985
```

```
t.test(MotorDLS_ASD2$Daily_Live_Skills_standard~MotorDLS_ASD2$Hispanic)
```

```
##
##  Welch Two Sample t-test
##
## data:  MotorDLS_ASD2$Daily_Live_Skills_standard by MotorDLS_ASD2$Hispanic
## t = -1.7456, df = 5.6543, p-value = 0.1345
## alternative hypothesis: true difference in means between group 0 and group 1 is not equal to 0
## 95 percent confidence interval:
##  -29.910072   5.219596
## sample estimates:
## mean in group 0 mean in group 1
##        87.82143       100.16667
```

```
chisq.test(MotorDLS_ASD2$Daily_Live_Skills_standard, y=MotorDLS_ASD2$Ethnicity)
```

```
## Warning in chisq.test(MotorDLS_ASD2$Daily_Live_Skills_standard, y =
## MotorDLS_ASD2$Ethnicity): Chi-squared approximation may be incorrect
```

```
##
##  Pearson's Chi-squared test
##
## data:  MotorDLS_ASD2$Daily_Live_Skills_standard and MotorDLS_ASD2$Ethnicity
## X-squared = 274.3, df = 304, p-value = 0.8886
```

```
cor.test(MotorDLS_ASD2$Age_Acc, as.numeric(MotorDLS_ASD2$Grade))
```

```
##
##  Pearson's product-moment correlation
##
## data:  MotorDLS_ASD2$Age_Acc and as.numeric(MotorDLS_ASD2$Grade)
## t = 56.859, df = 88, p-value < 2.2e-16
## alternative hypothesis: true correlation is not equal to 0
## 95 percent confidence interval:
##  0.9797651 0.9912184
## sample estimates:
##       cor
## 0.9866619
```

```
table(MotorDLS_ASD2$Grade)
```

```
##
##  0  1 10 11 12  2  3  4  5  7  8  9
##  3  8 13  5  2  8  8 16  9  6  6  6
```

```
Draw1 <- ggplot(MotorDLS_ASD2, aes(x=MotorDLS_ASD2$Drawing_Point_Score, y=Daily_Live_Skills_standard))+
  geom_point(size=.75)+
  geom_smooth(alpha=.2)+
 theme_bw()+
  theme(axis.title = element_text(face="bold", size=10))+
 theme(legend.position = "none")

Fold1 <- ggplot(MotorDLS_ASD2, aes(x=MotorDLS_ASD2$Folding_Raw_Score, y=Daily_Live_Skills_standard))+
  geom_point(size=.75)+
  geom_smooth(alpha=.2)+
 theme_bw()+
  theme(axis.title = element_text(face="bold", size=10))+
 theme(legend.position = "none")

Shape1 <- ggplot(MotorDLS_ASD2, aes(x=MotorDLS_ASD2$CopySquareStarSum, y=Daily_Live_Skills_standard))+
  geom_point(size=.75)+
  geom_smooth(alpha=.2)+
 theme_bw()+
  theme(axis.title = element_text(face="bold", size=10))+
 theme(legend.position = "none")

Penny1 <- ggplot(MotorDLS_ASD2, aes(x=MotorDLS_ASD2$Penny_max, y=Daily_Live_Skills_standard))+
  geom_point(size=.75)+
  geom_smooth(alpha=.2)+
 theme_bw()+
  theme(axis.title = element_text(face="bold", size=10))+
 theme(legend.position = "none")

Jump1 <- ggplot(MotorDLS_ASD2, aes(x=MotorDLS_ASD2$Jumping_max, y=Daily_Live_Skills_standard))+
  geom_point(size=.75)+
  geom_smooth(alpha=.2)+
 theme_bw()+
  theme(axis.title = element_text(face="bold", size=10))+
 theme(legend.position = "none")

Tap1 <- ggplot(MotorDLS_ASD2, aes(x=MotorDLS_ASD2$Tapping_max, y=Daily_Live_Skills_standard))+
  geom_point(size=.75)+
  geom_smooth(alpha=.2)+
 theme_bw()+
  theme(axis.title = element_text(face="bold", size=10))+
 theme(legend.position = "none")

Walk1 <- ggplot(MotorDLS_ASD2, aes(x=MotorDLS_ASD2$Walking_max, y=Daily_Live_Skills_standard))+
  geom_point(size=.75)+
  geom_smooth(alpha=.2)+
 theme_bw()+
  theme(axis.title = element_text(face="bold", size=10))+
 theme(legend.position = "none")

Stand1 <- ggplot(MotorDLS_ASD2, aes(x=MotorDLS_ASD2$Standing_max, y=Daily_Live_Skills_standard))+
  geom_point(size=.75)+
  geom_smooth(alpha=.2)+
 theme_bw()+
  theme(axis.title = element_text(face="bold", size=10))+
 theme(legend.position = "none")

Hop1 <- ggplot(MotorDLS_ASD2, aes(x=MotorDLS_ASD2$Hopping_max, y=Daily_Live_Skills_standard))+
  geom_point(size=.75)+
  geom_smooth(alpha=.2)+
 theme_bw()+
  theme(axis.title = element_text(face="bold", size=10))+
 theme(legend.position = "none")

Catch1 <- ggplot(MotorDLS_ASD2, aes(x=MotorDLS_ASD2$Catching_Raw_Score, y=Daily_Live_Skills_standard))+
  geom_point(size=.75)+
  geom_smooth(alpha=.2)+
 theme_bw()+
  theme(axis.title = element_text(face="bold", size=10))+
 theme(legend.position = "none")
```

```
Dribble1 <- ggplot(MotorDLS_ASD2, aes(x=MotorDLS_ASD2$Dribbling_max, y=Daily_Live_Skills_standard))+
  geom_point(size=.75)+
  geom_smooth(alpha=.2)+
 theme_bw()+
  theme(axis.title = element_text(face="bold", size=10))+
 theme(legend.position = "none")

Pushup1 <- ggplot(MotorDLS_ASD2, aes(x=MotorDLS_ASD2$Pushup_Raw_Score, y=Daily_Live_Skills_standard))+
  geom_point(size=.75)+
  geom_smooth(alpha=.2)+
 theme_bw()+
  theme(axis.title = element_text(face="bold", size=10))+
 theme(legend.position = "none")

Situp1 <- ggplot(MotorDLS_ASD2, aes(x=MotorDLS_ASD2$Situps_Raw_Score, y=Daily_Live_Skills_standard))+
  geom_point(size=.75)+
  geom_smooth(alpha=.2)+
 theme_bw()+
  theme(axis.title = element_text(face="bold", size=10))+
 theme(legend.position = "none")

Draw1 + Fold1 + Shape1 + Penny1 + Jump1 + Tap1 + Walk1 + Stand1 + Hop1 + Catch1 + Dribble1 + Pushup1 + Situp1
```

```
## `geom_smooth()` using method = 'loess' and formula = 'y ~ x'
```

```
## Warning: Removed 1 rows containing non-finite values (`stat_smooth()`).
```

```
## Warning in simpleLoess(y, x, w, span, degree = degree, parametric = parametric,
## : pseudoinverse used at 7.03
```

```
## Warning in simpleLoess(y, x, w, span, degree = degree, parametric = parametric,
## : neighborhood radius 1.03
```

```
## Warning in simpleLoess(y, x, w, span, degree = degree, parametric = parametric,
## : reciprocal condition number 0
```

```
## Warning in simpleLoess(y, x, w, span, degree = degree, parametric = parametric,
## : There are other near singularities as well. 1
```

```
## Warning in predLoess(object$y, object$x, newx = if (is.null(newdata)) object$x
## else if (is.data.frame(newdata))
## as.matrix(model.frame(delete.response(terms(object)), : pseudoinverse used at
## 7.03
```

```
## Warning in predLoess(object$y, object$x, newx = if (is.null(newdata)) object$x
## else if (is.data.frame(newdata))
## as.matrix(model.frame(delete.response(terms(object)), : neighborhood radius
## 1.03
```

```
## Warning in predLoess(object$y, object$x, newx = if (is.null(newdata)) object$x
## else if (is.data.frame(newdata))
## as.matrix(model.frame(delete.response(terms(object)), : reciprocal condition
## number 0
```

```
## Warning in predLoess(object$y, object$x, newx = if (is.null(newdata)) object$x
## else if (is.data.frame(newdata))
## as.matrix(model.frame(delete.response(terms(object)), : There are other near
## singularities as well. 1
```

```
## Warning: Removed 1 rows containing missing values (`geom_point()`).
```

```
## `geom_smooth()` using method = 'loess' and formula = 'y ~ x'
```

```
## Warning: Removed 1 rows containing non-finite values (`stat_smooth()`).
## Removed 1 rows containing missing values (`geom_point()`).
```

```
## `geom_smooth()` using method = 'loess' and formula = 'y ~ x'
```

```
## Warning: Removed 1 rows containing non-finite values (`stat_smooth()`).
```

```
## Warning in simpleLoess(y, x, w, span, degree = degree, parametric = parametric,
## : pseudoinverse used at 10.05
```

```
## Warning in simpleLoess(y, x, w, span, degree = degree, parametric = parametric,
## : neighborhood radius 1.05
```

```
## Warning in simpleLoess(y, x, w, span, degree = degree, parametric = parametric,
## : reciprocal condition number 0
```

```
## Warning in simpleLoess(y, x, w, span, degree = degree, parametric = parametric,
## : There are other near singularities as well. 1
```

```
## Warning in predLoess(object$y, object$x, newx = if (is.null(newdata)) object$x
## else if (is.data.frame(newdata))
## as.matrix(model.frame(delete.response(terms(object)), : pseudoinverse used at
## 10.05
```

```
## Warning in predLoess(object$y, object$x, newx = if (is.null(newdata)) object$x
## else if (is.data.frame(newdata))
## as.matrix(model.frame(delete.response(terms(object)), : neighborhood radius
## 1.05
```

```
## Warning in predLoess(object$y, object$x, newx = if (is.null(newdata)) object$x
## else if (is.data.frame(newdata))
## as.matrix(model.frame(delete.response(terms(object)), : reciprocal condition
## number 0
```

```
## Warning in predLoess(object$y, object$x, newx = if (is.null(newdata)) object$x
## else if (is.data.frame(newdata))
## as.matrix(model.frame(delete.response(terms(object)), : There are other near
## singularities as well. 1
```

```
## Warning: Removed 1 rows containing missing values (`geom_point()`).
```

```
## `geom_smooth()` using method = 'loess' and formula = 'y ~ x'
```

```
## Warning: Removed 1 rows containing non-finite values (`stat_smooth()`).
## Removed 1 rows containing missing values (`geom_point()`).
```

```
## `geom_smooth()` using method = 'loess' and formula = 'y ~ x'
```

```
## Warning: Removed 1 rows containing non-finite values (`stat_smooth()`).
```

```
## Warning in simpleLoess(y, x, w, span, degree = degree, parametric = parametric,
## : pseudoinverse used at 5.025
```

```
## Warning in simpleLoess(y, x, w, span, degree = degree, parametric = parametric,
## : neighborhood radius 2.025
```

```
## Warning in simpleLoess(y, x, w, span, degree = degree, parametric = parametric,
## : reciprocal condition number 3.0622e-16
```

```
## Warning in simpleLoess(y, x, w, span, degree = degree, parametric = parametric,
## : There are other near singularities as well. 1
```

```
## Warning in predLoess(object$y, object$x, newx = if (is.null(newdata)) object$x
## else if (is.data.frame(newdata))
## as.matrix(model.frame(delete.response(terms(object)), : pseudoinverse used at
## 5.025
```

```
## Warning in predLoess(object$y, object$x, newx = if (is.null(newdata)) object$x
## else if (is.data.frame(newdata))
## as.matrix(model.frame(delete.response(terms(object)), : neighborhood radius
## 2.025
```

```
## Warning in predLoess(object$y, object$x, newx = if (is.null(newdata)) object$x
## else if (is.data.frame(newdata))
## as.matrix(model.frame(delete.response(terms(object)), : reciprocal condition
## number 3.0622e-16
```

```
## Warning in predLoess(object$y, object$x, newx = if (is.null(newdata)) object$x
## else if (is.data.frame(newdata))
## as.matrix(model.frame(delete.response(terms(object)), : There are other near
## singularities as well. 1
```

```
## Warning: Removed 1 rows containing missing values (`geom_point()`).
```

```
## `geom_smooth()` using method = 'loess' and formula = 'y ~ x'
```

```
## Warning: Removed 1 rows containing non-finite values (`stat_smooth()`).
```

```
## Warning in simpleLoess(y, x, w, span, degree = degree, parametric = parametric,
## : at 10.05
```

```
## Warning in simpleLoess(y, x, w, span, degree = degree, parametric = parametric,
## : radius 0.0025
```

```
## Warning in simpleLoess(y, x, w, span, degree = degree, parametric = parametric,
## : all data on boundary of neighborhood. make span bigger
```

```
## Warning in simpleLoess(y, x, w, span, degree = degree, parametric = parametric,
## : pseudoinverse used at 10.05
```

```
## Warning in simpleLoess(y, x, w, span, degree = degree, parametric = parametric,
## : neighborhood radius 0.05
```

```
## Warning in simpleLoess(y, x, w, span, degree = degree, parametric = parametric,
## : reciprocal condition number 0.092827
```

```
## Warning in simpleLoess(y, x, w, span, degree = degree, parametric = parametric,
## : There are other near singularities as well. 1
```

```
## Warning in simpleLoess(y, x, w, span, degree = degree, parametric = parametric,
## : zero-width neighborhood. make span bigger
```

```
## Warning: Computation failed in `stat_smooth()`
## Caused by error in `predLoess()`:
## ! NA/NaN/Inf in foreign function call (arg 5)
```

```
## Warning: Removed 1 rows containing missing values (`geom_point()`).
```

```
## `geom_smooth()` using method = 'loess' and formula = 'y ~ x'
```

```
## Warning in simpleLoess(y, x, w, span, degree = degree, parametric = parametric,
## : at 6.03
```

```
## Warning in simpleLoess(y, x, w, span, degree = degree, parametric = parametric,
## : radius 0.0009
```

```
## Warning in simpleLoess(y, x, w, span, degree = degree, parametric = parametric,
## : all data on boundary of neighborhood. make span bigger
```

```
## Warning in simpleLoess(y, x, w, span, degree = degree, parametric = parametric,
## : pseudoinverse used at 6.03
```

```
## Warning in simpleLoess(y, x, w, span, degree = degree, parametric = parametric,
## : neighborhood radius 0.03
```

```
## Warning in simpleLoess(y, x, w, span, degree = degree, parametric = parametric,
## : reciprocal condition number 0.098921
```

```
## Warning in simpleLoess(y, x, w, span, degree = degree, parametric = parametric,
## : There are other near singularities as well. 1
```

```
## Warning in simpleLoess(y, x, w, span, degree = degree, parametric = parametric,
## : zero-width neighborhood. make span bigger
```

```
## Warning: Computation failed in `stat_smooth()`
## Caused by error in `predLoess()`:
## ! NA/NaN/Inf in foreign function call (arg 5)
```

```
## `geom_smooth()` using method = 'loess' and formula = 'y ~ x'
## `geom_smooth()` using method = 'loess' and formula = 'y ~ x'
```

```
## Warning: Removed 1 rows containing non-finite values (`stat_smooth()`).
```

```
## Warning: Removed 1 rows containing missing values (`geom_point()`).
```

```
## `geom_smooth()` using method = 'loess' and formula = 'y ~ x'
```

```
## Warning: Removed 1 rows containing non-finite values (`stat_smooth()`).
```

```
## Warning in simpleLoess(y, x, w, span, degree = degree, parametric = parametric,
## : pseudoinverse used at 5.025
```

```
## Warning in simpleLoess(y, x, w, span, degree = degree, parametric = parametric,
## : neighborhood radius 1.025
```

```
## Warning in simpleLoess(y, x, w, span, degree = degree, parametric = parametric,
## : reciprocal condition number 0
```

```
## Warning in simpleLoess(y, x, w, span, degree = degree, parametric = parametric,
## : There are other near singularities as well. 1
```

```
## Warning in predLoess(object$y, object$x, newx = if (is.null(newdata)) object$x
## else if (is.data.frame(newdata))
## as.matrix(model.frame(delete.response(terms(object)), : pseudoinverse used at
## 5.025
```

```
## Warning in predLoess(object$y, object$x, newx = if (is.null(newdata)) object$x
## else if (is.data.frame(newdata))
## as.matrix(model.frame(delete.response(terms(object)), : neighborhood radius
## 1.025
```

```
## Warning in predLoess(object$y, object$x, newx = if (is.null(newdata)) object$x
## else if (is.data.frame(newdata))
## as.matrix(model.frame(delete.response(terms(object)), : reciprocal condition
## number 0
```

```
## Warning in predLoess(object$y, object$x, newx = if (is.null(newdata)) object$x
## else if (is.data.frame(newdata))
## as.matrix(model.frame(delete.response(terms(object)), : There are other near
## singularities as well. 1
```

```
## Warning: Removed 1 rows containing missing values (`geom_point()`).
```

```
## `geom_smooth()` using method = 'loess' and formula = 'y ~ x'
```

```
## Warning: Removed 1 rows containing non-finite values (`stat_smooth()`).
## Removed 1 rows containing missing values (`geom_point()`).
```

```
## `geom_smooth()` using method = 'loess' and formula = 'y ~ x'
```

```
## Warning: Removed 1 rows containing non-finite values (`stat_smooth()`).
## Removed 1 rows containing missing values (`geom_point()`).
```

```
## `geom_smooth()` using method = 'loess' and formula = 'y ~ x'
```

```
## Warning: Removed 1 rows containing non-finite values (`stat_smooth()`).
## Removed 1 rows containing missing values (`geom_point()`).
```

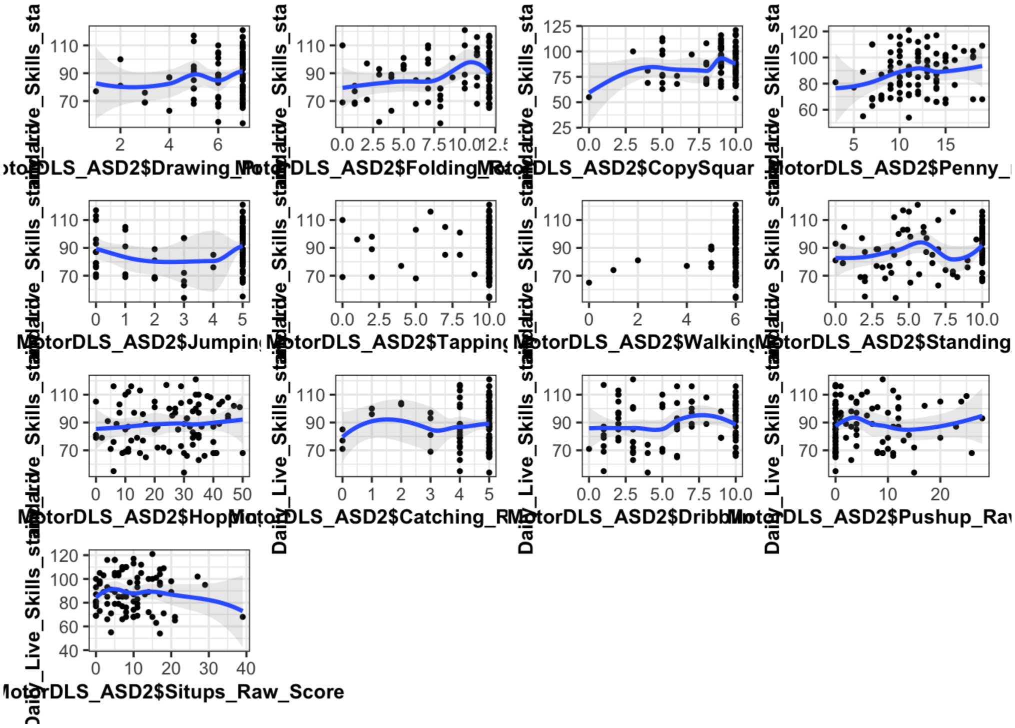

#We performed three sets of Spearman correlations (in light of potential non-normality and outliers) looking at individual BOT-2 SF items and DLS.

```
MotorDLS_ASD_A1 <- subset(MotorDLS_ASD3, MotorDLS_ASD3$subject!="H058")
MotorDLS_ASD_A1_sub<-MotorDLS_ASD_A1[,c( "Age_Acc", "IQ", "Daily_Live_Skills_standard", "Drawing_Point_Score", "F
olding_Raw_Score", "CopySquareStarSum", "Penny_max", "Jumping_max", "Standing_max", "Hopping_max", "Catching_Raw_
Score", "Dribbling_max", "Pushup_Raw_Score", "Situps_Raw_Score")]

#First with raw, unadjusted BOT-2 item scores.
res2<-rcorr(as.matrix(MotorDLS_ASD_A1_sub), type="spearman")
corrplot(res2$r, method="number", number.cex=.5)
```

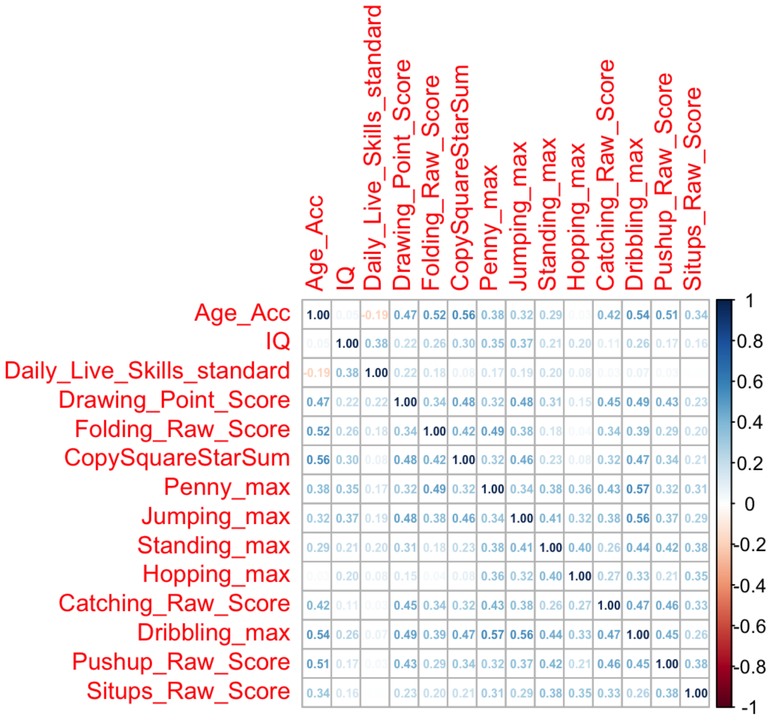

```
#Next, we will scale the items in advance of residualizing for age and IQ.
MotorDLS_ASD_A1$Drawscale <- scale(MotorDLS_ASD_A1$Drawing_Point_Score, center=TRUE, scale=TRUE)
MotorDLS_ASD_A1$Foldscale <- scale(MotorDLS_ASD_A1$Folding_Raw_Score, center=TRUE, scale=TRUE)
MotorDLS_ASD_A1$Shapescale <- scale(MotorDLS_ASD_A1$CopySquareStarSum, center=TRUE, scale=TRUE)
MotorDLS_ASD_A1$Pennyscale <- scale(MotorDLS_ASD_A1$Penny_max, center=TRUE, scale=TRUE)
MotorDLS_ASD_A1$Jumpscale <- scale(MotorDLS_ASD_A1$Jumping_max, center=TRUE, scale=TRUE)
MotorDLS_ASD_A1$Standscale <- scale(MotorDLS_ASD_A1$Standing_max, center=TRUE, scale=TRUE)
MotorDLS_ASD_A1$Hopscale <- scale(MotorDLS_ASD_A1$Hopping_max, center=TRUE, scale=TRUE)
MotorDLS_ASD_A1$Catchscale <- scale(MotorDLS_ASD_A1$Catching_Raw_Score, center=TRUE, scale=TRUE)
MotorDLS_ASD_A1$Dribblescale <- scale(MotorDLS_ASD_A1$Dribbling_max, center=TRUE, scale=TRUE)
MotorDLS_ASD_A1$Pushupscale <- scale(MotorDLS_ASD_A1$Pushup_Raw_Score, center=TRUE, scale=TRUE)
MotorDLS_ASD_A1$Situpscale <- scale(MotorDLS_ASD_A1$Situps_Raw_Score, center=TRUE, scale=TRUE)
MotorDLS_ASD_A1$DLSscale <- scale(MotorDLS_ASD_A1$Daily_Live_Skills_standard, center=TRUE, scale=TRUE)

#Now, we will look at the relations between DLS and BOT-2 SF items residualized for age.
MotorDLS_ASD_A1$DLSscale.residage <- summary(lm(data=MotorDLS_ASD_A1, DLSscale~ Age_Acc))$resid
MotorDLS_ASD_A1$Standscale.residage <- summary(lm(data=MotorDLS_ASD_A1, Standscale~ Age_Acc))$resid
MotorDLS_ASD_A1$Dribblescale.residage <- summary(lm(data=MotorDLS_ASD_A1, Dribblescale~ Age_Acc))$resid
MotorDLS_ASD_A1$Hopscale.residage <- summary(lm(data=MotorDLS_ASD_A1, Hopscale~ Age_Acc))$resid
MotorDLS_ASD_A1$Drawscale.residage <- summary(lm(data=MotorDLS_ASD_A1, Drawscale~ Age_Acc))$resid
MotorDLS_ASD_A1$Foldscale.residage <- summary(lm(data=MotorDLS_ASD_A1, Foldscale~ Age_Acc))$resid
MotorDLS_ASD_A1$Jumpscale.residage <- summary(lm(data=MotorDLS_ASD_A1, Jumpscale~ Age_Acc))$resid
MotorDLS_ASD_A1$Shapescale.residage <- summary(lm(data=MotorDLS_ASD_A1, Shapescale~ Age_Acc))$resid
MotorDLS_ASD_A1$Pennyscale.residage <- summary(lm(data=MotorDLS_ASD_A1, Pennyscale~ Age_Acc))$resid
MotorDLS_ASD_A1$Pushupscale.residage <- summary(lm(data=MotorDLS_ASD_A1, Pushupscale~ Age_Acc))$resid
MotorDLS_ASD_A1$Situpscale.residage <- summary(lm(data=MotorDLS_ASD_A1, Situpscale~ Age_Acc))$resid
MotorDLS_ASD_A1$Catchscale.residage <- summary(lm(data=MotorDLS_ASD_A1, Catchscale~ Age_Acc))$resid

MotorDLS_ASD_A1_subage<-MotorDLS_ASD_A1[,c("DLSscale.residage", "Drawscale.residage", "Foldscale.residage", "Shap
escale.residage", "Pennyscale.residage", "Jumpscale.residage", "Standscale.residage", "Hopscale.residage", "Catch
scale.residage", "Dribblescale.residage", "Pushupscale.residage", "Situpscale.residage")]
res2age<-rcorr(as.matrix(MotorDLS_ASD_A1_subage), type="spearman")
corrplot(res2age$r, method="number", number.cex=.5)
```

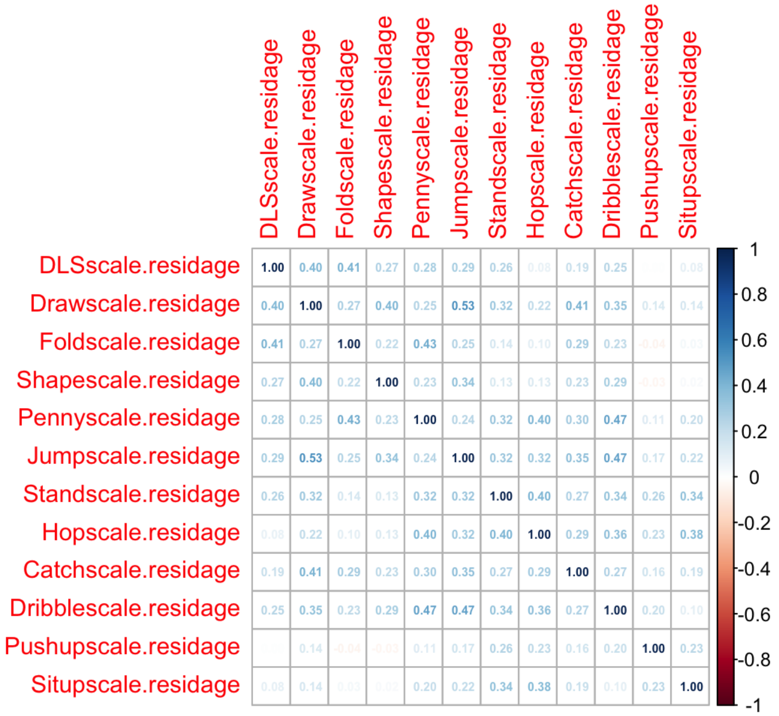

```
#Last, we will look at relatiosn between DLS and BOT-2 residualized by IQ.
MotorDLS_ASD_A1$DLSscale.residIQ <- summary(lm(data=MotorDLS_ASD_A1, DLSscale~IQ))$resid
MotorDLS_ASD_A1$Standscale.residIQ <- summary(lm(data=MotorDLS_ASD_A1, Standscale~IQ))$resid
MotorDLS_ASD_A1$Dribblescale.residIQ <- summary(lm(data=MotorDLS_ASD_A1, Dribblescale~IQ))$resid
MotorDLS_ASD_A1$Hopscale.residIQ <- summary(lm(data=MotorDLS_ASD_A1, Hopscale~IQ))$resid
MotorDLS_ASD_A1$Drawscale.residIQ <- summary(lm(data=MotorDLS_ASD_A1, Drawscale~IQ))$resid
MotorDLS_ASD_A1$Foldscale.residIQ <- summary(lm(data=MotorDLS_ASD_A1, Foldscale~IQ))$resid
MotorDLS_ASD_A1$Jumpscale.residIQ <- summary(lm(data=MotorDLS_ASD_A1, Jumpscale~IQ))$resid
MotorDLS_ASD_A1$Shapescale.residIQ <- summary(lm(data=MotorDLS_ASD_A1, Shapescale~IQ))$resid
MotorDLS_ASD_A1$Pennyscale.residIQ <- summary(lm(data=MotorDLS_ASD_A1, Pennyscale~IQ))$resid
MotorDLS_ASD_A1$Pushupscale.residIQ <- summary(lm(data=MotorDLS_ASD_A1, Pushupscale~ IQ))$resid
MotorDLS_ASD_A1$Situpscale.residIQ <- summary(lm(data=MotorDLS_ASD_A1, Situpscale~IQ))$resid
MotorDLS_ASD_A1$Catchscale.residIQ <- summary(lm(data=MotorDLS_ASD_A1, Catchscale~IQ))$resid

MotorDLS_ASD_A1_subIQ<-MotorDLS_ASD_A1[,c("DLSscale.residIQ", "Drawscale.residIQ", "Foldscale.residIQ", "Shapescale.residIQ", "Pennyscale.residIQ", "Jumpscale.residIQ", "Standscale.residIQ", "Hopscale.residIQ", "Catchscale.residIQ", "Dribblescale.residIQ", "Pushupscale.residIQ", "Situpscale.residIQ")]
res2IQ<-rcorr(as.matrix(MotorDLS_ASD_A1_subIQ), type="spearman")
corrplot(res2IQ$r, method="number", number.cex=.5)
```

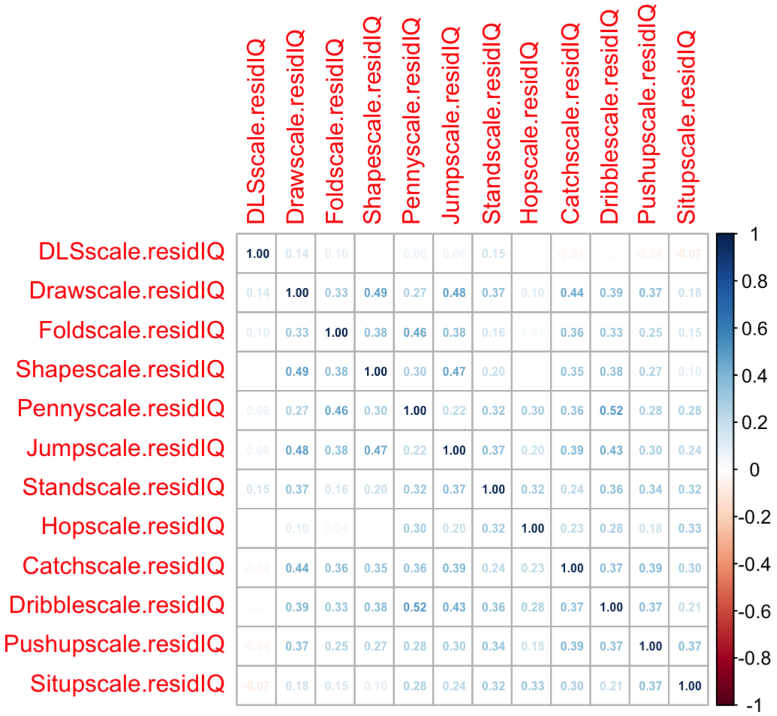

#Next, we developed age-standardized scores for each BOT-2 SF item.

```
MotorDLS$Penny_max <- pmax(MotorDLS$Penny_Trial1_Raw_Score, MotorDLS$Penny_Trial2_Raw_Score, na.rm=TRUE)
MotorDLS$Jumping_max <- pmax(MotorDLS$Jumping_Trial1_Raw_Score, MotorDLS$Jumping_Trial2_Raw_Score, na.rm=TRUE)
MotorDLS$Standing_max <- pmax(MotorDLS$Standing_One_Leg_Beam_Eyes_Open_Trial1_Raw, MotorDLS$Standing_One_Leg_Beam
_Eyes_Open_Trial2_Raw, na.rm=TRUE)
MotorDLS$Hopping_max <- pmax(MotorDLS$Hopping_Trial1_Raw_Score, MotorDLS$Hopping_Trial2_Raw_Score, na.rm=TRUE)
MotorDLS$Dribbling_max <- pmax(MotorDLS$Dribbling_Trial1_Raw_Score, MotorDLS$Dribbling_Trial2_Raw_Score, na.rm=TR
UE)
MotorDLS$IQ<- pmax(MotorDLS$Composite_FSIQ.2, MotorDLS$Composite_FSIQ.4, MotorDLS$kbit_iqstand_score, na.rm=TRUE)
MotorDLS$CopySquareStarSum<-MotorDLS$CopySquare_Raw_Score+MotorDLS$CopyStar_Raw_Score

Draw <- ggplot(MotorDLS, aes(x=Age_Acc, y=Drawing_Point_Score, colour=as.factor(ASDGroup)))+
  geom_point(size=.75)+
  geom_smooth(alpha=.2)+
   theme_bw()+
 theme_bw()+
  theme(axis.title = element_text(face="bold", size=10))+
 theme(legend.position = "none")

Fold <- ggplot(MotorDLS, aes(x=Age_Acc, y=Folding_Raw_Score, colour=as.factor(ASDGroup)))+
  geom_point(size=.75)+
  geom_smooth(alpha=.2)+
   theme_bw()+
 theme_bw()+
  theme(axis.title = element_text(face="bold", size=10))+
 theme(legend.position = "none")

Shapes <- ggplot(MotorDLS, aes(x=Age_Acc, y=CopySquareStarSum, colour=as.factor(ASDGroup)))+
  geom_point(size=.75)+
  geom_smooth(alpha=.2)+
   theme_bw()+
 theme_bw()+
  theme(axis.title = element_text(face="bold", size=10))+
 theme(legend.position = "none")

Pennies <- ggplot(MotorDLS, aes(x=Age_Acc, y=Penny_max, colour=as.factor(ASDGroup)))+
  geom_point(size=.75)+
  geom_smooth(alpha=.2)+
   theme_bw()+
 theme_bw()+
  theme(axis.title = element_text(face="bold", size=10))+
 theme(legend.position = "none")

Jumping <- ggplot(MotorDLS, aes(x=Age_Acc, y=Jumping_max, colour=as.factor(ASDGroup)))+
  geom_point(size=.75)+
  geom_smooth(alpha=.2)+
   theme_bw()+
 theme_bw()+
  theme(axis.title = element_text(face="bold", size=10))+
 theme(legend.position = "none")

Standing <- ggplot(MotorDLS, aes(x=Age_Acc, y=Standing_max, colour=as.factor(ASDGroup)))+
  geom_point(size=.75)+
  geom_smooth(alpha=.2)+
   theme_bw()+
 theme_bw()+
  theme(axis.title = element_text(face="bold", size=10))+
 theme(legend.position = "none")

Hopping <- ggplot(MotorDLS, aes(x=Age_Acc, y=Hopping_max, colour=as.factor(ASDGroup)))+
  geom_point(size=.75)+
  geom_smooth(alpha=.2)+
   theme_bw()+
 theme_bw()+
  theme(axis.title = element_text(face="bold", size=10))+
 theme(legend.position = "none")

Catching <- ggplot(MotorDLS, aes(x=Age_Acc, y=Catching_Raw_Score, colour=as.factor(ASDGroup)))+
  geom_point(size=.75)+
  geom_smooth(alpha=.2)+
```

```
   theme_bw()+
 theme_bw()+
  theme(axis.title = element_text(face="bold", size=10))+
 theme(legend.position = "none")

Dribbling <- ggplot(MotorDLS, aes(x=Age_Acc, y=Dribbling_max, colour=as.factor(ASDGroup)))+
  geom_point(size=.75)+
  geom_smooth(alpha=.2)+
   theme_bw()+
 theme_bw()+
  theme(axis.title = element_text(face="bold", size=10))+
 theme(legend.position = "none")

Pushups <- ggplot(MotorDLS, aes(x=Age_Acc, y=Pushup_Raw_Score, colour=as.factor(ASDGroup)))+
  geom_point(size=.75)+
  geom_smooth(alpha=.2)+
   theme_bw()+
 theme_bw()+
  theme(axis.title = element_text(face="bold", size=10))+
 theme(legend.position = "none")

Situps <- ggplot(MotorDLS, aes(x=Age_Acc, y=Situps_Raw_Score, colour=as.factor(ASDGroup)))+
  geom_point(size=.75)+
  geom_smooth(alpha=.2)+
   theme_bw()+
 theme_bw()+
  theme(axis.title = element_text(face="bold", size=10))+
 theme(legend.position = "none")

DLS <- ggplot(MotorDLS, aes(x=Age_Acc, y=Daily_Live_Skills_standard, colour=as.factor(ASDGroup)))+
  geom_point(size=.75)+
  geom_smooth(alpha=.2)+
   theme_bw()+
 theme_bw()+
  theme(axis.title = element_text(face="bold", size=10))+
 theme(legend.position = "none")

Draw + Fold + Shapes + Pennies + Jumping + Standing + Hopping + Catching + Dribbling + Pushups + Situps + DLS
```

```
## `geom_smooth()` using method = 'loess' and formula = 'y ~ x'
```

```
## Warning: Removed 41 rows containing non-finite values (`stat_smooth()`).
```

```
## Warning: Removed 41 rows containing missing values (`geom_point()`).
```

```
## `geom_smooth()` using method = 'loess' and formula = 'y ~ x'
```

```
## Warning: Removed 41 rows containing non-finite values (`stat_smooth()`).
## Removed 41 rows containing missing values (`geom_point()`).
```

```
## `geom_smooth()` using method = 'loess' and formula = 'y ~ x'
```

```
## Warning: Removed 41 rows containing non-finite values (`stat_smooth()`).
## Removed 41 rows containing missing values (`geom_point()`).
```

```
## `geom_smooth()` using method = 'loess' and formula = 'y ~ x'
```

```
## Warning: Removed 41 rows containing non-finite values (`stat_smooth()`).
## Removed 41 rows containing missing values (`geom_point()`).
```

```
## `geom_smooth()` using method = 'loess' and formula = 'y ~ x'
```

```
## Warning: Removed 41 rows containing non-finite values (`stat_smooth()`).
## Removed 41 rows containing missing values (`geom_point()`).
```

```
## `geom_smooth()` using method = 'loess' and formula = 'y ~ x'
```

```
## Warning: Removed 47 rows containing non-finite values (`stat_smooth()`).
```

```
## Warning: Removed 47 rows containing missing values (`geom_point()`).
```

```
## `geom_smooth()` using method = 'loess' and formula = 'y ~ x'
```

```
## Warning: Removed 41 rows containing non-finite values (`stat_smooth()`).
```

```
## Warning: Removed 41 rows containing missing values (`geom_point()`).
```

```
## `geom_smooth()` using method = 'loess' and formula = 'y ~ x'
```

```
## Warning: Removed 41 rows containing non-finite values (`stat_smooth()`).
## Removed 41 rows containing missing values (`geom_point()`).
```

```
## `geom_smooth()` using method = 'loess' and formula = 'y ~ x'
```

```
## Warning: Removed 41 rows containing non-finite values (`stat_smooth()`).
## Removed 41 rows containing missing values (`geom_point()`).
```

```
## `geom_smooth()` using method = 'loess' and formula = 'y ~ x'
```

```
## Warning: Removed 41 rows containing non-finite values (`stat_smooth()`).
## Removed 41 rows containing missing values (`geom_point()`).
```

```
## `geom_smooth()` using method = 'loess' and formula = 'y ~ x'
```

```
## Warning: Removed 41 rows containing non-finite values (`stat_smooth()`).
## Removed 41 rows containing missing values (`geom_point()`).
```

```
## `geom_smooth()` using method = 'loess' and formula = 'y ~ x'
```

```
## Warning: Removed 92 rows containing non-finite values (`stat_smooth()`).
```

```
## Warning: Removed 92 rows containing missing values (`geom_point()`).
```

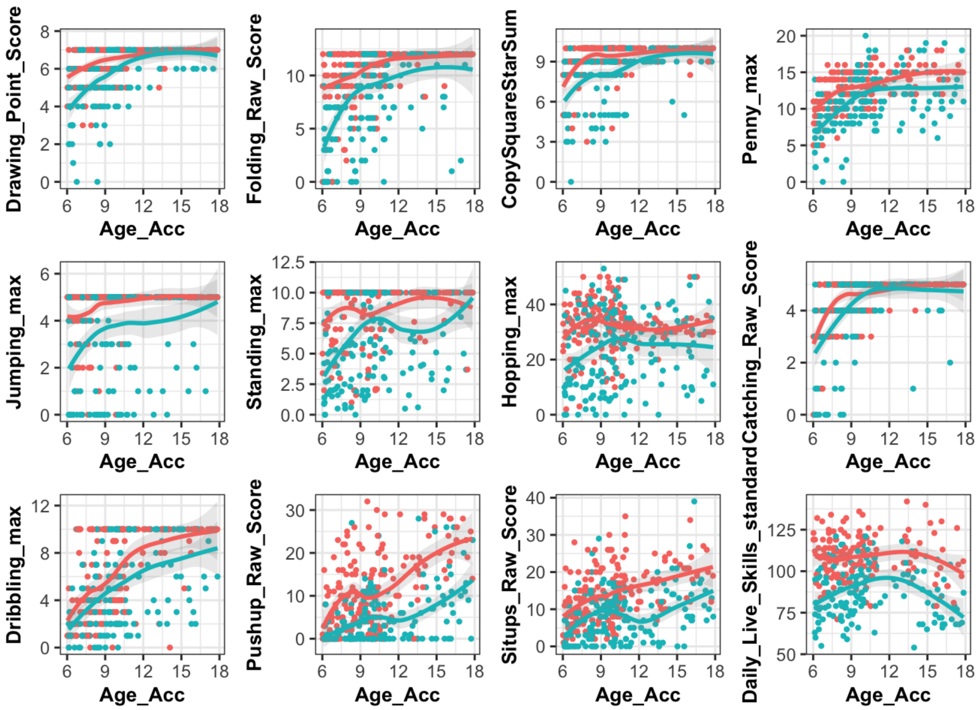
Drawing_Point_Score
Folding_Raw_Score
CopySquareStarSum
Penny_max
Jumping_max
Standing_max
Hopping_max
Catching_Raw_Score
Dribbling_max
Pushup_Raw_Score
Situps_Raw_Score
Daily_Live_Skills_standard
Age_Acc

```
#The relation between the items and age appears to be log fit.
MotorDLS$Agelog <- log(MotorDLS$Age_Acc)

Draw2 <- ggplot(MotorDLS, aes(x=Age_Acc, y=Drawing_Point_Score))+
  geom_point(size=.75)+
  geom_smooth(alpha=.2)+
   stat_smooth(method = "lm", formula = y ~ log(x), colour="purple")+ #log
   theme_bw()+
 theme_bw()+
  theme(axis.title = element_text(face="bold", size=10))+
 theme(legend.position = "none")

Fold2 <- ggplot(MotorDLS, aes(x=Age_Acc, y=Folding_Raw_Score))+
  geom_point(size=.75)+
  geom_smooth(alpha=.2)+
   stat_smooth(method = "lm", formula = y ~ log(x), colour="purple")+ #log
   theme_bw()+
 theme_bw()+
  theme(axis.title = element_text(face="bold", size=10))+
 theme(legend.position = "none")

Shapes2 <- ggplot(MotorDLS, aes(x=Age_Acc, y=CopySquareStarSum))+
  geom_point(size=.75)+
  geom_smooth(alpha=.2)+
   stat_smooth(method = "lm", formula = y ~ log(x), colour="purple")+ #log
   theme_bw()+
 theme_bw()+
  theme(axis.title = element_text(face="bold", size=10))+
 theme(legend.position = "none")

Pennies2 <- ggplot(MotorDLS, aes(x=Age_Acc, y=Penny_max))+
  geom_point(size=.75)+
  geom_smooth(alpha=.2)+
   stat_smooth(method = "lm", formula = y ~ log(x), colour="purple")+ #log
   theme_bw()+
 theme_bw()+
  theme(axis.title = element_text(face="bold", size=10))+
 theme(legend.position = "none")

Jumping2 <- ggplot(MotorDLS, aes(x=Age_Acc, y=Jumping_max))+
  geom_point(size=.75)+
  geom_smooth(alpha=.2)+
   stat_smooth(method = "lm", formula = y ~ log(x), colour="purple")+ #log
   theme_bw()+
 theme_bw()+
  theme(axis.title = element_text(face="bold", size=10))+
 theme(legend.position = "none")

Standing2 <- ggplot(MotorDLS, aes(x=Age_Acc, y=Standing_max))+
  geom_point(size=.75)+
  geom_smooth(alpha=.2)+
   stat_smooth(method = "lm", formula = y ~ log(x), colour="purple")+ #log
   theme_bw()+
 theme_bw()+
  theme(axis.title = element_text(face="bold", size=10))+
 theme(legend.position = "none")

Hopping2 <- ggplot(MotorDLS, aes(x=Age_Acc, y=Hopping_max))+
  geom_point(size=.75)+
  geom_smooth(alpha=.2)+
   stat_smooth(method = "lm", formula = y ~ log(x), colour="purple")+ #log
   theme_bw()+
 theme_bw()+
  theme(axis.title = element_text(face="bold", size=10))+
 theme(legend.position = "none")

Catching2 <- ggplot(MotorDLS, aes(x=Age_Acc, y=Catching_Raw_Score))+
  geom_point(size=.75)+
  geom_smooth(alpha=.2)+
```

```
   stat_smooth(method = "lm", formula = y ~ log(x), colour="purple")+ #log
   theme_bw()+
 theme_bw()+
  theme(axis.title = element_text(face="bold", size=10))+
 theme(legend.position = "none")

Dribbling2 <- ggplot(MotorDLS, aes(x=Age_Acc, y=Dribbling_max))+
  geom_point(size=.75)+
  geom_smooth(alpha=.2)+
   stat_smooth(method = "lm", formula = y ~ log(x), colour="purple")+ #log
   theme_bw()+
 theme_bw()+
  theme(axis.title = element_text(face="bold", size=10))+
 theme(legend.position = "none")

Pushups2 <- ggplot(MotorDLS, aes(x=Age_Acc, y=Pushup_Raw_Score))+
  geom_point(size=.75)+
  geom_smooth(alpha=.2)+
   stat_smooth(method = "lm", formula = y ~ log(x), colour="purple")+ #log
   theme_bw()+
 theme_bw()+
  theme(axis.title = element_text(face="bold", size=10))+
 theme(legend.position = "none")

Situps2 <- ggplot(MotorDLS, aes(x=Age_Acc, y=Situps_Raw_Score))+
  geom_point(size=.75)+
  geom_smooth(alpha=.2)+
   stat_smooth(method = "lm", formula = y ~ log(x), colour="purple")+ #log
   theme_bw()+
 theme_bw()+
  theme(axis.title = element_text(face="bold", size=10))+
 theme(legend.position = "none")

DLS2 <- ggplot(MotorDLS, aes(x=Age_Acc, y=Daily_Live_Skills_standard))+
  geom_point(size=.75)+
  geom_smooth(alpha=.2)+
   stat_smooth(method = "lm", formula = y ~ log(x), colour="purple")+ #log
   theme_bw()+
 theme_bw()+
  theme(axis.title = element_text(face="bold", size=10))+
 theme(legend.position = "none")

Draw2 + Fold2 + Shapes2 + Pennies2 + Jumping2 + Standing2 + Hopping2 + Catching2 + Dribbling2 + Pushups2 + Situps
2 + DLS2
```

```
## `geom_smooth()` using method = 'loess' and formula = 'y ~ x'
```

```
## Warning: Removed 41 rows containing non-finite values (`stat_smooth()`).
```

```
## Warning: Removed 41 rows containing non-finite values (`stat_smooth()`).
```

```
## Warning: Removed 41 rows containing missing values (`geom_point()`).
```

```
## `geom_smooth()` using method = 'loess' and formula = 'y ~ x'
```

```
## Warning: Removed 41 rows containing non-finite values (`stat_smooth()`).
```

```
## Warning: Removed 41 rows containing non-finite values (`stat_smooth()`).
```

```
## Warning: Removed 41 rows containing missing values (`geom_point()`).
```

```
## `geom_smooth()` using method = 'loess' and formula = 'y ~ x'
```

```
## Warning: Removed 41 rows containing non-finite values (`stat_smooth()`).
```

```
## Warning: Removed 41 rows containing non-finite values (`stat_smooth()`).
```

```
## Warning: Removed 41 rows containing missing values (`geom_point()`).
```

```
## `geom_smooth()` using method = 'loess' and formula = 'y ~ x'
```

```
## Warning: Removed 41 rows containing non-finite values (`stat_smooth()`).
```

```
## Warning: Removed 41 rows containing non-finite values (`stat_smooth()`).
```

```
## Warning: Removed 41 rows containing missing values (`geom_point()`).
```

```
## `geom_smooth()` using method = 'loess' and formula = 'y ~ x'
```

```
## Warning: Removed 41 rows containing non-finite values (`stat_smooth()`).
```

```
## Warning: Removed 41 rows containing non-finite values (`stat_smooth()`).
```

```
## Warning: Removed 41 rows containing missing values (`geom_point()`).
```

```
## `geom_smooth()` using method = 'loess' and formula = 'y ~ x'
```

```
## Warning: Removed 47 rows containing non-finite values (`stat_smooth()`).
```

```
## Warning: Removed 47 rows containing non-finite values (`stat_smooth()`).
```

```
## Warning: Removed 47 rows containing missing values (`geom_point()`).
```

```
## `geom_smooth()` using method = 'loess' and formula = 'y ~ x'
```

```
## Warning: Removed 41 rows containing non-finite values (`stat_smooth()`).
```

```
## Warning: Removed 41 rows containing non-finite values (`stat_smooth()`).
```

```
## Warning: Removed 41 rows containing missing values (`geom_point()`).
```

```
## `geom_smooth()` using method = 'loess' and formula = 'y ~ x'
```

```
## Warning: Removed 41 rows containing non-finite values (`stat_smooth()`).
```

```
## Warning: Removed 41 rows containing non-finite values (`stat_smooth()`).
```

```
## Warning: Removed 41 rows containing missing values (`geom_point()`).
```

```
## `geom_smooth()` using method = 'loess' and formula = 'y ~ x'
```

```
## Warning: Removed 41 rows containing non-finite values (`stat_smooth()`).
```

```
## Warning: Removed 41 rows containing non-finite values (`stat_smooth()`).
```

```
## Warning: Removed 41 rows containing missing values (`geom_point()`).
```

```
## `geom_smooth()` using method = 'loess' and formula = 'y ~ x'
```

```
## Warning: Removed 41 rows containing non-finite values (`stat_smooth()`).
```

```
## Warning: Removed 41 rows containing non-finite values (`stat_smooth()`).
```

```
## Warning: Removed 41 rows containing missing values (`geom_point()`).
```

```
## `geom_smooth()` using method = 'loess' and formula = 'y ~ x'
```

```
## Warning: Removed 41 rows containing non-finite values (`stat_smooth()`).
```

```
## Warning: Removed 41 rows containing non-finite values (`stat_smooth()`).
```

```
## Warning: Removed 41 rows containing missing values (`geom_point()`).
```

```
## `geom_smooth()` using method = 'loess' and formula = 'y ~ x'
```

```
## Warning: Removed 92 rows containing non-finite values (`stat_smooth()`).
```

```
## Warning: Removed 92 rows containing non-finite values (`stat_smooth()`).
```

```
## Warning: Removed 92 rows containing missing values (`geom_point()`).
```

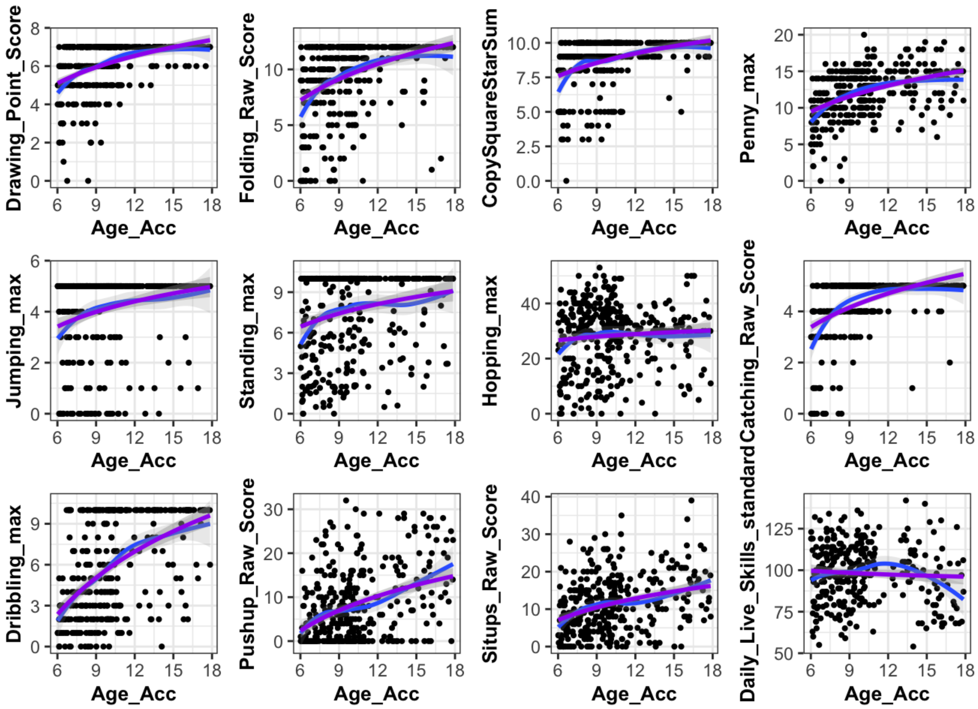

```
#Now, we need to residualize by age_log using the whole sample.
Drawing <- subset(MotorDLS, is.na(MotorDLS$Drawing_Point_Score)==FALSE)
Folding <- subset(MotorDLS, is.na(MotorDLS$Folding_Raw_Score)==FALSE)
Shapes <- subset(MotorDLS, is.na(MotorDLS$CopySquareStarSum)==FALSE)
Penny <- subset(MotorDLS, is.na(MotorDLS$Drawing_Point_Score)==FALSE)
Jump <- subset(MotorDLS, is.na(MotorDLS$Jumping_max)==FALSE)
Stand <- subset(MotorDLS, is.na(MotorDLS$Standing_max)==FALSE)
Hop <- subset(MotorDLS, is.na(MotorDLS$Hopping_max)==FALSE)
Catch <- subset(MotorDLS, is.na(MotorDLS$Catching_Raw_Score)==FALSE)
Dribble <- subset(MotorDLS, is.na(MotorDLS$Dribbling_max)==FALSE)
Pushup <- subset(MotorDLS, is.na(MotorDLS$Pushup_Raw_Score)==FALSE)
Situp <- subset(MotorDLS, is.na(MotorDLS$Situps_Raw_Score)==FALSE)

Drawing$Drawscale <- scale(Drawing$Drawing_Point_Score, center=TRUE, scale=TRUE)
Folding$Foldscale <- scale(Folding$Folding_Raw_Score, center=TRUE, scale=TRUE)
Shapes$Shapescale <- scale(Shapes$CopySquareStarSum, center=TRUE, scale=TRUE)
Penny$Pennyscale <- scale(Penny$Penny_max, center=TRUE, scale=TRUE)
Jump$Jumpscale <- scale(Jump$Jumping_max, center=TRUE, scale=TRUE)
Stand$Standscale <- scale(Stand$Standing_max, center=TRUE, scale=TRUE)
Hop$Hopscale <- scale(Hop$Hopping_max, center=TRUE, scale=TRUE)
Catch$Catchscale <- scale(Catch$Catching_Raw_Score, center=TRUE, scale=TRUE)
Dribble$Dribblescale <- scale(Dribble$Dribbling_max, center=TRUE, scale=TRUE)
Pushup$Pushupscale <- scale(Pushup$Pushup_Raw_Score, center=TRUE, scale=TRUE)
Situp$Situpscale <- scale(Situp$Situps_Raw_Score, center=TRUE, scale=TRUE)

Drawing$Drawscale.resid <- summary(lm(data=Drawing, Drawscale~ Agelog))$resid
Folding$Foldscale.resid <- summary(lm(data=Folding, Foldscale~ Agelog))$resid
Shapes$Shapescale.resid <- summary(lm(data=Shapes, Shapescale~ Agelog))$resid
Penny$Pennyscale.resid <- summary(lm(data=Penny, Pennyscale~ Agelog))$resid
Jump$Jumpscale.resid <- summary(lm(data=Jump, Jumpscale~ Agelog))$resid
Stand$Standscale.resid <- summary(lm(data=Stand, Standscale~ Agelog))$resid
Hop$Hopscale.resid <- summary(lm(data=Hop, Hopscale~ Agelog))$resid
Catch$Catchscale.resid <- summary(lm(data=Catch, Catchscale~ Agelog))$resid
Dribble$Dribblescale.resid <- summary(lm(data=Dribble, Dribblescale~ Agelog))$resid
Pushup$Pushupscale.resid <- summary(lm(data=Pushup, Pushupscale~ Agelog))$resid
Situp$Situpscale.resid <- summary(lm(data=Situp, Situpscale~ Agelog))$resid

Drawingage <- ggplot(Drawing, aes(x=Drawscale.resid, y=Daily_Live_Skills_standard))+
  geom_point(size=.75)+
  geom_smooth(alpha=.2)+
 theme_bw()+
  theme(axis.title = element_text(face="bold", size=10))+
 theme(legend.position = "none")

Foldingage <- ggplot(Folding, aes(x=Foldscale.resid, y=Daily_Live_Skills_standard))+
  geom_point(size=.75)+
  geom_smooth(alpha=.2)+
 theme_bw()+
  theme(axis.title = element_text(face="bold", size=10))+
 theme(legend.position = "none")

Shapesage <- ggplot(Shapes, aes(x=Shapescale.resid, y=Daily_Live_Skills_standard))+
  geom_point(size=.75)+
  geom_smooth(alpha=.2)+
 theme_bw()+
  theme(axis.title = element_text(face="bold", size=10))+
 theme(legend.position = "none")

Pennyage <- ggplot(Penny, aes(x=Pennyscale.resid, y=Daily_Live_Skills_standard))+
  geom_point(size=.75)+
  geom_smooth(alpha=.2)+
 theme_bw()+
  theme(axis.title = element_text(face="bold", size=10))+
 theme(legend.position = "none")

Jumpage <- ggplot(Jump, aes(x=Jumpscale.resid, y=Daily_Live_Skills_standard))+
  geom_point(size=.75)+
  geom_smooth(alpha=.2)+
 theme_bw()+
```

```
  theme(axis.title = element_text(face="bold", size=10))+
 theme(legend.position = "none")

Standage <- ggplot(Stand, aes(x=Standscale.resid, y=Daily_Live_Skills_standard))+
  geom_point(size=.75)+
  geom_smooth(alpha=.2)+
 theme_bw()+
  theme(axis.title = element_text(face="bold", size=10))+
 theme(legend.position = "none")

Hopage <- ggplot(Hop, aes(x=Hopscale.resid, y=Daily_Live_Skills_standard))+
  geom_point(size=.75)+
  geom_smooth(alpha=.2)+
 theme_bw()+
  theme(axis.title = element_text(face="bold", size=10))+
 theme(legend.position = "none")

Catchage <- ggplot(Catch, aes(x=Catchscale.resid, y=Daily_Live_Skills_standard))+
  geom_point(size=.75)+
  geom_smooth(alpha=.2)+
 theme_bw()+
  theme(axis.title = element_text(face="bold", size=10))+
 theme(legend.position = "none")

Dribbleage <- ggplot(Dribble, aes(x=Dribblescale.resid, y=Daily_Live_Skills_standard))+
  geom_point(size=.75)+
  geom_smooth(alpha=.2)+
 theme_bw()+
  theme(axis.title = element_text(face="bold", size=10))+
 theme(legend.position = "none")

Pushupage <- ggplot(Pushup, aes(x=Pushupscale.resid, y=Daily_Live_Skills_standard))+
  geom_point(size=.75)+
  geom_smooth(alpha=.2)+
 theme_bw()+
  theme(axis.title = element_text(face="bold", size=10))+
 theme(legend.position = "none")

Situpage <- ggplot(Situp, aes(x=Situpscale.resid, y=Daily_Live_Skills_standard))+
  geom_point(size=.75)+
  geom_smooth(alpha=.2)+
 theme_bw()+
  theme(axis.title = element_text(face="bold", size=10))+
 theme(legend.position = "none")

Drawingage + Foldingage + Shapesage + Pennyage + Jumpage + Standage + Hopage + Catchage + Dribbleage + Pushupage
+ Situpage
```

```
## `geom_smooth()` using method = 'loess' and formula = 'y ~ x'
```

```
## Warning: Removed 55 rows containing non-finite values (`stat_smooth()`).
```

```
## Warning: Removed 55 rows containing missing values (`geom_point()`).
```

```
## `geom_smooth()` using method = 'loess' and formula = 'y ~ x'
```

```
## Warning: Removed 55 rows containing non-finite values (`stat_smooth()`).
## Removed 55 rows containing missing values (`geom_point()`).
```

```
## `geom_smooth()` using method = 'loess' and formula = 'y ~ x'
```

```
## Warning: Removed 55 rows containing non-finite values (`stat_smooth()`).
## Removed 55 rows containing missing values (`geom_point()`).
```

```
## `geom_smooth()` using method = 'loess' and formula = 'y ~ x'
```

```
## Warning: Removed 55 rows containing non-finite values (`stat_smooth()`).
## Removed 55 rows containing missing values (`geom_point()`).
```

```
## `geom_smooth()` using method = 'loess' and formula = 'y ~ x'
```

```
## Warning: Removed 55 rows containing non-finite values (`stat_smooth()`).
## Removed 55 rows containing missing values (`geom_point()`).
```

```
## `geom_smooth()` using method = 'loess' and formula = 'y ~ x'
```

```
## Warning: Removed 55 rows containing non-finite values (`stat_smooth()`).
## Removed 55 rows containing missing values (`geom_point()`).
```

```
## `geom_smooth()` using method = 'loess' and formula = 'y ~ x'
```

```
## Warning: Removed 55 rows containing non-finite values (`stat_smooth()`).
## Removed 55 rows containing missing values (`geom_point()`).
```

```
## `geom_smooth()` using method = 'loess' and formula = 'y ~ x'
```

```
## Warning: Removed 55 rows containing non-finite values (`stat_smooth()`).
## Removed 55 rows containing missing values (`geom_point()`).
```

```
## `geom_smooth()` using method = 'loess' and formula = 'y ~ x'
```

```
## Warning: Removed 55 rows containing non-finite values (`stat_smooth()`).
## Removed 55 rows containing missing values (`geom_point()`).
```

```
## `geom_smooth()` using method = 'loess' and formula = 'y ~ x'
```

```
## Warning: Removed 55 rows containing non-finite values (`stat_smooth()`).
## Removed 55 rows containing missing values (`geom_point()`).
```

```
## `geom_smooth()` using method = 'loess' and formula = 'y ~ x'
```

```
## Warning: Removed 55 rows containing non-finite values (`stat_smooth()`).
## Removed 55 rows containing missing values (`geom_point()`).
```

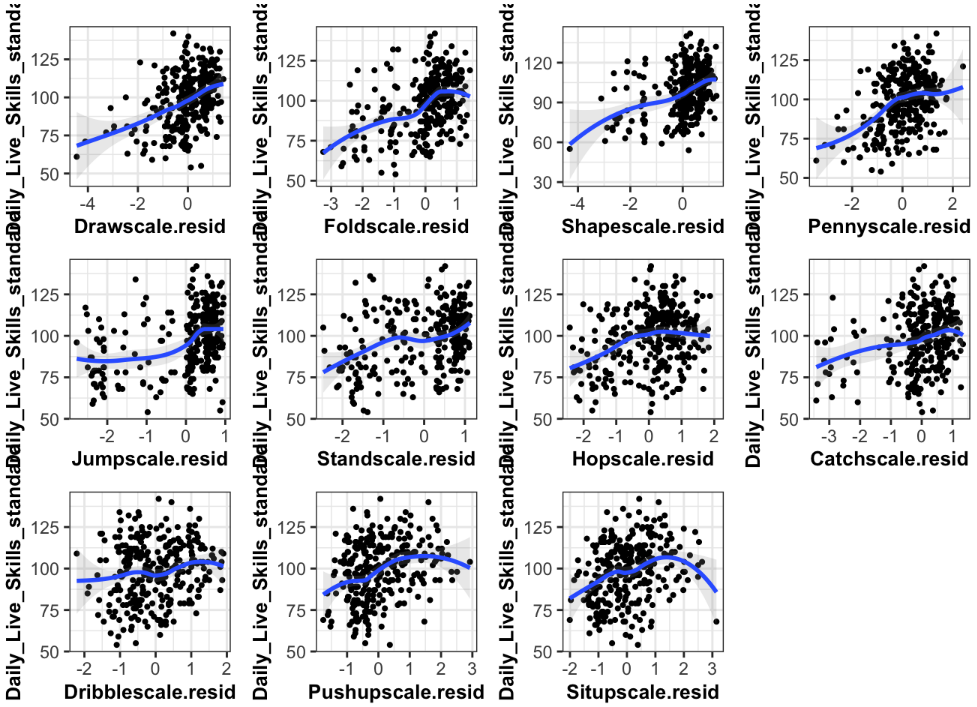


```
#Now that we have the scaled and residualized items, we will check the Spearman correlations.
cor.test(x=Drawing$Drawscale.resid, y=Drawing$Daily_Live_Skills_standard, method="spearman")
```

```
## Warning in cor.test.default(x = Drawing$Drawscale.resid, y =
## Drawing$Daily_Live_Skills_standard, : Cannot compute exact p-value with ties
```

```
##
##  Spearman's rank correlation rho
##
## data:  Drawing$Drawscale.resid and Drawing$Daily_Live_Skills_standard
## S = 2600423, p-value = 4.086e-13
## alternative hypothesis: true rho is not equal to 0
## sample estimates:
##       rho
## 0.4044328
```

```
cor.test(x=Folding$Foldscale.resid, y=Folding$Daily_Live_Skills_standard, method="spearman")
```

```
## Warning in cor.test.default(x = Folding$Foldscale.resid, y =
## Folding$Daily_Live_Skills_standard, : Cannot compute exact p-value with ties
```

```
##
##  Spearman's rank correlation rho
##
## data:  Folding$Foldscale.resid and Folding$Daily_Live_Skills_standard
## S = 2551053, p-value = 7.684e-14
## alternative hypothesis: true rho is not equal to 0
## sample estimates:
##       rho
## 0.4157399
```

```
cor.test(x=Shapes$Shapescale.resid, y=Shapes$Daily_Live_Skills_standard, method="spearman")
```

```
## Warning in cor.test.default(x = Shapes$Shapescale.resid, y =
## Shapes$Daily_Live_Skills_standard, : Cannot compute exact p-value with ties
```

```
##
##  Spearman's rank correlation rho
##
## data:  Shapes$Shapescale.resid and Shapes$Daily_Live_Skills_standard
## S = 2753388, p-value = 4.924e-11
## alternative hypothesis: true rho is not equal to 0
## sample estimates:
##       rho
## 0.3693997
```

```
cor.test(x=Penny$Pennyscale.resid, y=Penny$Daily_Live_Skills_standard, method="spearman")
```

```
## Warning in cor.test.default(x = Penny$Pennyscale.resid, y =
## Penny$Daily_Live_Skills_standard, : Cannot compute exact p-value with ties
```

```
##
##  Spearman's rank correlation rho
##
## data:  Penny$Pennyscale.resid and Penny$Daily_Live_Skills_standard
## S = 2959845, p-value = 1.348e-08
## alternative hypothesis: true rho is not equal to 0
## sample estimates:
##       rho
## 0.3221153
```

```
cor.test(x=Jump$Jumpscale.resid, y=Jump$Daily_Live_Skills_standard, method="spearman")
```

```
## Warning in cor.test.default(x = Jump$Jumpscale.resid, y =
## Jump$Daily_Live_Skills_standard, : Cannot compute exact p-value with ties
```

```
##
##  Spearman's rank correlation rho
##
## data:  Jump$Jumpscale.resid and Jump$Daily_Live_Skills_standard
## S = 2775337, p-value = 9.355e-11
## alternative hypothesis: true rho is not equal to 0
## sample estimates:
##       rho
## 0.3643727
```

```
cor.test(x=Stand$Standscale.resid, y=Stand$Daily_Live_Skills_standard, method="spearman")
```

```
## Warning in cor.test.default(x = Stand$Standscale.resid, y =
## Stand$Daily_Live_Skills_standard, : Cannot compute exact p-value with ties
```

```
##
##  Spearman's rank correlation rho
##
## data:  Stand$Standscale.resid and Stand$Daily_Live_Skills_standard
## S = 2868913, p-value = 1.582e-07
## alternative hypothesis: true rho is not equal to 0
## sample estimates:
##       rho
## 0.3014544
```

```
cor.test(x=Hop$Hopscale.resid, y=Hop$Daily_Live_Skills_standard, method="spearman")
```

```
## Warning in cor.test.default(x = Hop$Hopscale.resid, y =
## Hop$Daily_Live_Skills_standard, : Cannot compute exact p-value with ties
```

```
##
##  Spearman's rank correlation rho
##
## data:  Hop$Hopscale.resid and Hop$Daily_Live_Skills_standard
## S = 3339554, p-value = 4.259e-05
## alternative hypothesis: true rho is not equal to 0
## sample estimates:
##       rho
## 0.2351517
```

```
cor.test(x=Catch$Catchscale.resid, y=Catch$Daily_Live_Skills_standard, method="spearman")
```

```
## Warning in cor.test.default(x = Catch$Catchscale.resid, y =
## Catch$Daily_Live_Skills_standard, : Cannot compute exact p-value with ties
```

```
##
##  Spearman's rank correlation rho
##
## data:  Catch$Catchscale.resid and Catch$Daily_Live_Skills_standard
## S = 3359075, p-value = 5.996e-05
## alternative hypothesis: true rho is not equal to 0
## sample estimates:
##       rho
## 0.2306809
```

```
cor.test(x=Dribble$Dribblescale.resid, y=Dribble$Daily_Live_Skills_standard, method="spearman")
```

```
## Warning in cor.test.default(x = Dribble$Dribblescale.resid, y =
## Dribble$Daily_Live_Skills_standard, : Cannot compute exact p-value with ties
```

```
##
##  Spearman's rank correlation rho
##
## data:  Dribble$Dribblescale.resid and Dribble$Daily_Live_Skills_standard
## S = 3658764, p-value = 0.00512
## alternative hypothesis: true rho is not equal to 0
## sample estimates:
##      rho
## 0.162044
```

```
cor.test(x=Pushup$Pushupscale.resid, y=Pushup$Daily_Live_Skills_standard, method="spearman")
```

```
## Warning in cor.test.default(x = Pushup$Pushupscale.resid, y =
## Pushup$Daily_Live_Skills_standard, : Cannot compute exact p-value with ties
```

```
##
##  Spearman's rank correlation rho
##
## data:  Pushup$Pushupscale.resid and Pushup$Daily_Live_Skills_standard
## S = 3024860, p-value = 6.534e-08
## alternative hypothesis: true rho is not equal to 0
## sample estimates:
##       rho
## 0.3072252
```

```
cor.test(x=Situp$Situpscale.resid, y=Situp$Daily_Live_Skills_standard, method="spearman")
```

```
## Warning in cor.test.default(x = Situp$Situpscale.resid, y =
## Situp$Daily_Live_Skills_standard, : Cannot compute exact p-value with ties
```

```
##
##  Spearman's rank correlation rho
##
## data:  Situp$Situpscale.resid and Situp$Daily_Live_Skills_standard
## S = 3071367, p-value = 1.918e-07
## alternative hypothesis: true rho is not equal to 0
## sample estimates:
##       rho
## 0.2965738
```

#Aim 1: How do specific motor skills on the BOT-2 Short Form relate to DLS?

```
MotorDLS_ASD4 <-subset(MotorDLS_ASD3, MotorDLS_ASD3$subject!="H058")
d <- merge(MotorDLS_ASD4, Drawing[,c("subject", "Drawscale.resid")], by="subject", all.x = TRUE, all.y=FALSE)
d2 <- merge(d, Folding[,c("subject", "Foldscale.resid")], by="subject", all.x = TRUE, all.y=FALSE)
d3 <- merge(d2, Shapes[,c("subject", "Shapescale.resid")], by="subject", all.x = TRUE, all.y=FALSE)
d4 <- merge(d3, Penny[,c("subject", "Pennyscale.resid")], by="subject", all.x = TRUE, all.y=FALSE)
d5 <- merge(d4, Jump[,c("subject", "Jumpscale.resid")], by="subject", all.x = TRUE, all.y=FALSE)
d6 <- merge(d5, Stand[,c("subject", "Standscale.resid")], by="subject", all.x = TRUE, all.y=FALSE)
d7 <- merge(d6, Hop[,c("subject", "Hopscale.resid")], by="subject", all.x = TRUE, all.y=FALSE)
d8 <- merge(d7, Catch[,c("subject", "Catchscale.resid")], by="subject", all.x = TRUE, all.y=FALSE)
d9 <- merge(d8, Dribble[,c("subject", "Dribblescale.resid")], by="subject", all.x = TRUE, all.y=FALSE)
d10 <- merge(d9, Pushup[,c("subject", "Pushupscale.resid")], by="subject", all.x = TRUE, all.y=FALSE)
d11 <- merge(d10, Situp[,c("subject", "Situpscale.resid")], by="subject", all.x = TRUE, all.y=FALSE)

write.csv(d11, "MotorDLS_1.9.24.csv", row.names=FALSE)

d11sub<-d11[,c("Age_Acc", "IQ", "Daily_Live_Skills_standard", "Drawscale.resid", "Foldscale.resid", "Shapescale.r
esid", "Pennyscale.resid", "Jumpscale.resid", "Standscale.resid", "Hopscale.resid", "Catchscale.resid", "Dribbles
cale.resid", "Pushupscale.resid", "Situpscale.resid")]

res3<-rcorr(as.matrix(d11sub), type="spearman")
res3$P
```

```
##                              Age_Acc           IQ Daily_Live_Skills_standard
## Age_Acc                           NA 0.6578721679               6.862014e-02
## IQ                      0.6578721679           NA               2.820299e-04
## Daily_Live_Skills_standard 0.0686201414 0.0002820299                     NA
## Drawscale.resid         0.1283686410 0.0317890206               9.136716e-05
## Foldscale.resid         0.8252235571 0.0033129808               1.340769e-04
## Shapescale.resid        0.2174909198 0.0018733708               1.259584e-02
## Pennyscale.resid        0.2535462488 0.0002633812               7.782424e-03
## Jumpscale.resid         0.0535166024 0.0006005799               2.682999e-03
## Standscale.resid        0.6842120600 0.1151373563               9.985196e-03
## Hopscale.resid          0.7756498663 0.0507464477               3.554695e-01
## Catchscale.resid        0.0007428543 0.2835455620               3.148049e-02
## Dribblescale.resid      0.7150920716 0.0043380869               2.186525e-02
## Pushupscale.resid       0.4764825599 0.1556735623               8.543332e-01
## Situpscale.resid        0.6175846283 0.2447792974               5.217720e-01
##                         Drawscale.resid Foldscale.resid Shapescale.resid
## Age_Acc                    1.283686e-01    8.252236e-01     0.2174909198
## IQ                         3.178902e-02    3.312981e-03     0.0018733708
## Daily_Live_Skills_standard 9.136716e-05    1.340769e-04     0.0125958402
## Drawscale.resid                      NA    2.499110e-02     0.0007554091
## Foldscale.resid            2.499110e-02              NA     0.1449468578
## Shapescale.resid           7.554091e-04    1.449469e-01               NA
## Pennyscale.resid           2.704510e-02    8.455636e-05     0.0512804659
## Jumpscale.resid            1.731145e-07    4.697208e-02     0.0048679727
## Standscale.resid           6.830822e-03    3.094104e-01     0.5290914355
## Hopscale.resid             4.557298e-02    4.464380e-01     0.2665851990
## Catchscale.resid           3.763799e-05    6.839501e-03     0.0396223218
## Dribblescale.resid         8.404775e-04    5.871102e-02     0.0124398142
## Pushupscale.resid          2.831435e-01    7.319017e-01     0.9384093225
## Situpscale.resid           2.597901e-01    9.093584e-01     0.7559967988
##                         Pennyscale.resid Jumpscale.resid Standscale.resid
## Age_Acc                     2.535462e-01    5.351660e-02     6.842121e-01
## IQ                          2.633812e-04    6.005799e-04     1.151374e-01
## Daily_Live_Skills_standard  7.782424e-03    2.682999e-03     9.985196e-03
## Drawscale.resid             2.704510e-02    1.731145e-07     6.830822e-03
## Foldscale.resid             8.455636e-05    4.697208e-02     3.094104e-01
## Shapescale.resid            5.128047e-02    4.867973e-03     5.290914e-01
## Pennyscale.resid                      NA    1.597150e-02     3.749662e-03
## Jumpscale.resid             1.597150e-02              NA     3.269233e-03
## Standscale.resid            3.749662e-03    3.269233e-03               NA
## Hopscale.resid              3.833730e-05    2.888831e-03     6.894252e-05
## Catchscale.resid            3.458808e-03    1.452361e-04     2.296339e-02
## Dribblescale.resid          5.553637e-06    1.633662e-06     9.851106e-04
## Pushupscale.resid           2.533486e-01    1.599783e-01     2.524494e-02
## Situpscale.resid            5.296455e-02    6.874102e-02     1.122444e-03
##                         Hopscale.resid Catchscale.resid Dribblescale.resid
## Age_Acc                   7.756499e-01     7.428543e-04       7.150921e-01
## IQ                        5.074645e-02     2.835456e-01       4.338087e-03
## Daily_Live_Skills_standard 3.554695e-01     3.148049e-02       2.186525e-02
## Drawscale.resid           4.557298e-02     3.763799e-05       8.404775e-04
## Foldscale.resid           4.464380e-01     6.839501e-03       5.871102e-02
## Shapescale.resid          2.665852e-01     3.962232e-02       1.243981e-02
## Pennyscale.resid          3.833730e-05     3.458808e-03       5.553637e-06
## Jumpscale.resid           2.888831e-03     1.452361e-04       1.633662e-06
## Standscale.resid          6.894252e-05     2.296339e-02       9.851106e-04
## Hopscale.resid                      NA     1.858103e-02       8.884489e-04
## Catchscale.resid          1.858103e-02               NA       8.655040e-03
## Dribblescale.resid        8.884489e-04     8.655040e-03                 NA
## Pushupscale.resid         4.165350e-02     1.688234e-01       5.857036e-02
## Situpscale.resid          2.459877e-04     9.832721e-02       5.175622e-01
##                         Pushupscale.resid Situpscale.resid
## Age_Acc                         0.47648256     0.6175846283
## IQ                              0.15567356     0.2447792974
## Daily_Live_Skills_standard      0.85433325     0.5217720229
## Drawscale.resid                 0.28314346     0.2597900610
## Foldscale.resid                 0.73190166     0.9093584212
## Shapescale.resid                0.93840932     0.7559967988
## Pennyscale.resid                0.25334860     0.0529645457
## Jumpscale.resid                 0.15997833     0.0687410176
```

```
## Standscale.resid                 0.02524494     0.0011224438
## Hopscale.resid                   0.04165350     0.0002459877
## Catchscale.resid                 0.16882337     0.0983272110
## Dribblescale.resid               0.05857036     0.5175621810
## Pushupscale.resid                        NA     0.0201730638
## Situpscale.resid                 0.02017306               NA
```

```
res3$r
```

```
##                                Age_Acc         IQ Daily_Live_Skills_standard
## Age_Acc                     1.00000000 0.04758843                -0.19392107
## IQ                          0.04758843 1.00000000                 0.37600321
## Daily_Live_Skills_standard -0.19392107 0.37600321                 1.00000000
## Drawscale.resid            -0.16240349 0.22780610                 0.40270738
## Foldscale.resid             0.02374041 0.30807698                 0.39387343
## Shapescale.resid           -0.13201508 0.32520609                 0.26350231
## Pennyscale.resid           -0.12230219 0.37769060                 0.28038530
## Jumpscale.resid            -0.20537288 0.35672161                 0.31452969
## Standscale.resid           -0.04370975 0.16818995                 0.27177287
## Hopscale.resid             -0.03063513 0.20775453                 0.09910684
## Catchscale.resid           -0.35107402 0.11492043                 0.22820811
## Dribblescale.resid         -0.03923238 0.29961762                 0.24280921
## Pushupscale.resid          -0.07643887 0.15176788                 0.01973788
## Situpscale.resid           -0.05364340 0.12457095                 0.06879722
##                            Drawscale.resid Foldscale.resid Shapescale.resid
## Age_Acc                         -0.1624035      0.02374041     -0.132015084
## IQ                               0.2278061      0.30807698      0.325206091
## Daily_Live_Skills_standard       0.4027074      0.39387343      0.263502311
## Drawscale.resid                  1.0000000      0.23754884      0.350624367
## Foldscale.resid                  0.2375488      1.00000000      0.155764009
## Shapescale.resid                 0.3506244      0.15576401      1.000000000
## Pennyscale.resid                 0.2343898      0.40446206      0.207287229
## Jumpscale.resid                  0.5203058      0.21117817      0.295925229
## Standscale.resid                 0.2847901      0.10896369      0.067594772
## Hopscale.resid                   0.2125052      0.08172490      0.119025021
## Catchscale.resid                 0.4222354      0.28474755      0.218557280
## Dribblescale.resid               0.3477458      0.20116701      0.263952434
## Pushupscale.resid                0.1150158     -0.03682345     -0.008307832
## Situpscale.resid                 0.1207189     -0.01224043      0.033401572
##                            Pennyscale.resid Jumpscale.resid Standscale.resid
## Age_Acc                          -0.1223022      -0.2053729      -0.04370975
## IQ                                0.3776906       0.3567216       0.16818995
## Daily_Live_Skills_standard        0.2803853       0.3145297       0.27177287
## Drawscale.resid                   0.2343898       0.5203058       0.28479011
## Foldscale.resid                   0.4044621       0.2111782       0.10896369
## Shapescale.resid                  0.2072872       0.2959252       0.06759477
## Pennyscale.resid                  1.0000000       0.2547849       0.30422200
## Jumpscale.resid                   0.2547849       1.0000000       0.30848786
## Standscale.resid                  0.3042220       0.3084879       1.00000000
## Hopscale.resid                    0.4218420       0.3122843       0.40903984
## Catchscale.resid                  0.3067416       0.3920003       0.24089207
## Dribblescale.resid                0.4606742       0.4830204       0.34341164
## Pushupscale.resid                 0.1223527       0.1502220       0.23714675
## Situpscale.resid                  0.2058393       0.1938381       0.33980252
##                            Hopscale.resid Catchscale.resid Dribblescale.resid
## Age_Acc                       -0.03063513       -0.3510740        -0.03923238
## IQ                             0.20775453        0.1149204         0.29961762
## Daily_Live_Skills_standard     0.09910684        0.2282081         0.24280921
## Drawscale.resid                0.21250516        0.4222354         0.34774578
## Foldscale.resid                0.08172490        0.2847476         0.20116701
## Shapescale.resid               0.11902502        0.2185573         0.26395243
## Pennyscale.resid               0.42184202        0.3067416         0.46067416
## Jumpscale.resid                0.31228427        0.3920003         0.48302044
## Standscale.resid               0.40903984        0.2408921         0.34341164
## Hopscale.resid                 1.00000000        0.2490807         0.34623766
## Catchscale.resid               0.24908069        1.0000000         0.27674498
## Dribblescale.resid             0.34623766        0.2767450         1.00000000
## Pushupscale.resid              0.21641130        0.1471399         0.20127681
## Situpscale.resid               0.37936670        0.1763364         0.06949268
##                            Pushupscale.resid Situpscale.resid
## Age_Acc                         -0.076438868      -0.05364340
## IQ                               0.151767875       0.12457095
## Daily_Live_Skills_standard       0.019737885       0.06879722
## Drawscale.resid                  0.115015811       0.12071893
## Foldscale.resid                 -0.036823446      -0.01224043
## Shapescale.resid                -0.008307832       0.03340157
## Pennyscale.resid                 0.122352741       0.20583929
## Jumpscale.resid                  0.150221954       0.19383807
```

```
## Standscale.resid                   0.237146748       0.33980252
## Hopscale.resid                     0.216411304       0.37936670
## Catchscale.resid                   0.147139939       0.17633640
## Dribblescale.resid                 0.201276813       0.06949268
## Pushupscale.resid                  1.000000000       0.24593122
## Situpscale.resid                   0.245931222       1.00000000
```

```
Aim1_rvalue<-c(.40, .39, .26, .28, .31, .27, .10, .23, .24, .02, .07)
Aim1_pvalue<-c(9.136716e-05, 1.340769e-04 , 1.259584e-02, 7.782424e-03  ,  2.682999e-03, 9.985196e-03, 3.554695e-01, 3.148049e-02,  2.186525e-02,   8.543332e-01, 5.217720e-01)
Variable <- c("Draw", "Fold", "Shape", "Penny", "Jump", "Stand", "Hop", "Catch", "Dribble", "Pushup", "Situp")

Aim1_padj <- p.adjust(Aim1_pvalue, method="fdr")

cbind(Variable, Aim1_rvalue, Aim1_pvalue, Aim1_padj)
```

```
##       Variable  Aim1_rvalue Aim1_pvalue    Aim1_padj
##  [1,] "Draw"    "0.4"       "9.136716e-05" "0.00073742295"
##  [2,] "Fold"    "0.39"      "0.0001340769" "0.00073742295"
##  [3,] "Shape"   "0.26"      "0.01259584"   "0.0230923733333333"
##  [4,] "Penny"   "0.28"      "0.007782424"  "0.021401666"
##  [5,] "Jump"    "0.31"      "0.002682999"  "0.009837663"
##  [6,] "Stand"   "0.27"      "0.009985196"  "0.0219674312"
##  [7,] "Hop"     "0.1"       "0.3554695"    "0.434462722222222"
##  [8,] "Catch"   "0.23"      "0.03148049"   "0.04328567375"
##  [9,] "Dribble" "0.24"      "0.02186525"   "0.0343596785714286"
## [10,] "Pushup"  "0.02"      "0.8543332"    "0.8543332"
## [11,] "Situp"   "0.07"      "0.521772"     "0.5739492"
```

```
corrplot(res3$r, method="number", number.cex=.5)
```

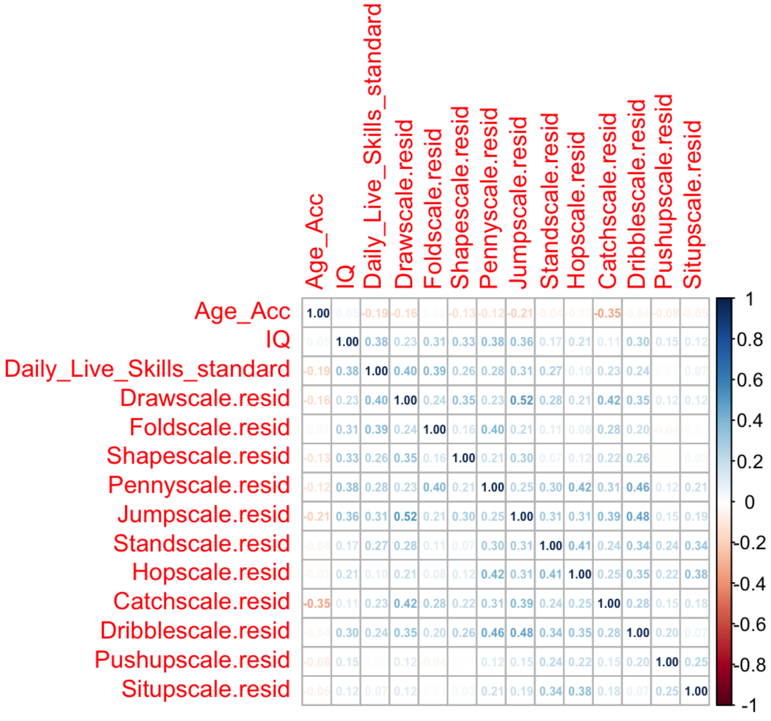

```
#This spreadsheet was revised in Excel based on the csv written earlier in this chunk. Rankings were performed fo
r each BOT-2 item and DLS.
Spearman<-read.csv("MotorDLS_1.9.24_v2.csv")

Drawingage3 <- ggplot(Spearman, aes(x=Draw_spearman, y=DLS_Spearman))+
  geom_point(size=.75, colour="#009E73")+
    geom_smooth(method="lm", alpha=.2, colour="gray")+
  labs(x="Drawing a Line \n Through a Crooked Path", y="Vineland DLS Standard Score")+
 theme_bw()+
  theme(axis.title = element_text(face="bold", size=8))+
 theme(legend.position = "none")

Foldingage3 <- ggplot(Spearman, aes(x=Fold_spearman, y=DLS_Spearman))+
  geom_point(size=.75, colour="#009E73")+
    geom_smooth(method="lm", alpha=.2, colour="gray")+
    labs(x="Folding Paper", y="Vineland DLS Standard Score")+
 theme_bw()+
  theme(axis.title = element_text(face="bold", size=8))+
 theme(legend.position = "none")

Shapeage3 <- ggplot(Spearman, aes(x=Shape_spearman, y=DLS_Spearman))+
  geom_point(size=.75, colour="#009E73")+
    geom_smooth(method="lm", alpha=.2, colour="gray")+
    labs(x="Copying a Square/Star", y="Vineland DLS Standard Score")+
 theme_bw()+
  theme(axis.title = element_text(face="bold", size=8))+
 theme(legend.position = "none")

Pennyage3 <- ggplot(Spearman, aes(x=Penny_spearman, y=DLS_Spearman))+
  geom_point(size=.75, colour="#009E73")+
    geom_smooth(method="lm", alpha=.2, colour="gray")+
    labs(x="Transferring Pennies", y="Vineland DLS Standard Score")+
 theme_bw()+
  theme(axis.title = element_text(face="bold", size=8))+
 theme(legend.position = "none")

Jumpage3 <- ggplot(Spearman, aes(x=Jump_spearman, y=DLS_Spearman))+
  geom_point(size=.75, colour="#009E73")+
    geom_smooth(method="lm", alpha=.2, colour="gray")+
    labs(x="Jumping in Place \n Same Sides Synchronized", y="Vineland DLS Standard Score")+
 theme_bw()+
  theme(axis.title = element_text(face="bold", size=8))+
 theme(legend.position = "none")

Standage3 <- ggplot(Spearman, aes(x=Stand_spearman, y=DLS_Spearman))+
  geom_point(size=.75, colour="#009E73")+
    geom_smooth(method="lm", alpha=.2, colour="gray")+
    labs(x="Standing on One Leg \n On a Balance Beam \n Eyes Open", y="Vineland DLS Standard Score")+
 theme_bw()+
  theme(axis.title = element_text(face="bold", size=8))+
 theme(legend.position = "none")

Hopage3 <- ggplot(Spearman, aes(x=Hop_spearman, y=DLS_Spearman))+
  geom_point(size=.75, colour="#009E73")+
    geom_smooth(method="lm", alpha=.2, colour="gray")+
    labs(x="One-Legged Stationary Hop", y="Vineland DLS Standard Score")+
 theme_bw()+
  theme(axis.title = element_text(face="bold", size=8))+
 theme(legend.position = "none")

Catchage3 <- ggplot(Spearman, aes(x=Catch_spearman, y=DLS_Spearman))+
  geom_point(size=.75, colour="#009E73")+
    geom_smooth(method="lm", alpha=.2, colour="gray")+
    labs(x="Dropping and Catching a Ball \n Both Hands", y="Vineland DLS Standard Score")+
 theme_bw()+
  theme(axis.title = element_text(face="bold", size=8))+
 theme(legend.position = "none")

Dribbleage3 <- ggplot(Spearman, aes(x=Dribble_spearman, y=DLS_Spearman))+
```

```
  geom_point(size=.75, colour="#009E73")+
    geom_smooth(method="lm", alpha=.2, colour="gray")+
    labs(x="Dribbling a Ball \n Alternating Hands", y="Vineland DLS Standard Score")+
 theme_bw()+
  theme(axis.title = element_text(face="bold", size=8))+
 theme(legend.position = "none")

Pushupage3 <- ggplot(Spearman, aes(x=Pushup_spearman, y=DLS_Spearman))+
  geom_point(size=.75, colour="#009E73")+
    geom_smooth(method="lm", alpha=.2, colour="gray")+
    labs(x="Push-ups", y="Vineland DLS Standard Score")+
 theme_bw()+
  theme(axis.title = element_text(face="bold", size=8))+
 theme(legend.position = "none")

Situpage3 <- ggplot(Spearman, aes(x=Situp_spearman, y=DLS_Spearman))+
  geom_point(size=.75, colour="#009E73")+
    geom_smooth(method="lm", alpha=.2, colour="gray")+
    labs(x="Sit-ups", y="Vineland DLS Standard Score")+
 theme_bw()+
  theme(axis.title = element_text(face="bold", size=8))+
 theme(legend.position = "none")

Drawingage3 + Foldingage3 + Shapeage3 + Pennyage3 + Jumpage3 + Standage3 + Hopage3 + Catchage3 + Dribbleage3 + Pu
shupage3 + Situpage3
```

```
## `geom_smooth()` using formula = 'y ~ x'
## `geom_smooth()` using formula = 'y ~ x'
## `geom_smooth()` using formula = 'y ~ x'
## `geom_smooth()` using formula = 'y ~ x'
## `geom_smooth()` using formula = 'y ~ x'
## `geom_smooth()` using formula = 'y ~ x'
## `geom_smooth()` using formula = 'y ~ x'
## `geom_smooth()` using formula = 'y ~ x'
## `geom_smooth()` using formula = 'y ~ x'
## `geom_smooth()` using formula = 'y ~ x'
## `geom_smooth()` using formula = 'y ~ x'
```

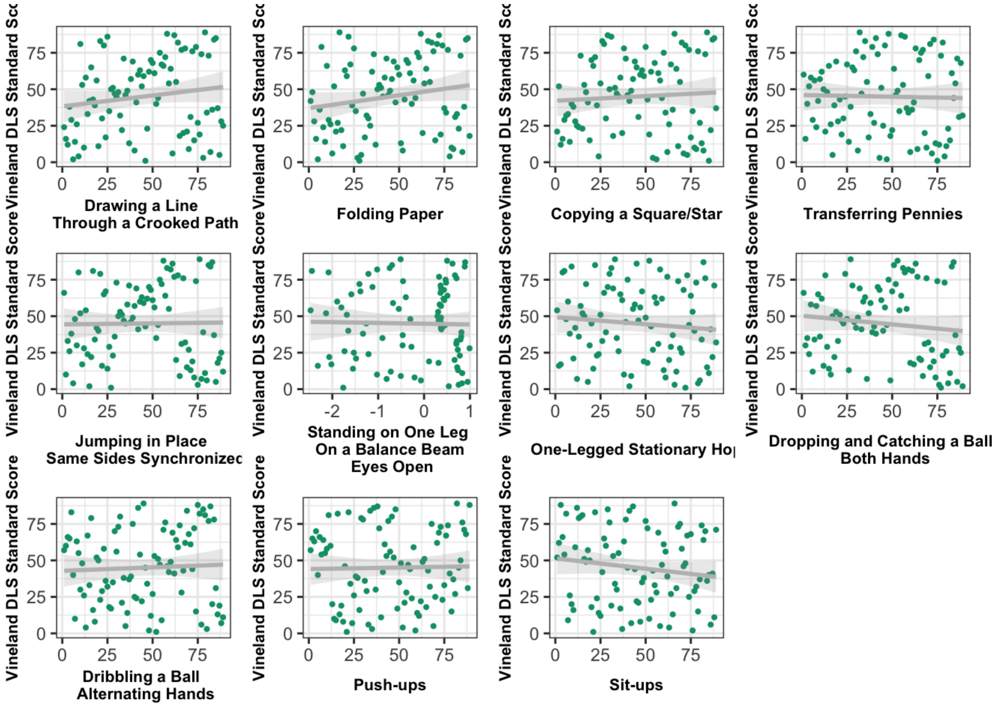


```
ggsave("SERP_fig1.png", height=6.5, width=8)
```

```
## `geom_smooth()` using formula = 'y ~ x'
## `geom_smooth()` using formula = 'y ~ x'
## `geom_smooth()` using formula = 'y ~ x'
## `geom_smooth()` using formula = 'y ~ x'
## `geom_smooth()` using formula = 'y ~ x'
## `geom_smooth()` using formula = 'y ~ x'
## `geom_smooth()` using formula = 'y ~ x'
## `geom_smooth()` using formula = 'y ~ x'
## `geom_smooth()` using formula = 'y ~ x'
## `geom_smooth()` using formula = 'y ~ x'
## `geom_smooth()` using formula = 'y ~ x'
```

```
DrawIQ <- lm(Daily_Live_Skills_standard~Drawscale.resid*IQ, data=d11sub)
FoldIQ <- lm(Daily_Live_Skills_standard~Foldscale.resid*IQ, data=d11sub)
ShapeIQ <- lm(Daily_Live_Skills_standard~Shapescale.resid*IQ, data=d11sub)
PennyIQ <- lm(Daily_Live_Skills_standard~Pennyscale.resid*IQ, data=d11sub)
JumpIQ <- lm(Daily_Live_Skills_standard~Jumpscale.resid*IQ, data=d11sub)
StandIQ <- lm(Daily_Live_Skills_standard~Standscale.resid*IQ, data=d11sub)
HopIQ <- lm(Daily_Live_Skills_standard~Hopscale.resid*IQ, data=d11sub)
CatchIQ <- lm(Daily_Live_Skills_standard~Catchscale.resid*IQ, data=d11sub)
DribbleIQ <- lm(Daily_Live_Skills_standard~Dribblescale.resid*IQ, data=d11sub)
PushupIQ <- lm(Daily_Live_Skills_standard~Pushupscale.resid*IQ, data=d11sub)
SitupIQ <- lm(Daily_Live_Skills_standard~Situpscale.resid*IQ, data=d11sub)

summary(DrawIQ)
```

```
##
## Call:
## lm(formula = Daily_Live_Skills_standard ~ Drawscale.resid * IQ,
##     data = d11sub)
##
## Residuals:
##     Min      1Q  Median      3Q     Max
## -36.079  -9.981  -0.793  10.315  27.291
##
## Coefficients:
##                     Estimate Std. Error t value Pr(>|t|)
## (Intercept)         57.35525   11.08479   5.174 1.51e-06 ***
## Drawscale.resid      2.37048    8.91043   0.266  0.79086
## IQ                   0.30945    0.10507   2.945  0.00416 **
## Drawscale.resid:IQ  0.02098    0.09249   0.227  0.82108
## ---
## Signif. codes:  0 '***' 0.001 '**' 0.01 '*' 0.05 '.' 0.1 ' ' 1
##
## Residual standard error: 14.67 on 85 degrees of freedom
## Multiple R-squared:  0.2007, Adjusted R-squared:  0.1725
## F-statistic: 7.114 on 3 and 85 DF,  p-value: 0.0002546
```

```
summary(FoldIQ)
```

```
##
## Call:
## lm(formula = Daily_Live_Skills_standard ~ Foldscale.resid * IQ,
##     data = d11sub)
##
## Residuals:
##     Min      1Q  Median      3Q     Max
## -30.913 -11.004  -0.022  10.103  32.319
##
## Coefficients:
##                      Estimate Std. Error t value Pr(>|t|)
## (Intercept)        54.95445   10.98982   5.000 3.04e-06 ***
## Foldscale.resid    -9.50715    9.36921  -1.015  0.31312
## IQ                  0.33398    0.10328   3.234  0.00174 **
## Foldscale.resid:IQ  0.13961    0.09619   1.451  0.15034
## ---
## Signif. codes:  0 '***' 0.001 '**' 0.01 '*' 0.05 '.' 0.1 ' ' 1
##
## Residual standard error: 14.52 on 85 degrees of freedom
## Multiple R-squared:  0.2171, Adjusted R-squared:  0.1894
## F-statistic: 7.855 on 3 and 85 DF,  p-value: 0.0001094
```

```
summary(ShapeIQ)
```

```
##
## Call:
## lm(formula = Daily_Live_Skills_standard ~ Shapescale.resid *
##     IQ, data = d11sub)
##
## Residuals:
##     Min      1Q  Median      3Q     Max
## -36.109 -10.871  -0.522  10.052  33.322
##
## Coefficients:
##                       Estimate Std. Error t value Pr(>|t|)
## (Intercept)         57.32901   11.86642   4.831 5.95e-06 ***
## Shapescale.resid     4.95687    9.56237   0.518  0.60555
## IQ                   0.31025    0.11155   2.781  0.00667 **
## Shapescale.resid:IQ -0.02597    0.09845  -0.264  0.79255
## ---
## Signif. codes:  0 '***' 0.001 '**' 0.01 '*' 0.05 '.' 0.1 ' ' 1
##
## Residual standard error: 14.96 on 85 degrees of freedom
## Multiple R-squared:  0.1695, Adjusted R-squared:  0.1402
## F-statistic: 5.785 on 3 and 85 DF,  p-value: 0.001198
```

```
summary(PennyIQ)
```

```
##
## Call:
## lm(formula = Daily_Live_Skills_standard ~ Pennyscale.resid *
##     IQ, data = d11sub)
##
## Residuals:
##     Min      1Q  Median      3Q     Max
## -33.306 -10.213   0.382   8.329  31.479
##
## Coefficients:
##                      Estimate Std. Error t value Pr(>|t|)
## (Intercept)         57.654241  11.978835   4.813 6.39e-06 ***
## Pennyscale.resid     3.885437  10.136377   0.383  0.70244
## IQ                   0.308864   0.111899   2.760  0.00707 **
## Pennyscale.resid:IQ -0.009762   0.099929  -0.098  0.92241
## ---
## Signif. codes:  0 '***' 0.001 '**' 0.01 '*' 0.05 '.' 0.1 ' ' 1
##
## Residual standard error: 14.92 on 85 degrees of freedom
## Multiple R-squared:  0.1738, Adjusted R-squared:  0.1446
## F-statistic: 5.958 on 3 and 85 DF,  p-value: 0.0009765
```

```
summary(JumpIQ)
```

```
##
## Call:
## lm(formula = Daily_Live_Skills_standard ~ Jumpscale.resid * IQ,
##     data = d11sub)
##
## Residuals:
##     Min      1Q  Median      3Q     Max
## -35.026 -10.642  -0.241  10.499  32.666
##
## Coefficients:
##                    Estimate Std. Error t value Pr(>|t|)
## (Intercept)        54.50464   12.65100   4.308  4.4e-05 ***
## Jumpscale.resid     4.85495    8.72362   0.557  0.57931
## IQ                  0.33577    0.11799   2.846  0.00555 **
## Jumpscale.resid:IQ -0.04274    0.08571  -0.499  0.61928
## ---
## Signif. codes:  0 '***' 0.001 '**' 0.01 '*' 0.05 '.' 0.1 ' ' 1
##
## Residual standard error: 15.09 on 85 degrees of freedom
## Multiple R-squared:  0.1541, Adjusted R-squared:  0.1242
## F-statistic: 5.161 on 3 and 85 DF,  p-value: 0.002517
```

```
summary(StandIQ)
```

```
##
## Call:
## lm(formula = Daily_Live_Skills_standard ~ Standscale.resid *
##     IQ, data = d11sub)
##
## Residuals:
##     Min      1Q  Median      3Q     Max
## -33.360 -10.323   1.391  10.871  30.108
##
## Coefficients:
##                       Estimate Std. Error t value Pr(>|t|)
## (Intercept)         61.24737   11.17898   5.479 4.29e-07 ***
## Standscale.resid    17.57299    9.89471   1.776   0.0793 .
## IQ                   0.27709    0.10578   2.619   0.0104 *
## Standscale.resid:IQ -0.15022    0.09794  -1.534   0.1288
## ---
## Signif. codes:  0 '***' 0.001 '**' 0.01 '*' 0.05 '.' 0.1 ' ' 1
##
## Residual standard error: 14.7 on 85 degrees of freedom
## Multiple R-squared:  0.1978, Adjusted R-squared:  0.1695
## F-statistic: 6.985 on 3 and 85 DF,  p-value: 0.0002951
```

```
summary(HopIQ)
```

```
##
## Call:
## lm(formula = Daily_Live_Skills_standard ~ Hopscale.resid * IQ,
##     data = d11sub)
##
## Residuals:
##     Min      1Q  Median      3Q     Max
## -35.375 -11.553  -0.433  11.100  30.234
##
## Coefficients:
##                     Estimate Std. Error t value Pr(>|t|)
## (Intercept)       54.48906   11.22013   4.856 5.39e-06 ***
## Hopscale.resid     9.87689    9.45962   1.044  0.29939
## IQ                 0.33548    0.10614   3.161  0.00218 **
## Hopscale.resid:IQ -0.09553    0.09230  -1.035  0.30362
## ---
## Signif. codes:  0 '***' 0.001 '**' 0.01 '*' 0.05 '.' 0.1 ' ' 1
##
## Residual standard error: 15.03 on 85 degrees of freedom
## Multiple R-squared:  0.161,  Adjusted R-squared:  0.1314
## F-statistic: 5.439 on 3 and 85 DF,  p-value: 0.001807
```

```
summary(CatchIQ)
```

```
##
## Call:
## lm(formula = Daily_Live_Skills_standard ~ Catchscale.resid *
##     IQ, data = d11sub)
##
## Residuals:
##     Min      1Q  Median      3Q     Max
## -35.170 -10.989  -0.578  11.011  29.751
##
## Coefficients:
##                       Estimate Std. Error t value Pr(>|t|)
## (Intercept)         50.20312   10.92619   4.595 1.49e-05 ***
## Catchscale.resid    -0.74701    8.47272  -0.088 0.929952
## IQ                   0.37454    0.10447   3.585 0.000561 ***
## Catchscale.resid:IQ  0.01934    0.08643   0.224 0.823429
## ---
## Signif. codes:  0 '***' 0.001 '**' 0.01 '*' 0.05 '.' 0.1 ' ' 1
##
## Residual standard error: 15.09 on 85 degrees of freedom
## Multiple R-squared:  0.1548, Adjusted R-squared:  0.125
## F-statistic:  5.19 on 3 and 85 DF,  p-value: 0.00243
```

```
summary(DribbleIQ)
```

```
##
## Call:
## lm(formula = Daily_Live_Skills_standard ~ Dribblescale.resid *
##     IQ, data = d11sub)
##
## Residuals:
##     Min      1Q  Median      3Q     Max
## -33.166 -12.201   0.721   9.536  30.879
##
## Coefficients:
##                         Estimate Std. Error t value Pr(>|t|)
## (Intercept)            54.6678    11.2633   4.854 5.44e-06 ***
## Dribblescale.resid      5.7334    13.5740   0.422  0.67381
## IQ                      0.3350     0.1059   3.163  0.00217 **
## Dribblescale.resid:IQ  -0.0345     0.1313  -0.263  0.79338
## ---
## Signif. codes:  0 '***' 0.001 '**' 0.01 '*' 0.05 '.' 0.1 ' ' 1
##
## Residual standard error: 15.02 on 85 degrees of freedom
## Multiple R-squared:  0.1623, Adjusted R-squared:  0.1327
## F-statistic: 5.488 on 3 and 85 DF,  p-value: 0.001703
```

```
summary(PushupIQ)
```

```
##
## Call:
## lm(formula = Daily_Live_Skills_standard ~ Pushupscale.resid *
##     IQ, data = d11sub)
##
## Residuals:
##    Min     1Q Median     3Q    Max
## -35.72 -10.34  -0.63  10.62  30.34
##
## Coefficients:
##                      Estimate Std. Error t value Pr(>|t|)
## (Intercept)          53.55857   14.16951   3.780 0.000291 ***
## Pushupscale.resid     5.73309   16.38755   0.350 0.727321
## IQ                    0.34503    0.13078   2.638 0.009908 **
## Pushupscale.resid:IQ -0.04794    0.15006  -0.320 0.750125
## ---
## Signif. codes:  0 '***' 0.001 '**' 0.01 '*' 0.05 '.' 0.1 ' ' 1
##
## Residual standard error: 15.11 on 85 degrees of freedom
## Multiple R-squared:  0.1519, Adjusted R-squared:  0.122
## F-statistic: 5.075 on 3 and 85 DF,  p-value: 0.002789
```

```
summary(SitupIQ)
```

```
##
## Call:
## lm(formula = Daily_Live_Skills_standard ~ Situpscale.resid *
##     IQ, data = d11sub)
##
## Residuals:
##    Min     1Q Median     3Q    Max
## -34.49 -10.63  -0.90  11.23  31.32
##
## Coefficients:
##                     Estimate Std. Error t value Pr(>|t|)
## (Intercept)         47.45091   12.21691   3.884 0.000203 ***
## Situpscale.resid    -3.60033   12.26860  -0.293 0.769887
## IQ                   0.39684    0.11468   3.460 0.000846 ***
## Situpscale.resid:IQ  0.02879    0.11414   0.252 0.801497
## ---
## Signif. codes:  0 '***' 0.001 '**' 0.01 '*' 0.05 '.' 0.1 ' ' 1
##
## Residual standard error: 15.11 on 85 degrees of freedom
## Multiple R-squared:  0.1518, Adjusted R-squared:  0.1219
## F-statistic: 5.071 on 3 and 85 DF,  p-value: 0.002802
```

```
DrawIQ2 <- lm(Daily_Live_Skills_standard~Drawscale.resid + IQ, data=d11sub)
FoldIQ2 <- lm(Daily_Live_Skills_standard~Foldscale.resid + IQ, data=d11sub)
ShapeIQ2 <- lm(Daily_Live_Skills_standard~Shapescale.resid + IQ, data=d11sub)
PennyIQ2 <- lm(Daily_Live_Skills_standard~Pennyscale.resid + IQ, data=d11sub)
JumpIQ2 <- lm(Daily_Live_Skills_standard~Jumpscale.resid + IQ, data=d11sub)
StandIQ2 <- lm(Daily_Live_Skills_standard~Standscale.resid + IQ, data=d11sub)
HopIQ2 <- lm(Daily_Live_Skills_standard~Hopscale.resid + IQ, data=d11sub)
CatchIQ2 <- lm(Daily_Live_Skills_standard~Catchscale.resid + IQ, data=d11sub)
DribbleIQ2 <- lm(Daily_Live_Skills_standard~Dribblescale.resid + IQ, data=d11sub)
PushupIQ2 <- lm(Daily_Live_Skills_standard~Pushupscale.resid + IQ, data=d11sub)
SitupIQ2 <- lm(Daily_Live_Skills_standard~Situpscale.resid + IQ, data=d11sub)

summary(DrawIQ2)
```

```
##
## Call:
## lm(formula = Daily_Live_Skills_standard ~ Drawscale.resid + IQ,
##     data = d11sub)
##
## Residuals:
##     Min      1Q  Median      3Q     Max
## -36.114 -10.110  -0.517  10.439  27.255
##
## Coefficients:
##                 Estimate Std. Error t value Pr(>|t|)
## (Intercept)     58.18659   10.40364   5.593  2.6e-07 ***
## Drawscale.resid  4.34603    1.87569   2.317  0.02288 *
## IQ               0.30204    0.09931   3.041  0.00312 **
## ---
## Signif. codes:  0 '***' 0.001 '**' 0.01 '*' 0.05 '.' 0.1 ' ' 1
##
## Residual standard error: 14.59 on 86 degrees of freedom
## Multiple R-squared:  0.2002, Adjusted R-squared:  0.1816
## F-statistic: 10.76 on 2 and 86 DF,  p-value: 6.731e-05
```

```
t_to_d(2.32, 86)
```

```
## d    |       95% CI
## -------------------
## 0.50 | [0.07, 0.93]
```

```
t_to_d(3.04, 86)
```

```
## d    |       95% CI
## -------------------
## 0.66 | [0.22, 1.09]
```

```
summary(FoldIQ2)
```

```
##
## Call:
## lm(formula = Daily_Live_Skills_standard ~ Foldscale.resid + IQ,
##     data = d11sub)
##
## Residuals:
##     Min      1Q  Median      3Q     Max
## -32.446 -11.516  -0.113  10.201  36.870
##
## Coefficients:
##                 Estimate Std. Error t value Pr(>|t|)
## (Intercept)      59.3041    10.6411   5.573 2.82e-07 ***
## Foldscale.resid   3.8647     1.7149   2.254  0.02676 *
## IQ                0.2964     0.1006   2.946  0.00415 **
## ---
## Signif. codes:  0 '***' 0.001 '**' 0.01 '*' 0.05 '.' 0.1 ' ' 1
##
## Residual standard error: 14.61 on 86 degrees of freedom
## Multiple R-squared:  0.1977, Adjusted R-squared:  0.179
## F-statistic: 10.59 on 2 and 86 DF,  p-value: 7.717e-05
```

```
t_to_d(2.25, 86)
```

```
## d    |       95% CI
## -------------------
## 0.49 | [0.06, 0.91]
```

```
t_to_d(2.95, 86)
```

```
## d    |       95% CI
## -------------------
## 0.64 | [0.20, 1.07]
```

```
summary(ShapeIQ2)
```

```
##
## Call:
## lm(formula = Daily_Live_Skills_standard ~ Shapescale.resid +
##     IQ, data = d11sub)
##
## Residuals:
##     Min      1Q  Median      3Q     Max
## -36.054 -11.306  -0.734  11.344  32.945
##
## Coefficients:
##                  Estimate Std. Error t value Pr(>|t|)
## (Intercept)       56.1629    10.9528   5.128 1.79e-06 ***
## Shapescale.resid   2.4790     1.7873   1.387  0.16901
## IQ                 0.3204     0.1041   3.079  0.00279 **
## ---
## Signif. codes:  0 '***' 0.001 '**' 0.01 '*' 0.05 '.' 0.1 ' ' 1
##
## Residual standard error: 14.87 on 86 degrees of freedom
## Multiple R-squared:  0.1689, Adjusted R-squared:  0.1495
## F-statistic: 8.737 on 2 and 86 DF,  p-value: 0.0003514
```

```
t_to_d(1.39, 86)
```

```
## d    |        95% CI
## --------------------
## 0.30 | [-0.13, 0.72]
```

```
t_to_d(3.08, 86)
```

```
## d    |       95% CI
## -------------------
## 0.66 | [0.23, 1.10]
```

```
summary(PennyIQ2)
```

```
##
## Call:
## lm(formula = Daily_Live_Skills_standard ~ Pennyscale.resid +
##     IQ, data = d11sub)
##
## Residuals:
##     Min      1Q  Median      3Q     Max
## -33.224 -10.192   0.381   8.377  31.407
##
## Coefficients:
##                  Estimate Std. Error t value Pr(>|t|)
## (Intercept)       57.2043    10.9941   5.203 1.31e-06 ***
## Pennyscale.resid   2.9123     1.8669   1.560  0.12244
## IQ                 0.3128     0.1040   3.008  0.00345 **
## ---
## Signif. codes:  0 '***' 0.001 '**' 0.01 '*' 0.05 '.' 0.1 ' ' 1
##
## Residual standard error: 14.83 on 86 degrees of freedom
## Multiple R-squared:  0.1737, Adjusted R-squared:  0.1544
## F-statistic: 9.037 on 2 and 86 DF,  p-value: 0.0002741
```

```
t_to_d(1.56, 86)
```

```
## d    |        95% CI
## ---------------------
## 0.34 | [-0.09, 0.76]
```

```
t_to_d(3.01, 86)
```

```
## d    |       95% CI
## --------------------
## 0.65 | [0.21, 1.08]
```

```
summary(JumpIQ2)
```

```
##
## Call:
## lm(formula = Daily_Live_Skills_standard ~ Jumpscale.resid + IQ,
##     data = d11sub)
##
## Residuals:
##     Min      1Q  Median      3Q     Max
## -34.634 -11.210  -0.283  10.699  31.493
##
## Coefficients:
##                 Estimate Std. Error t value Pr(>|t|)
## (Intercept)      51.5302    11.1080   4.639 1.24e-05 ***
## Jumpscale.resid   0.5755     1.5631   0.368 0.713652
## IQ                0.3622     0.1050   3.449 0.000874 ***
## ---
## Signif. codes:  0 '***' 0.001 '**' 0.01 '*' 0.05 '.' 0.1 ' ' 1
##
## Residual standard error: 15.03 on 86 degrees of freedom
## Multiple R-squared:  0.1516, Adjusted R-squared:  0.1319
## F-statistic: 7.684 on 2 and 86 DF,  p-value: 0.0008502
```

```
t_to_d(0.37, 86)
```

```
## d    |        95% CI
## ---------------------
## 0.08 | [-0.34, 0.50]
```

```
t_to_d(3.45, 86)
```

```
## d    |       95% CI
## --------------------
## 0.74 | [0.30, 1.18]
```

```
summary(StandIQ2)
```

```
##
## Call:
## lm(formula = Daily_Live_Skills_standard ~ Standscale.resid +
##     IQ, data = d11sub)
##
## Residuals:
##     Min      1Q  Median      3Q     Max
## -32.130  -9.916   0.383   9.976  30.772
##
## Coefficients:
##                  Estimate Std. Error t value Pr(>|t|)
## (Intercept)      54.16666   10.26102   5.279 9.62e-07 ***
## Standscale.resid  2.59190    1.59529   1.625 0.107883
## IQ                0.34191    0.09773   3.498 0.000744 ***
## ---
## Signif. codes:  0 '***' 0.001 '**' 0.01 '*' 0.05 '.' 0.1 ' ' 1
##
## Residual standard error: 14.81 on 86 degrees of freedom
## Multiple R-squared:  0.1756, Adjusted R-squared:  0.1564
## F-statistic: 9.158 on 2 and 86 DF,  p-value: 0.000248
```

```
t_to_d(1.63, 86)
```

```
## d    |        95% CI
## --------------------
## 0.35 | [-0.08, 0.78]
```

```
t_to_d(3.50, 86)
```

```
## d    |       95% CI
## -------------------
## 0.75 | [0.32, 1.19]
```

```
summary(HopIQ2)
```

```
##
## Call:
## lm(formula = Daily_Live_Skills_standard ~ Hopscale.resid + IQ,
##     data = d11sub)
##
## Residuals:
##     Min      1Q  Median      3Q     Max
## -35.078 -11.076  -0.732  10.953  30.523
##
## Coefficients:
##                Estimate Std. Error t value Pr(>|t|)
## (Intercept)    50.16747   10.41852   4.815 6.24e-06 ***
## Hopscale.resid  0.20881    1.49128   0.140 0.888973
## IQ              0.37403    0.09943   3.762 0.000307 ***
## ---
## Signif. codes:  0 '***' 0.001 '**' 0.01 '*' 0.05 '.' 0.1 ' ' 1
##
## Residual standard error: 15.04 on 86 degrees of freedom
## Multiple R-squared:  0.1505, Adjusted R-squared:  0.1307
## F-statistic: 7.616 on 2 and 86 DF,  p-value: 0.0009009
```

```
t_to_d(0.14, 86)
```

```
## d    |        95% CI
## --------------------
## 0.03 | [-0.39, 0.45]
```

```
t_to_d(3.76, 86)
```

```
## d    |       95% CI
## --------------------
## 0.81 | [0.37, 1.25]
```

```
summary(CatchIQ2)
```

```
##
## Call:
## lm(formula = Daily_Live_Skills_standard ~ Catchscale.resid +
##     IQ, data = d11sub)
##
## Residuals:
##    Min     1Q Median     3Q    Max
## -35.19 -11.06  -0.62  11.01  29.15
##
## Coefficients:
##                  Estimate Std. Error t value Pr(>|t|)
## (Intercept)      51.03666   10.21506   4.996 3.04e-06 ***
## Catchscale.resid  1.10913    1.72725   0.642 0.522492
## IQ                0.36671    0.09789   3.746 0.000324 ***
## ---
## Signif. codes:  0 '***' 0.001 '**' 0.01 '*' 0.05 '.' 0.1 ' ' 1
##
## Residual standard error: 15 on 86 degrees of freedom
## Multiple R-squared:  0.1543, Adjusted R-squared:  0.1347
## F-statistic: 7.847 on 2 and 86 DF,  p-value: 0.0007407
```

```
t_to_d(0.64, 86)
```

```
## d    |        95% CI
## ---------------------
## 0.14 | [-0.29, 0.56]
```

```
t_to_d(3.75, 86)
```

```
## d    |       95% CI
## --------------------
## 0.81 | [0.37, 1.25]
```

```
summary(DribbleIQ2)
```

```
##
## Call:
## lm(formula = Daily_Live_Skills_standard ~ Dribblescale.resid +
##     IQ, data = d11sub)
##
## Residuals:
##     Min      1Q  Median      3Q     Max
## -32.964 -12.052   0.757   9.436  30.570
##
## Coefficients:
##                    Estimate Std. Error t value Pr(>|t|)
## (Intercept)         53.7401    10.6376   5.052 2.43e-06 ***
## Dribblescale.resid   2.2081     2.0492   1.078  0.28424
## IQ                   0.3427     0.1013   3.383  0.00108 **
## ---
## Signif. codes:  0 '***' 0.001 '**' 0.01 '*' 0.05 '.' 0.1 ' ' 1
##
## Residual standard error: 14.94 on 86 degrees of freedom
## Multiple R-squared:  0.1616, Adjusted R-squared:  0.1421
## F-statistic: 8.288 on 2 and 86 DF,  p-value: 0.0005111
```

```
t_to_d(1.08, 86)
```

```
## d    |        95% CI
## ---------------------
## 0.23 | [-0.19, 0.66]
```

```
t_to_d(3.38, 86)
```

```
## d    |       95% CI
## -------------------
## 0.73 | [0.29, 1.16]
```

```
summary(PushupIQ2)
```

```
##
## Call:
## lm(formula = Daily_Live_Skills_standard ~ Pushupscale.resid +
##     IQ, data = d11sub)
##
## Residuals:
##     Min      1Q  Median      3Q     Max
## -35.442 -11.124  -0.379  10.661  30.654
##
## Coefficients:
##                   Estimate Std. Error t value Pr(>|t|)
## (Intercept)       50.53066   10.47890   4.822 6.07e-06 ***
## Pushupscale.resid  0.54344    2.16324   0.251 0.802246
## IQ                 0.37224    0.09874   3.770 0.000299 ***
## ---
## Signif. codes:  0 '***' 0.001 '**' 0.01 '*' 0.05 '.' 0.1 ' ' 1
##
## Residual standard error: 15.03 on 86 degrees of freedom
## Multiple R-squared:  0.1509, Adjusted R-squared:  0.1312
## F-statistic: 7.642 on 2 and 86 DF,  p-value: 0.0008816
```

```
t_to_d(.25, 86)
```

```
## d    |        95% CI
## ---------------------
## 0.05 | [-0.37, 0.48]
```

```
t_to_d(3.77, 86)
```

```
## d    |       95% CI
## -------------------
## 0.81 | [0.37, 1.25]
```

```
summary(SitupIQ2)
```

```
##
## Call:
## lm(formula = Daily_Live_Skills_standard ~ Situpscale.resid +
##     IQ, data = d11sub)
##
## Residuals:
##     Min      1Q  Median      3Q     Max
## -34.605 -10.795  -0.683  11.146  31.009
##
## Coefficients:
##                  Estimate Std. Error t value Pr(>|t|)
## (Intercept)      49.08214   10.30762   4.762  7.7e-06 ***
## Situpscale.resid -0.53927    1.77875  -0.303  0.76249
## IQ                0.38203    0.09796   3.900  0.00019 ***
## ---
## Signif. codes:  0 '***' 0.001 '**' 0.01 '*' 0.05 '.' 0.1 ' ' 1
##
## Residual standard error: 15.03 on 86 degrees of freedom
## Multiple R-squared:  0.1512, Adjusted R-squared:  0.1314
## F-statistic: 7.659 on 2 and 86 DF,  p-value: 0.000869
```

```
t_to_d(0.30, 86)
```

```
## d    |        95% CI
## --------------------
## 0.06 | [-0.36, 0.49]
```

```
t_to_d(3.90, 86)
```

```
## d    |       95% CI
## -------------------
## 0.84 | [0.40, 1.28]
```

```
MotorDLS_ASD_SEQfu <- subset(MotorDLS_ASD2, MotorDLS_ASD2$subject!="H058")
MotorDLS_wSEQ<-merge(d11,MotorDLS_ASD_SEQfu[,c(1, 206)],by="subject", all.x = TRUE, all.y=FALSE)
MotorDLS_wSEQ2<-subset(MotorDLS_wSEQ, is.na(MotorDLS_wSEQ$SEQ_totalpt)==FALSE)

DrawSEQ <- lm(Daily_Live_Skills_standard~Drawscale.resid*SEQ_totalpt, data=MotorDLS_wSEQ2)
FoldSEQ <- lm(Daily_Live_Skills_standard~Foldscale.resid*SEQ_totalpt, data=MotorDLS_wSEQ2)
ShapeSEQ <- lm(Daily_Live_Skills_standard~Shapescale.resid*SEQ_totalpt, data=MotorDLS_wSEQ2)
PennySEQ <- lm(Daily_Live_Skills_standard~Pennyscale.resid*SEQ_totalpt, data=MotorDLS_wSEQ2)
JumpSEQ <- lm(Daily_Live_Skills_standard~Jumpscale.resid*SEQ_totalpt, data=MotorDLS_wSEQ2)
StandSEQ <- lm(Daily_Live_Skills_standard~Standscale.resid*SEQ_totalpt, data=MotorDLS_wSEQ2)
HopSEQ <- lm(Daily_Live_Skills_standard~Hopscale.resid*SEQ_totalpt, data=MotorDLS_wSEQ2)
CatchSEQ <- lm(Daily_Live_Skills_standard~Catchscale.resid*SEQ_totalpt, data=MotorDLS_wSEQ2)
DribbleSEQ <- lm(Daily_Live_Skills_standard~Dribblescale.resid*SEQ_totalpt, data=MotorDLS_wSEQ2)
PushupSEQ <- lm(Daily_Live_Skills_standard~Pushupscale.resid*SEQ_totalpt, data=MotorDLS_wSEQ2)
SitupSEQ <- lm(Daily_Live_Skills_standard~Situpscale.resid*SEQ_totalpt, data=MotorDLS_wSEQ2)

summary(DrawSEQ)
```

```
##
## Call:
## lm(formula = Daily_Live_Skills_standard ~ Drawscale.resid * SEQ_totalpt,
##     data = MotorDLS_wSEQ2)
##
## Residuals:
##     Min      1Q  Median      3Q     Max
## -30.981  -8.588   1.393   9.793  34.725
##
## Coefficients:
##                              Estimate Std. Error t value Pr(>|t|)
## (Intercept)                 110.24841    8.06353  13.672  < 2e-16 ***
## Drawscale.resid              17.35012   10.18285   1.704  0.09219 .
## SEQ_totalpt                  -0.09674    0.03656  -2.646  0.00976 **
## Drawscale.resid:SEQ_totalpt  -0.05044    0.04065  -1.241  0.21815
## ---
## Signif. codes:  0 '***' 0.001 '**' 0.01 '*' 0.05 '.' 0.1 ' ' 1
##
## Residual standard error: 14.92 on 82 degrees of freedom
## Multiple R-squared:  0.1808, Adjusted R-squared:  0.1508
## F-statistic: 6.031 on 3 and 82 DF,  p-value: 0.0009182
```

```
summary(FoldSEQ)
```

```
##
## Call:
## lm(formula = Daily_Live_Skills_standard ~ Foldscale.resid * SEQ_totalpt,
##     data = MotorDLS_wSEQ2)
##
## Residuals:
##     Min      1Q  Median      3Q     Max
## -29.232  -9.751  -0.108  10.869  33.081
##
## Coefficients:
##                              Estimate Std. Error t value Pr(>|t|)
## (Intercept)                 107.35682    8.11547  13.229   <2e-16 ***
## Foldscale.resid              -1.30505    9.92573  -0.131   0.8957
## SEQ_totalpt                  -0.07876    0.03660  -2.152   0.0343 *
## Foldscale.resid:SEQ_totalpt   0.02481    0.04072   0.609   0.5440
## ---
## Signif. codes:  0 '***' 0.001 '**' 0.01 '*' 0.05 '.' 0.1 ' ' 1
##
## Residual standard error: 15.02 on 82 degrees of freedom
## Multiple R-squared:  0.1702, Adjusted R-squared:  0.1398
## F-statistic: 5.606 on 3 and 82 DF,  p-value: 0.001512
```

```
summary(ShapeSEQ)
```

```
##
## Call:
## lm(formula = Daily_Live_Skills_standard ~ Shapescale.resid *
##     SEQ_totalpt, data = MotorDLS_wSEQ2)
##
## Residuals:
##     Min      1Q  Median      3Q     Max
## -27.668 -10.176  -1.215  11.580  31.641
##
## Coefficients:
##                               Estimate Std. Error t value Pr(>|t|)
## (Intercept)                  108.67931    7.88830  13.777   <2e-16 ***
## Shapescale.resid              -1.26889    9.44741  -0.134   0.8935
## SEQ_totalpt                   -0.08681    0.03526  -2.462   0.0159 *
## Shapescale.resid:SEQ_totalpt   0.02249    0.03833   0.587   0.5590
## ---
## Signif. codes:  0 '***' 0.001 '**' 0.01 '*' 0.05 '.' 0.1 ' ' 1
##
## Residual standard error: 15.08 on 82 degrees of freedom
## Multiple R-squared:  0.1637, Adjusted R-squared:  0.1331
## F-statistic: 5.351 on 3 and 82 DF,  p-value: 0.002045
```

```
summary(PennySEQ)
```

```
##
## Call:
## lm(formula = Daily_Live_Skills_standard ~ Pennyscale.resid *
##     SEQ_totalpt, data = MotorDLS_wSEQ2)
##
## Residuals:
##     Min      1Q  Median      3Q     Max
## -28.513 -10.300  -0.129   9.465  33.960
##
## Coefficients:
##                               Estimate Std. Error t value Pr(>|t|)
## (Intercept)                  103.49371    8.54271  12.115   <2e-16 ***
## Pennyscale.resid              -8.63378    8.15583  -1.059    0.293
## SEQ_totalpt                   -0.06056    0.03894  -1.555    0.124
## Pennyscale.resid:SEQ_totalpt   0.06017    0.03731   1.613    0.111
## ---
## Signif. codes:  0 '***' 0.001 '**' 0.01 '*' 0.05 '.' 0.1 ' ' 1
##
## Residual standard error: 14.93 on 82 degrees of freedom
## Multiple R-squared:  0.1799, Adjusted R-squared:  0.1499
## F-statistic: 5.996 on 3 and 82 DF,  p-value: 0.0009568
```

```
summary(JumpSEQ)
```

```
##
## Call:
## lm(formula = Daily_Live_Skills_standard ~ Jumpscale.resid * SEQ_totalpt,
##     data = MotorDLS_wSEQ2)
##
## Residuals:
##     Min      1Q  Median      3Q     Max
## -32.443  -9.585  -0.941  11.230  34.410
##
## Coefficients:
##                              Estimate Std. Error t value Pr(>|t|)
## (Intercept)                 107.33172    8.47156  12.670   <2e-16 ***
## Jumpscale.resid              -6.65816    8.23343  -0.809   0.4210
## SEQ_totalpt                  -0.08138    0.03901  -2.086   0.0401 *
## Jumpscale.resid:SEQ_totalpt   0.03376    0.03488   0.968   0.3359
## ---
## Signif. codes:  0 '***' 0.001 '**' 0.01 '*' 0.05 '.' 0.1 ' ' 1
##
## Residual standard error: 15.53 on 82 degrees of freedom
## Multiple R-squared:  0.113,  Adjusted R-squared:  0.0806
## F-statistic: 3.484 on 3 and 82 DF,  p-value: 0.01947
```

```
summary(StandSEQ)
```

```
##
## Call:
## lm(formula = Daily_Live_Skills_standard ~ Standscale.resid *
##     SEQ_totalpt, data = MotorDLS_wSEQ2)
##
## Residuals:
##     Min      1Q  Median      3Q     Max
## -28.353 -10.365   0.972  10.713  38.024
##
## Coefficients:
##                                Estimate Std. Error t value Pr(>|t|)
## (Intercept)                  108.195139   8.575709  12.616   <2e-16 ***
## Standscale.resid               1.150342   7.547208   0.152   0.8792
## SEQ_totalpt                   -0.084356   0.039369  -2.143   0.0351 *
## Standscale.resid:SEQ_totalpt   0.008157   0.033198   0.246   0.8065
## ---
## Signif. codes:  0 '***' 0.001 '**' 0.01 '*' 0.05 '.' 0.1 ' ' 1
##
## Residual standard error: 15.37 on 82 degrees of freedom
## Multiple R-squared:  0.1308, Adjusted R-squared:  0.09896
## F-statistic: 4.112 on 3 and 82 DF,  p-value: 0.009052
```

```
summary(HopSEQ)
```

```
##
## Call:
## lm(formula = Daily_Live_Skills_standard ~ Hopscale.resid * SEQ_totalpt,
##     data = MotorDLS_wSEQ2)
##
## Residuals:
##     Min      1Q  Median      3Q     Max
## -29.726 -10.728  -0.109  11.504  36.331
##
## Coefficients:
##                             Estimate Std. Error t value Pr(>|t|)
## (Intercept)                108.32983    8.57963  12.626   <2e-16 ***
## Hopscale.resid              -6.67070    7.37924  -0.904   0.3687
## SEQ_totalpt                 -0.08798    0.03925  -2.242   0.0277 *
## Hopscale.resid:SEQ_totalpt   0.03026    0.03256   0.929   0.3555
## ---
## Signif. codes:  0 '***' 0.001 '**' 0.01 '*' 0.05 '.' 0.1 ' ' 1
##
## Residual standard error: 15.58 on 82 degrees of freedom
## Multiple R-squared:  0.1064, Adjusted R-squared:  0.07375
## F-statistic: 3.256 on 3 and 82 DF,  p-value: 0.02574
```

```
summary(CatchSEQ)
```

```
##
## Call:
## lm(formula = Daily_Live_Skills_standard ~ Catchscale.resid *
##     SEQ_totalpt, data = MotorDLS_wSEQ2)
##
## Residuals:
##     Min      1Q  Median      3Q     Max
## -29.502  -9.693  -0.283  12.031  36.219
##
## Coefficients:
##                                Estimate Std. Error t value Pr(>|t|)
## (Intercept)                  110.874612   8.045590  13.781  < 2e-16 ***
## Catchscale.resid               0.853108   9.189781   0.093  0.92626
## SEQ_totalpt                   -0.099912   0.035802  -2.791  0.00654 **
## Catchscale.resid:SEQ_totalpt   0.003981   0.037715   0.106  0.91620
## ---
## Signif. codes:  0 '***' 0.001 '**' 0.01 '*' 0.05 '.' 0.1 ' ' 1
##
## Residual standard error: 15.57 on 82 degrees of freedom
## Multiple R-squared:  0.1084, Adjusted R-squared:  0.07582
## F-statistic: 3.324 on 3 and 82 DF,  p-value: 0.02367
```

```
summary(DribbleSEQ)
```

```
##
## Call:
## lm(formula = Daily_Live_Skills_standard ~ Dribblescale.resid *
##     SEQ_totalpt, data = MotorDLS_wSEQ2)
##
## Residuals:
##     Min      1Q  Median      3Q     Max
## -27.625 -11.528  -0.042  10.917  37.757
##
## Coefficients:
##                                 Estimate Std. Error t value Pr(>|t|)
## (Intercept)                    105.96528    8.19391  12.932   <2e-16 ***
## Dribblescale.resid              -9.00294    9.77010  -0.921   0.3595
## SEQ_totalpt                     -0.07312    0.03745  -1.953   0.0543 .
## Dribblescale.resid:SEQ_totalpt   0.05897    0.04515   1.306   0.1952
## ---
## Signif. codes:  0 '***' 0.001 '**' 0.01 '*' 0.05 '.' 0.1 ' ' 1
##
## Residual standard error: 15.24 on 82 degrees of freedom
## Multiple R-squared:  0.1451, Adjusted R-squared:  0.1139
## F-statistic: 4.641 on 3 and 82 DF,  p-value: 0.004776
```

```
summary(PushupSEQ)
```

```
##
## Call:
## lm(formula = Daily_Live_Skills_standard ~ Pushupscale.resid *
##     SEQ_totalpt, data = MotorDLS_wSEQ2)
##
## Residuals:
##     Min      1Q  Median      3Q     Max
## -28.293 -10.350   0.512  11.536  35.323
##
## Coefficients:
##                                Estimate Std. Error t value Pr(>|t|)
## (Intercept)                   110.32002    8.87967  12.424   <2e-16 ***
## Pushupscale.resid              -3.48158   11.43699  -0.304   0.7616
## SEQ_totalpt                    -0.09410    0.03991  -2.358   0.0208 *
## Pushupscale.resid:SEQ_totalpt   0.02625    0.05401   0.486   0.6283
## ---
## Signif. codes:  0 '***' 0.001 '**' 0.01 '*' 0.05 '.' 0.1 ' ' 1
##
## Residual standard error: 15.57 on 82 degrees of freedom
## Multiple R-squared:  0.1082, Adjusted R-squared:  0.07559
## F-statistic: 3.317 on 3 and 82 DF,  p-value: 0.02389
```

```
summary(SitupSEQ)
```

```
##
## Call:
## lm(formula = Daily_Live_Skills_standard ~ Situpscale.resid *
##     SEQ_totalpt, data = MotorDLS_wSEQ2)
##
## Residuals:
##     Min      1Q  Median      3Q     Max
## -28.299  -9.487  -0.192  11.543  37.700
##
## Coefficients:
##                              Estimate Std. Error t value Pr(>|t|)
## (Intercept)                 110.30485    8.82643  12.497   <2e-16 ***
## Situpscale.resid             -2.44088    9.19186  -0.266   0.7913
## SEQ_totalpt                  -0.09845    0.03976  -2.476   0.0153 *
## Situpscale.resid:SEQ_totalpt   0.01132    0.04304   0.263   0.7932
## ---
## Signif. codes:  0 '***' 0.001 '**' 0.01 '*' 0.05 '.' 0.1 ' ' 1
##
## Residual standard error: 15.66 on 82 degrees of freedom
## Multiple R-squared:  0.09781,    Adjusted R-squared:  0.0648
## F-statistic: 2.963 on 3 and 82 DF,  p-value: 0.03688
```

```
DrawSEQ2 <- lm(Daily_Live_Skills_standard~Drawscale.resid +SEQ_totalpt, data=MotorDLS_wSEQ2)
FoldSEQ2 <- lm(Daily_Live_Skills_standard~Foldscale.resid + SEQ_totalpt, data=MotorDLS_wSEQ2)
ShapeSEQ2 <- lm(Daily_Live_Skills_standard~Shapescale.resid + SEQ_totalpt, data=MotorDLS_wSEQ2)
PennySEQ2 <- lm(Daily_Live_Skills_standard~Pennyscale.resid +SEQ_totalpt, data=MotorDLS_wSEQ2)
JumpSEQ2 <- lm(Daily_Live_Skills_standard~Jumpscale.resid + SEQ_totalpt, data=MotorDLS_wSEQ2)
StandSEQ2 <- lm(Daily_Live_Skills_standard~Standscale.resid + SEQ_totalpt, data=MotorDLS_wSEQ2)
HopSEQ2 <- lm(Daily_Live_Skills_standard~Hopscale.resid + SEQ_totalpt, data=MotorDLS_wSEQ2)
CatchSEQ2 <- lm(Daily_Live_Skills_standard~Catchscale.resid + SEQ_totalpt, data=MotorDLS_wSEQ2)
DribbleSEQ2 <- lm(Daily_Live_Skills_standard~Dribblescale.resid +SEQ_totalpt, data=MotorDLS_wSEQ2)
PushupSEQ2 <- lm(Daily_Live_Skills_standard~Pushupscale.resid + SEQ_totalpt, data=MotorDLS_wSEQ2)
SitupSEQ2 <- lm(Daily_Live_Skills_standard~Situpscale.resid + SEQ_totalpt, data=MotorDLS_wSEQ2)

summary(DrawSEQ2)
```

```
##
## Call:
## lm(formula = Daily_Live_Skills_standard ~ Drawscale.resid + SEQ_totalpt,
##     data = MotorDLS_wSEQ2)
##
## Residuals:
##     Min      1Q  Median      3Q     Max
## -32.795  -9.039   0.615  10.731  33.406
##
## Coefficients:
##                  Estimate Std. Error t value Pr(>|t|)
## (Intercept)     107.13083    7.68710  13.936   <2e-16 ***
## Drawscale.resid   4.93194    1.89177   2.607   0.0108 *
## SEQ_totalpt      -0.08099    0.03440  -2.354   0.0209 *
## ---
## Signif. codes:  0 '***' 0.001 '**' 0.01 '*' 0.05 '.' 0.1 ' ' 1
##
## Residual standard error: 14.97 on 83 degrees of freedom
## Multiple R-squared:  0.1654, Adjusted R-squared:  0.1453
## F-statistic: 8.223 on 2 and 83 DF,  p-value: 0.0005519
```

```
t_to_d(2.61, 83)
```

```
## d    |       95% CI
## -------------------
## 0.57 | [0.13, 1.01]
```

```
t_to_d(-2.35, 83)
```

```
## d     |         95% CI
## ----------------------
## -0.52 | [-0.95, -0.08]
```

```
summary(FoldSEQ2)
```

```
## 
## Call:
## lm(formula = Daily_Live_Skills_standard ~ Foldscale.resid + SEQ_totalpt, 
##     data = MotorDLS_wSEQ2)
## 
## Residuals:
##     Min      1Q  Median      3Q     Max 
## -29.807  -9.508   0.504  10.834  34.101 
## 
## Coefficients:
##                  Estimate Std. Error t value Pr(>|t|)    
## (Intercept)     109.11370    7.55714  14.438   <2e-16 ***
## Foldscale.resid   4.64525    1.76704   2.629   0.0102 *  
## SEQ_totalpt      -0.08703    0.03386  -2.570   0.0120 *  
## ---
## Signif. codes:  0 '***' 0.001 '**' 0.01 '*' 0.05 '.' 0.1 ' ' 1
## 
## Residual standard error: 14.96 on 83 degrees of freedom
## Multiple R-squared:  0.1664, Adjusted R-squared:  0.1463 
## F-statistic: 8.286 on 2 and 83 DF,  p-value: 0.0005236
```

```
t_to_d(2.63, 83)
```

```
## d    |       95% CI
## -------------------
## 0.58 | [0.14, 1.01]
```

```
t_to_d(2.57, 83)
```

```
## d    |       95% CI
## -------------------
## 0.56 | [0.12, 1.00]
```

```
summary(ShapeSEQ2)
```

```
## 
## Call:
## lm(formula = Daily_Live_Skills_standard ~ Shapescale.resid + 
##     SEQ_totalpt, data = MotorDLS_wSEQ2)
## 
## Residuals:
##     Min      1Q  Median      3Q     Max 
## -27.268 -10.933  -2.196  11.763  32.849 
## 
## Coefficients:
##                   Estimate Std. Error t value Pr(>|t|)    
## (Intercept)      109.94470    7.55776  14.547  < 2e-16 ***
## Shapescale.resid   4.18599    1.67526   2.499  0.01444 *  
## SEQ_totalpt       -0.09266    0.03368  -2.751  0.00729 ** 
## ---
## Signif. codes:  0 '***' 0.001 '**' 0.01 '*' 0.05 '.' 0.1 ' ' 1
## 
## Residual standard error: 15.02 on 83 degrees of freedom
## Multiple R-squared:  0.1602, Adjusted R-squared:   0.14 
## F-statistic: 7.917 on 2 and 83 DF,  p-value: 0.0007132
```

```
t_to_d(2.50, 83)
```

```
## d    |       95% CI
## -------------------
## 0.55 | [0.11, 0.99]
```

```
t_to_d(2.75, 83)
```

```
## d    |       95% CI
## -------------------
## 0.60 | [0.16, 1.04]
```

```
summary(PennySEQ2)
```

```
##
## Call:
## lm(formula = Daily_Live_Skills_standard ~ Pennyscale.resid +
##     SEQ_totalpt, data = MotorDLS_wSEQ2)
##
## Residuals:
##     Min      1Q  Median      3Q     Max
## -31.409  -9.609   0.005   9.733  36.587
##
## Coefficients:
##                   Estimate Std. Error t value Pr(>|t|)
## (Intercept)      110.05045    7.58510  14.509  < 2e-16 ***
## Pennyscale.resid   4.20667    1.78121   2.362  0.02053 *
## SEQ_totalpt       -0.09248    0.03385  -2.732  0.00769 **
## ---
## Signif. codes:  0 '***' 0.001 '**' 0.01 '*' 0.05 '.' 0.1 ' ' 1
##
## Residual standard error: 15.07 on 83 degrees of freedom
## Multiple R-squared:  0.1539, Adjusted R-squared:  0.1335
## F-statistic: 7.548 on 2 and 83 DF,  p-value: 0.0009733
```

```
t_to_d(2.36, 83)
```

```
## d    |       95% CI
## -------------------
## 0.52 | [0.08, 0.95]
```

```
t_to_d(2.73, 83)
```

```
## d    |       95% CI
## -------------------
## 0.60 | [0.16, 1.04]
```

```
summary(JumpSEQ2)
```

```
##
## Call:
## lm(formula = Daily_Live_Skills_standard ~ Jumpscale.resid + SEQ_totalpt,
##     data = MotorDLS_wSEQ2)
##
## Residuals:
##     Min      1Q  Median      3Q     Max
## -30.159 -10.394  -0.755  11.633  36.859
##
## Coefficients:
##                  Estimate Std. Error t value Pr(>|t|)
## (Intercept)     109.97226    8.01724  13.717   <2e-16 ***
## Jumpscale.resid   1.16350    1.57723   0.738   0.4628
## SEQ_totalpt      -0.09529    0.03625  -2.629   0.0102 *
## ---
## Signif. codes:  0 '***' 0.001 '**' 0.01 '*' 0.05 '.' 0.1 ' ' 1
##
## Residual standard error: 15.52 on 83 degrees of freedom
## Multiple R-squared:  0.1029, Adjusted R-squared:  0.0813
## F-statistic: 4.761 on 2 and 83 DF,  p-value: 0.01103
```

```
t_to_d(0.74, 83)
```

```
## d    |        95% CI
## --------------------
## 0.16 | [-0.27, 0.59]
```

```
t_to_d(2.63, 83)
```

```
## d    |       95% CI
## -------------------
## 0.58 | [0.14, 1.01]
```

```
summary(StandSEQ2)
```

```
##
## Call:
## lm(formula = Daily_Live_Skills_standard ~ Standscale.resid +
##     SEQ_totalpt, data = MotorDLS_wSEQ2)
##
## Residuals:
##     Min      1Q  Median      3Q     Max
## -28.752 -10.279   1.584  10.251  38.153
##
## Coefficients:
##                   Estimate Std. Error t value Pr(>|t|)
## (Intercept)      109.05773    7.77982  14.018   <2e-16 ***
## Standscale.resid   2.95856    1.66496   1.777   0.0792 .
## SEQ_totalpt       -0.08869    0.03500  -2.534   0.0132 *
## ---
## Signif. codes:  0 '***' 0.001 '**' 0.01 '*' 0.05 '.' 0.1 ' ' 1
##
## Residual standard error: 15.28 on 83 degrees of freedom
## Multiple R-squared:  0.1301, Adjusted R-squared:  0.1092
## F-statistic: 6.208 on 2 and 83 DF,  p-value: 0.003073
```

```
t_to_d(1.78, 83)
```

```
## d    |        95% CI
## --------------------
## 0.39 | [-0.04, 0.82]
```

```
t_to_d(2.53, 83)
```

```
## d    |       95% CI
## -------------------
## 0.56 | [0.12, 0.99]
```

```
summary(HopSEQ2)
```

```
##
## Call:
## lm(formula = Daily_Live_Skills_standard ~ Hopscale.resid + SEQ_totalpt,
##     data = MotorDLS_wSEQ2)
##
## Residuals:
##     Min      1Q  Median      3Q     Max
## -28.554  -9.670  -0.189  11.922  38.562
##
## Coefficients:
##                 Estimate Std. Error t value Pr(>|t|)
## (Intercept)    111.35082    7.93323  14.036  < 2e-16 ***
## Hopscale.resid   0.02643    1.58170   0.017  0.98671
## SEQ_totalpt     -0.10331    0.03558  -2.904  0.00472 **
## ---
## Signif. codes:  0 '***' 0.001 '**' 0.01 '*' 0.05 '.' 0.1 ' ' 1
##
## Residual standard error: 15.57 on 83 degrees of freedom
## Multiple R-squared:  0.09703,    Adjusted R-squared:  0.07528
## F-statistic:  4.46 on 2 and 83 DF,  p-value: 0.01447
```

```
t_to_d(0.02, 83)
```

```
## d        |        95% CI
## ------------------------
## 4.39e-03 | [-0.43, 0.43]
```

```
t_to_d(2.90, 83)
```

```
## d    |       95% CI
## -------------------
## 0.64 | [0.19, 1.08]
```

```
summary(CatchSEQ2)
```

```
##
## Call:
## lm(formula = Daily_Live_Skills_standard ~ Catchscale.resid +
##     SEQ_totalpt, data = MotorDLS_wSEQ2)
##
## Residuals:
##     Min      1Q  Median      3Q     Max
## -29.407  -9.667  -0.205  11.970  36.432
##
## Coefficients:
##                   Estimate Std. Error t value Pr(>|t|)
## (Intercept)      111.07535    7.77087  14.294  < 2e-16 ***
## Catchscale.resid   1.80490    1.76104   1.025  0.30839
## SEQ_totalpt       -0.10083    0.03452  -2.921  0.00449 **
## ---
## Signif. codes:  0 '***' 0.001 '**' 0.01 '*' 0.05 '.' 0.1 ' ' 1
##
## Residual standard error: 15.47 on 83 degrees of freedom
## Multiple R-squared:  0.1083, Adjusted R-squared:  0.08683
## F-statistic: 5.041 on 2 and 83 DF,  p-value: 0.008586
```

```
t_to_d(1.03, 83)
```

```
## d    |        95% CI
## --------------------
## 0.23 | [-0.21, 0.66]
```

```
t_to_d(2.92, 83)
```

```
## d    |       95% CI
## -------------------
## 0.64 | [0.20, 1.08]
```

```
summary(DribbleSEQ2)
```

```
## 
## Call:
## lm(formula = Daily_Live_Skills_standard ~ Dribblescale.resid + 
##     SEQ_totalpt, data = MotorDLS_wSEQ2)
## 
## Residuals:
##     Min      1Q  Median      3Q     Max 
## -28.341 -10.375   1.257  11.031  38.234 
## 
## Coefficients:
##                     Estimate Std. Error t value Pr(>|t|)    
## (Intercept)        109.53140    7.75821  14.118  < 2e-16 ***
## Dribblescale.resid   3.47509    2.04585   1.699  0.09314 .  
## SEQ_totalpt         -0.09187    0.03473  -2.645  0.00976 ** 
## ---
## Signif. codes:  0 '***' 0.001 '**' 0.01 '*' 0.05 '.' 0.1 ' ' 1
## 
## Residual standard error: 15.31 on 83 degrees of freedom
## Multiple R-squared:  0.1274, Adjusted R-squared:  0.1063 
## F-statistic: 6.057 on 2 and 83 DF,  p-value: 0.003504
```

```
t_to_d(1.70, 83)
```

```
## d    |        95% CI
## --------------------
## 0.37 | [-0.06, 0.81]
```

```
t_to_d(2.65, 83)
```

```
## d    |       95% CI
## -------------------
## 0.58 | [0.14, 1.02]
```

```
summary(PushupSEQ2)
```

```
##
## Call:
## lm(formula = Daily_Live_Skills_standard ~ Pushupscale.resid +
##     SEQ_totalpt, data = MotorDLS_wSEQ2)
##
## Residuals:
##     Min      1Q  Median      3Q     Max
## -28.941  -9.689   0.397  11.397  36.871
##
## Coefficients:
##                    Estimate Std. Error t value Pr(>|t|)
## (Intercept)       112.30624    7.84660  14.313  < 2e-16 ***
## Pushupscale.resid   1.97086    2.20357   0.894  0.37370
## SEQ_totalpt        -0.10374    0.03447  -3.009  0.00347 **
## ---
## Signif. codes:  0 '***' 0.001 '**' 0.01 '*' 0.05 '.' 0.1 ' ' 1
##
## Residual standard error: 15.5 on 83 degrees of freedom
## Multiple R-squared:  0.1057, Adjusted R-squared:  0.0841
## F-statistic: 4.902 on 2 and 83 DF,  p-value: 0.009718
```

```
t_to_d(.89, 83)
```

```
## d    |        95% CI
## --------------------
## 0.20 | [-0.24, 0.63]
```

```
t_to_d(3.01, 83)
```

```
## d    |       95% CI
## -------------------
## 0.66 | [0.22, 1.10]
```

```
summary(SitupSEQ2)
```

```
##
## Call:
## lm(formula = Daily_Live_Skills_standard ~ Situpscale.resid +
##     SEQ_totalpt, data = MotorDLS_wSEQ2)
##
## Residuals:
##     Min      1Q  Median      3Q     Max
## -28.488  -9.683  -0.165  11.952  38.682
##
## Coefficients:
##                   Estimate Std. Error t value Pr(>|t|)
## (Intercept)      111.35019    7.83628  14.210  < 2e-16 ***
## Situpscale.resid  -0.07508    1.87410  -0.040  0.96814
## SEQ_totalpt       -0.10348    0.03465  -2.987  0.00371 **
## ---
## Signif. codes:  0 '***' 0.001 '**' 0.01 '*' 0.05 '.' 0.1 ' ' 1
##
## Residual standard error: 15.57 on 83 degrees of freedom
## Multiple R-squared:  0.09705,    Adjusted R-squared:  0.07529
## F-statistic:  4.46 on 2 and 83 DF,  p-value: 0.01446
```

```
t_to_d(0.04, 83)
```

```
## d        |        95% CI
## ------------------------
## 8.78e-03 | [-0.42, 0.44]
```

```
t_to_d(2.99, 83)
```

```
## d    |       95% CI
## ------------------
## 0.66 | [0.21, 1.10]
```

#Aim 2: Does IQ moderate the relation between balance and DLS?

```
MotorDLS_ASD_A2 <- subset(MotorDLS_ASD3, MotorDLS_ASD3$subject!="H022")

#These participants were part of Fisher et al. 2018.
Unique_MotorDLS_A2 <- subset(MotorDLS_ASD_A2, MotorDLS_ASD_A2$subject!="VGML_3" & MotorDLS_ASD_A2$subject!="VGML_
9" & MotorDLS_ASD_A2$subject!="VGML_10" & MotorDLS_ASD_A2$subject!="VGML_11" & MotorDLS_ASD_A2$subject!="VGML_12"
& MotorDLS_ASD_A2$subject!="VGML_13" & MotorDLS_ASD_A2$subject!="VGML_14" & MotorDLS_ASD_A2$subject!="VGML_15" &
MotorDLS_ASD_A2$subject!="VGML_16" & MotorDLS_ASD_A2$subject!="VGML_17" & MotorDLS_ASD_A2$subject!="VGML_19" & Mo
torDLS_ASD_A2$subject!="VGML_20" & MotorDLS_ASD_A2$subject!="VGML_23" &  MotorDLS_ASD_A2$subject!="VGML_24" & Mot
orDLS_ASD_A2$subject!="VGML_25" & MotorDLS_ASD_A2$subject!="VGML_26" & MotorDLS_ASD_A2$subject!="VGML_27" & Motor
DLS_ASD_A2$subject!="VGML_28" &  MotorDLS_ASD_A2$subject!="VGML_29" & MotorDLS_ASD_A2$subject!="VGML_32" & MotorD
LS_ASD_A2$subject!="H005" &  MotorDLS_ASD_A2$subject!="H006" & MotorDLS_ASD_A2$subject!="H007" &  MotorDLS_ASD_A2
$subject!="H011" & MotorDLS_ASD_A2$subject!="H014" & MotorDLS_ASD_A2$subject!="H015" &MotorDLS_ASD_A2$subject!="H
016" &  MotorDLS_ASD_A2$subject!="H018" &  MotorDLS_ASD_A2$subject!="H020" &MotorDLS_ASD_A2$subject!="H021" & Mot
orDLS_ASD_A2$subject!="H022" & MotorDLS_ASD_A2$subject!="H023"&  MotorDLS_ASD_A2$subject!="H026" & MotorDLS_ASD_A
2$subject!="H027" & MotorDLS_ASD_A2$subject!="H028" &MotorDLS_ASD_A2$subject!="H032" & MotorDLS_ASD_A2$subject!
="H041" & MotorDLS_ASD_A2$subject!="H045" & MotorDLS_ASD_A2$subject!="H051" & MotorDLS_ASD_A2$subject!="H055" & M
otorDLS_ASD_A2$subject!="N001" & MotorDLS_ASD_A2$subject!="N002" & MotorDLS_ASD_A2$subject!="N003" & MotorDLS_ASD
_A2$subject!="N004" & MotorDLS_ASD_A2$subject!="N005" & MotorDLS_ASD_A2$subject!="N006" & MotorDLS_ASD_A2$subjec
t!="N007" & MotorDLS_ASD_A2$subject!="N008" & MotorDLS_ASD_A2$subject!="N009" & MotorDLS_ASD_A2$subject!="N010" &
MotorDLS_ASD_A2$subject!="N011" & MotorDLS_ASD_A2$subject!="N012" & MotorDLS_ASD_A2$subject!="N013" & MotorDLS_AS
D_A2$subject!="N014" & MotorDLS_ASD_A2$subject!="N015" & MotorDLS_ASD_A2$subject!="N016" & MotorDLS_ASD_A2$subjec
t!="N018" & MotorDLS_ASD_A2$subject!="N022" & MotorDLS_ASD_A2$subject!="N024" & MotorDLS_ASD_A2$subject!="N026" &
MotorDLS_ASD_A2$subject!="H029")

table(Unique_MotorDLS_A2$Sex..b.1..g.0.)
```

```
##
##  0  1
##  4 31
```

```
describe(Unique_MotorDLS_A2$Age_Acc)
```

```
## Unique_MotorDLS_A2$Age_Acc
##        n  missing distinct     Info     Mean      Gmd      .05      .10
##       35        0       35        1    10.65    3.905    6.454    6.561
##      .25      .50      .75      .90      .95
##    7.710    9.469   14.279   15.389   16.001
##
## lowest : 6.22142 6.39909 6.4769  6.50821 6.64083
## highest: 15.0274 15.6299 15.9495 16.1217 16.3525
```

```
describe(Unique_MotorDLS_A2$IQ)
```

```
## Unique_MotorDLS_A2$IQ
##        n  missing distinct     Info     Mean      Gmd      .05      .10
##       35        0       25    0.999    97.54    21.09     72.7     74.2
##      .25      .50      .75      .90      .95
##     82.0    100.0    109.0    118.2    124.0
##
## lowest :  62  72  73  76  78, highest: 117 119 121 131 136
```

```
table(Unique_MotorDLS_A2$Hispanic)
```

```
##
##  0  1
## 34  1
```

```
table(Unique_MotorDLS_A2$New_ethnicity)
```

```
##
##  1  2  5  6
##  1  1 32  1
```

```
ggplot(MotorDLS_ASD_A2, aes(x=BalanceTscore, y=Daily_Live_Skills_standard))+
  geom_point()+
  stat_smooth(method="lm", formula=y~x, size=1)+ #linear
  stat_smooth(method="loess", formula=y~x, size=1, colour="red")+ #loess
  stat_smooth(method = "lm", formula = y ~ x + I(x^2), size = 1, colour="forestgreen")+ #quadratic
  stat_smooth(method = "lm", formula = y ~ log(x), colour="purple")+ #log
  stat_smooth(method = "lm", formula = y ~ exp(x), colour="orange") #exponential
```

```
## Warning: Using `size` aesthetic for lines was deprecated in ggplot2 3.4.0.
## ℹ Please use `linewidth` instead.
## This warning is displayed once every 8 hours.
## Call `lifecycle::last_lifecycle_warnings()` to see where this warning was
## generated.
```

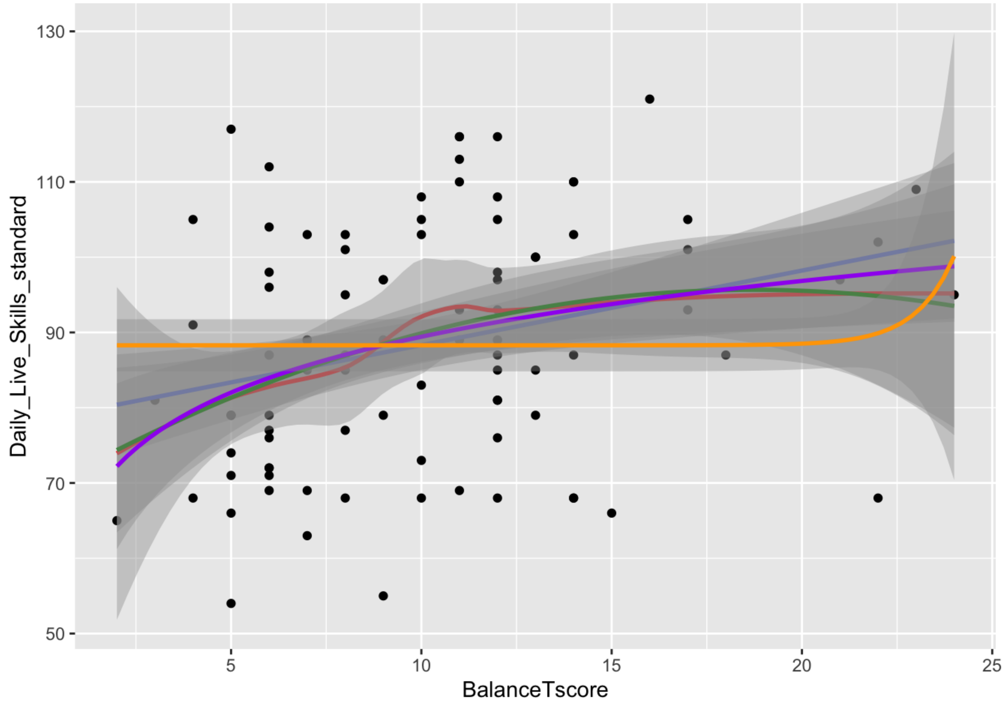


```
Balance_lin <- lm(Daily_Live_Skills_standard~BalanceTscore*IQ, data=MotorDLS_ASD_A2)
Balance_quad_bothIQ <- lm(Daily_Live_Skills_standard~BalanceTscore*IQ + I(BalanceTscore^2)*IQ, data=MotorDLS_ASD_
A2)
Balance_quad_BalanceIQ <- lm(Daily_Live_Skills_standard~BalanceTscore*IQ + I(BalanceTscore^2), data=MotorDLS_ASD_
A2)
Balance_exp <- lm(Daily_Live_Skills_standard~I(exp(BalanceTscore))*IQ, data=MotorDLS_ASD_A2)
Balance_log <- lm(Daily_Live_Skills_standard~log(BalanceTscore), data=MotorDLS_ASD_A2)

#Compare fit
anova(Balance_quad_bothIQ, Balance_quad_BalanceIQ)
```

```
## Analysis of Variance Table
##
## Model 1: Daily_Live_Skills_standard ~ BalanceTscore * IQ + I(BalanceTscore^2) *
##     IQ
## Model 2: Daily_Live_Skills_standard ~ BalanceTscore * IQ + I(BalanceTscore^2)
##   Res.Df   RSS Df Sum of Sq      F Pr(>F)
## 1     83 17984
## 2     84 18224 -1   -239.93 1.1073 0.2957
```

```
anova(Balance_lin, Balance_exp)
```

```
## Analysis of Variance Table
##
## Model 1: Daily_Live_Skills_standard ~ BalanceTscore * IQ
## Model 2: Daily_Live_Skills_standard ~ I(exp(BalanceTscore)) * IQ
##   Res.Df   RSS Df Sum of Sq F Pr(>F)
## 1     85 18245
## 2     85 19964  0   -1719.3
```

```
anova(Balance_lin, Balance_log)
```

```
## Analysis of Variance Table
##
## Model 1: Daily_Live_Skills_standard ~ BalanceTscore * IQ
## Model 2: Daily_Live_Skills_standard ~ log(BalanceTscore)
##   Res.Df   RSS Df Sum of Sq      F   Pr(>F)
## 1     85 18245
## 2     87 20699 -2   -2454.4 5.7173 0.004682 **
## ---
## Signif. codes:  0 '***' 0.001 '**' 0.01 '*' 0.05 '.' 0.1 ' ' 1
```

```
anova(Balance_log, Balance_exp)
```

```
## Analysis of Variance Table
##
## Model 1: Daily_Live_Skills_standard ~ log(BalanceTscore)
## Model 2: Daily_Live_Skills_standard ~ I(exp(BalanceTscore)) * IQ
##   Res.Df   RSS Df Sum of Sq      F Pr(>F)
## 1     87 20699
## 2     85 19964  2    735.09 1.5649 0.2151
```

```
anova(Balance_lin, Balance_quad_BalanceIQ)
```

```
## Analysis of Variance Table
##
## Model 1: Daily_Live_Skills_standard ~ BalanceTscore * IQ
## Model 2: Daily_Live_Skills_standard ~ BalanceTscore * IQ + I(BalanceTscore^2)
##   Res.Df   RSS Df Sum of Sq      F Pr(>F)
## 1     85 18245
## 2     84 18224  1    20.227 0.0932 0.7609
```

```
summary(Balance_lin)
```

```
##
## Call:
## lm(formula = Daily_Live_Skills_standard ~ BalanceTscore * IQ,
##     data = MotorDLS_ASD_A2)
##
## Residuals:
##     Min      1Q  Median      3Q     Max
## -31.948  -9.764  -0.390  10.249  26.470
##
## Coefficients:
##                   Estimate Std. Error t value Pr(>|t|)
## (Intercept)        7.49779   23.16530   0.324  0.74699
## BalanceTscore      5.51262    2.32059   2.376  0.01977 *
## IQ                 0.71659    0.22436   3.194  0.00197 **
## BalanceTscore:IQ -0.04546    0.02159  -2.106  0.03818 *
## ---
## Signif. codes:  0 '***' 0.001 '**' 0.01 '*' 0.05 '.' 0.1 ' ' 1
##
## Residual standard error: 14.65 on 85 degrees of freedom
## Multiple R-squared:  0.2057, Adjusted R-squared:  0.1777
## F-statistic: 7.339 on 3 and 85 DF,  p-value: 0.0001968
```

```
t_to_d(-2.11, 85)
```

```
## d     |         95% CI
## ----------------------
## -0.46 | [-0.89, -0.03]
```

```
ggplot(Unique_MotorDLS_A2, aes(x=BalanceTscore, y=Daily_Live_Skills_standard))+
  geom_point()+
  stat_smooth(method="lm", formula=y~x, size=1)+
  stat_smooth(method="loess", formula=y~x, size=1, colour="red")+
  stat_smooth(method = "lm", formula = y ~ x + I(x^2), size = 1, colour="forestgreen")+
  stat_smooth(method = "lm", formula = y ~ log(x), colour="purple")+
  stat_smooth(method = "lm", formula = y ~ exp(x), colour="orange")
```

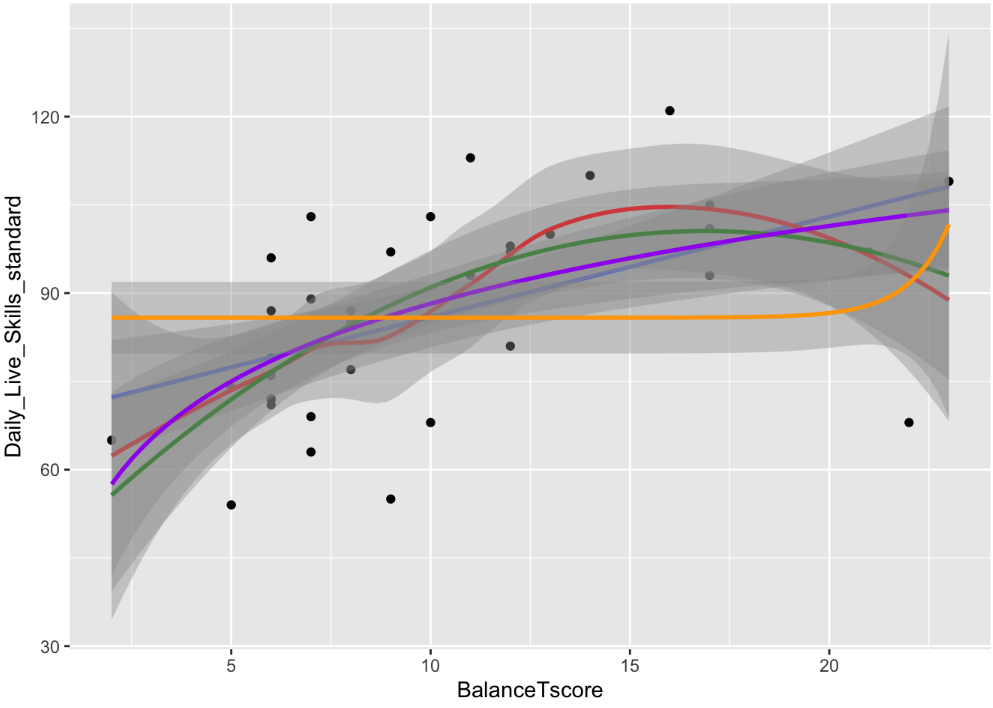

```
Balance_lin_mini <- lm(Daily_Live_Skills_standard~BalanceTscore*IQ, data=Unique_MotorDLS_A2)
Balance_quad_mini <- lm(Daily_Live_Skills_standard~BalanceTscore*IQ + I(BalanceTscore^2), data=Unique_MotorDLS_A
2)
Balance_quad_mini_bothIQ <- lm(Daily_Live_Skills_standard~BalanceTscore*IQ + I(BalanceTscore^2)*IQ, data=Unique_M
otorDLS_A2)

anova(Balance_quad_mini, Balance_quad_mini_bothIQ)
```

```
## Analysis of Variance Table
##
## Model 1: Daily_Live_Skills_standard ~ BalanceTscore * IQ + I(BalanceTscore^2)
## Model 2: Daily_Live_Skills_standard ~ BalanceTscore * IQ + I(BalanceTscore^2) *
##     IQ
##   Res.Df    RSS Df Sum of Sq      F Pr(>F)
## 1     30 5821.1
## 2     29 5816.7  1    4.3426 0.0217  0.884
```

```
anova(Balance_lin_mini, Balance_quad_mini)
```

```
## Analysis of Variance Table
##
## Model 1: Daily_Live_Skills_standard ~ BalanceTscore * IQ
## Model 2: Daily_Live_Skills_standard ~ BalanceTscore * IQ + I(BalanceTscore^2)
##   Res.Df    RSS Df Sum of Sq      F  Pr(>F)
## 1     31 6655.0
## 2     30 5821.1  1    833.91 4.2977 0.04685 *
## ---
## Signif. codes:  0 '***' 0.001 '**' 0.01 '*' 0.05 '.' 0.1 ' ' 1
```

```
summary(Balance_lin_mini)
```

```
##
## Call:
## lm(formula = Daily_Live_Skills_standard ~ BalanceTscore * IQ,
##     data = Unique_MotorDLS_A2)
##
## Residuals:
##     Min      1Q  Median      3Q     Max
## -35.288  -7.578   1.776   7.016  23.391
##
## Coefficients:
##                  Estimate Std. Error t value Pr(>|t|)
## (Intercept)      14.41896   31.74688   0.454   0.6529
## BalanceTscore     5.71091    2.93847   1.943   0.0611 .
## IQ                0.56277    0.31919   1.763   0.0877 .
## BalanceTscore:IQ -0.03987    0.02716  -1.468   0.1523
## ---
## Signif. codes:  0 '***' 0.001 '**' 0.01 '*' 0.05 '.' 0.1 ' ' 1
##
## Residual standard error: 14.65 on 31 degrees of freedom
## Multiple R-squared:  0.3368, Adjusted R-squared:  0.2726
## F-statistic: 5.248 on 3 and 31 DF,  p-value: 0.004797
```

```
t_to_d(1.94, 31)
```

```
## d    |        95% CI
## --------------------
## 0.70 | [-0.03, 1.42]
```

```
t_to_d(1.76, 31)
```

```
## d    |        95% CI
## --------------------
## 0.63 | [-0.09, 1.35]
```

```
t_to_d(-1.47, 31)
```

```
## d     |        95% CI
## ---------------------
## -0.53 | [-1.24, 0.19]
```

```
median(MotorDLS_ASD_A2$IQ)
```

```
## [1] 104
```

```
MotorDLS_ASD_A2$IQgroup <- ifelse(MotorDLS_ASD_A2$IQ>median(MotorDLS_ASD_A2$IQ),"Above Median IQ (>=104)", "Below
Median IQ (<104)")

HighIQ <- subset(MotorDLS_ASD_A2, MotorDLS_ASD_A2$IQ>median(MotorDLS_ASD_A2$IQ))
LowIQ <- subset(MotorDLS_ASD_A2, MotorDLS_ASD_A2$IQ<median(MotorDLS_ASD_A2$IQ))

cor.test(HighIQ$BalanceTscore, HighIQ$Daily_Live_Skills_standard)
```

```
##
##  Pearson's product-moment correlation
##
## data:  HighIQ$BalanceTscore and HighIQ$Daily_Live_Skills_standard
## t = 0.10861, df = 42, p-value = 0.914
## alternative hypothesis: true correlation is not equal to 0
## 95 percent confidence interval:
##  -0.2815243  0.3120844
## sample estimates:
##        cor
## 0.01675658
```

```
cor.test(LowIQ$BalanceTscore, LowIQ$Daily_Live_Skills_standard)
```

```
##
##  Pearson's product-moment correlation
##
## data:  LowIQ$BalanceTscore and LowIQ$Daily_Live_Skills_standard
## t = 2.6814, df = 40, p-value = 0.0106
## alternative hypothesis: true correlation is not equal to 0
## 95 percent confidence interval:
##  0.09804041 0.62064110
## sample estimates:
##       cor
## 0.3903406
```

```
ggplot(MotorDLS_ASD_A2, aes(x=BalanceTscore, y=Daily_Live_Skills_standard, colour=IQgroup, shape=IQgroup, linetyp
e=IQgroup))+
  geom_point()+
  geom_smooth(method=lm, alpha=.2)+
  scale_colour_manual(values=c("#0072B2", "#009E73"))+
  scale_shape_manual(values=c(1,2))+
  annotate(geom="text", x=20, y=62, label="r = .01, p = .91", colour="#0072B2")+
  annotate(geom="text", x=20, y=58, label="r = .39, p = .01", colour="#009E73")+
  labs(x="BOT-2 Balance T-score", y="Vineland DLS Standard Scores")+
  theme_bw()+
  theme(legend.title=element_blank())+
  theme(legend.position="bottom")+
  theme(axis.title = element_text(face="bold", size=12))
```

```
## `geom_smooth()` using formula = 'y ~ x'
```

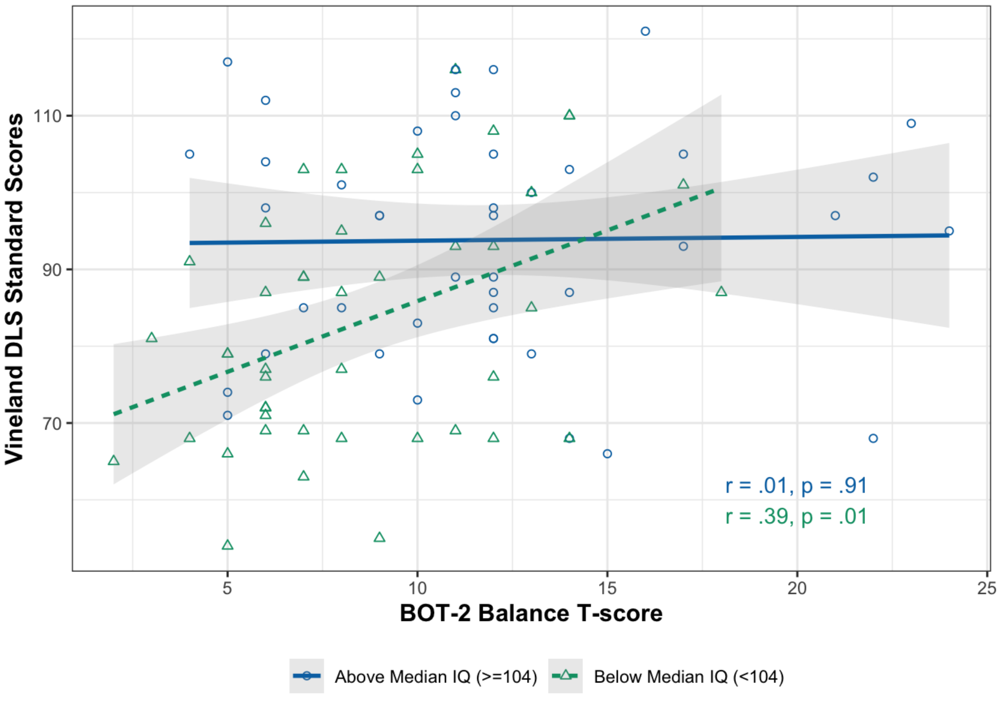


```
ggsave("SERP_fig2.png", height=6, width=6)
```

```
## `geom_smooth()` using formula = 'y ~ x'
```

#Supplementary Materials

```
median(MotorDLS_ASD3$IQ)
```

```
## [1] 104.5
```

```
HighIQ_full <- subset(MotorDLS_ASD3, MotorDLS_ASD3$IQ>median(MotorDLS_ASD3$IQ))
LowIQ_full <- subset(MotorDLS_ASD3, MotorDLS_ASD3$IQ<median(MotorDLS_ASD3$IQ))

table(HighIQ_full$Sex..b.1..g.0.)
```

```
##
##  0  1
##  5 40
```

```
table(HighIQ_full$Hispanic)
```

```
##
##  0  1
## 42  3
```

```
table(HighIQ_full$New_ethnicity)
```

```
##
##  2  4  5  6
##  1  2 39  3
```

```
describe(HighIQ_full$Age_Acc)
```

```
## HighIQ_full$Age_Acc
##        n  missing distinct     Info     Mean      Gmd      .05      .10
##       45        0       45        1    11.71    3.863    7.166    7.668
##      .25      .50      .75      .90      .95
##    9.469   10.544   14.877   16.614   17.093
##
## lowest : 6.39357 6.50821 7.15726 7.20226 7.58333
## highest: 16.7883 16.9127 17.1381 17.2379 17.847
```

```
describe(HighIQ_full$IQ)
```

```
## HighIQ_full$IQ
##        n  missing distinct     Info     Mean      Gmd      .05      .10
##       45        0       19    0.995    115.5    9.727    105.0    106.0
##      .25      .50      .75      .90      .95
##    109.0    114.0    121.0    128.6    134.2
##
## Value        105   106   107   108   109   110   111   112   114   115   116
## Frequency      4     2     3     1     5     3     1     3     1     2     1
## Proportion 0.089 0.044 0.067 0.022 0.111 0.067 0.022 0.067 0.022 0.044 0.022
##
## Value        117   119   121   123   125   131   135   136
## Frequency      2     5     4     1     2     2     2     1
## Proportion 0.044 0.111 0.089 0.022 0.044 0.044 0.044 0.022
##
## For the frequency table, variable is rounded to the nearest 0
```

```
describe(HighIQ_full$SRSTscore)
```

```
## HighIQ_full$SRSTscore
##        n  missing distinct     Info     Mean      Gmd      .05      .10
##       45        0       21    0.995    73.78    10.14     62.0     64.8
##      .25      .50      .75      .90      .95
##     67.0     73.0     80.0     85.6     89.8
##
## lowest : 48 57 62 64 66, highest: 82 85 86 89 90
```

```
describe(HighIQ_full$SCQ_Total_Raw_Score)
```

```
## HighIQ_full$SCQ_Total_Raw_Score
##        n  missing distinct     Info     Mean      Gmd      .05      .10
##       45        0       21    0.997    17.53     7.78      7.0      8.0
##      .25      .50      .75      .90      .95
##     11.0     18.0     23.0     26.6     27.8
##
## lowest :  6  7  8  9 10, highest: 24 26 27 28 30
```

```
describe(HighIQ_full$BOT2_shorttotal_mot_sscore)
```

```
## HighIQ_full$BOT2_shorttotal_mot_sscore
##        n  missing distinct     Info     Mean      Gmd      .05      .10
##       45        0       19    0.995    41.09     7.97     32.2     33.4
##      .25      .50      .75      .90      .95
##     36.0     39.0     43.0     51.2     53.0
##
## Value         32    33    34    35    36    37    38    39    40    41    42
## Frequency      3     2     2     3     3     1     5     4     5     1     3
## Proportion 0.067 0.044 0.044 0.067 0.067 0.022 0.111 0.089 0.111 0.022 0.067
##
## Value         43    44    46    50    52    53    61    68
## Frequency      3     1     1     3     1     2     1     1
## Proportion 0.067 0.022 0.022 0.067 0.022 0.044 0.022 0.022
##
## For the frequency table, variable is rounded to the nearest 0
```

```
describe(HighIQ_full$BalanceTscore)
```

```
## HighIQ_full$BalanceTscore
##        n  missing distinct     Info     Mean      Gmd      .05      .10
##       44        1       18    0.989    11.82    5.564     5.00     6.00
##      .25      .50      .75      .90      .95
##     8.75    12.00    14.00    19.80    22.00
##
## Value          4     5     6     7     8     9    10    11    12    13    14
## Frequency      1     3     4     1     2     3     3     4     9     2     3
## Proportion 0.023 0.068 0.091 0.023 0.045 0.068 0.068 0.091 0.205 0.045 0.068
##
## Value         15    16    17    21    22    23    24
## Frequency      1     1     2     1     2     1     1
## Proportion 0.023 0.023 0.045 0.023 0.045 0.023 0.023
##
## For the frequency table, variable is rounded to the nearest 0
```

```
describe(HighIQ_full$Daily_Live_Skills_standard)
```

```
## HighIQ_full$Daily_Live_Skills_standard
##        n  missing distinct     Info     Mean      Gmd      .05      .10
##       45        0       29    0.998    93.96    16.95     68.6     73.4
##      .25      .50      .75      .90      .95
##     83.0     97.0    105.0    112.6    116.0
##
## lowest :  66  68  71  73  74, highest: 112 113 116 117 121
```

```
describe(HighIQ_full$ADOS2_M3_Total)
```

```
## HighIQ_full$ADOS2_M3_Total
##        n  missing distinct     Info     Mean      Gmd      .05      .10
##       26       19       15    0.994    10.65    5.388     4.00     4.50
##      .25      .50      .75      .90      .95
##     8.00    10.00    14.00    16.00    16.75
##
## Value          3     4     5     6     8     9    10    11    12    13    14
## Frequency      1     2     2     1     2     3     3     1     2     1     2
## Proportion 0.038 0.077 0.077 0.038 0.077 0.115 0.115 0.038 0.077 0.038 0.077
##
## Value         15    16    17    21
## Frequency      1     3     1     1
## Proportion 0.038 0.115 0.038 0.038
##
## For the frequency table, variable is rounded to the nearest 0
```

```
describe(HighIQ_full$ADOS2_M4_Total)
```

```
## HighIQ_full$ADOS2_M4_Total
##        n  missing distinct     Info     Mean      Gmd      .05      .10
##       19       26       10    0.987    12.53    5.181      6.4      7.8
##      .25      .50      .75      .90      .95
##     10.0     12.0     16.0     17.6     20.0
##
## Value          1     7     8    10    11    12    13    16    17    20
## Frequency      1     1     1     3     1     3     3     2     2     2
## Proportion 0.053 0.053 0.053 0.158 0.053 0.158 0.158 0.105 0.105 0.105
##
## For the frequency table, variable is rounded to the nearest 0
```

```
table(LowIQ_full$Sex..b.1..g.0.)
```

```
##
##  0  1
##  6 39
```

```
table(LowIQ_full$Hispanic)
```

```
##
##  0  1
## 42  3
```

```
table(LowIQ_full$New_ethnicity)
```

```
##
##  1  2  4  5  6
##  1  4  4 34  2
```

```
describe(LowIQ_full$Age_Acc)
```

```
## LowIQ_full$Age_Acc
##        n  missing distinct     Info     Mean      Gmd      .05      .10
##       45        0       45        1    11.46    4.148    6.426    6.852
##      .25      .50      .75      .90      .95
##    8.608   10.388   14.730   16.123   16.276
##
## lowest : 6.22142 6.39909 6.41273 6.4769  6.64083
## highest: 16.1244 16.194  16.2965 17.3267 17.5082
```

```
describe(LowIQ_full$IQ)
```

```
## LowIQ_full$IQ
##        n  missing distinct     Info     Mean      Gmd      .05      .10
##       45        0       22    0.995    89.09     13.1     72.2     73.0
##      .25      .50      .75      .90      .95
##     79.0     94.0     99.0    101.2    103.6
##
## lowest :  62  67  72  73  75, highest:  98  99 100 102 104
```

```
describe(LowIQ_full$SRSTscore)
```

```
## LowIQ_full$SRSTscore
##        n  missing distinct     Info     Mean      Gmd      .05      .10
##       45        0       26    0.995    76.56    12.84     53.2     57.0
##      .25      .50      .75      .90      .95
##     71.0     79.0     85.0     90.0     90.0
##
## lowest : 48 51 53 54 55, highest: 84 85 87 88 90
```

```
describe(LowIQ_full$SCQ_Total_Raw_Score)
```

```
## LowIQ_full$SCQ_Total_Raw_Score
##        n  missing distinct     Info     Mean      Gmd      .05      .10
##       44        1       21    0.997    20.41    8.786     8.15    11.00
##      .25      .50      .75      .90      .95
##    15.00    20.00    26.25    30.00    33.00
##
## lowest :  8  9 11 12 13, highest: 28 29 30 33 37
```

```
describe(LowIQ_full$BOT2_shorttotal_mot_sscore)
```

```
## LowIQ_full$BOT2_shorttotal_mot_sscore
##        n  missing distinct     Info     Mean      Gmd      .05      .10
##       44        1       18    0.995    35.75    5.673       28       30
##      .25      .50      .75      .90      .95
##       32       36       40       41       42
##
## Value         24    27    28    30    31    32    33    34    35    36    37
## Frequency      1     1     2     3     3     4     1     1     5     2     2
## Proportion 0.023 0.023 0.045 0.068 0.068 0.091 0.023 0.023 0.114 0.045 0.045
##
## Value         38    39    40    41    42    44    45
## Frequency      3     4     4     4     2     1     1
## Proportion 0.068 0.091 0.091 0.091 0.045 0.023 0.023
##
## For the frequency table, variable is rounded to the nearest 0
```

```
describe(LowIQ_full$BalanceTscore)
```

```
## LowIQ_full$BalanceTscore
##        n  missing distinct     Info     Mean      Gmd      .05      .10
##       45        0       15     0.99    8.622    4.145      4.0      5.0
##      .25      .50      .75      .90      .95
##      6.0      8.0     11.0     13.6     14.0
##
## Value          2     3     4     5     6     7     8     9    10    11    12
## Frequency      1     1     2     4     8     5     5     2     3     3     4
## Proportion 0.022 0.022 0.044 0.089 0.178 0.111 0.111 0.044 0.067 0.067 0.089
##
## Value         13    14    17    18
## Frequency      2     3     1     1
## Proportion 0.044 0.067 0.022 0.022
##
## For the frequency table, variable is rounded to the nearest 0
```

```
describe(LowIQ_full$Daily_Live_Skills_standard)
```

```
## LowIQ_full$Daily_Live_Skills_standard
##        n  missing distinct     Info     Mean      Gmd      .05      .10
##       45        0       27    0.997    83.33    18.36     63.4     66.8
##      .25      .50      .75      .90      .95
##     69.0     81.0     95.0    104.2    109.6
##
## lowest :  54  55  63  65  66, highest: 103 105 108 110 116
```

```
describe(LowIQ_full$ADOS2_M3_Total)
```

```
## LowIQ_full$ADOS2_M3_Total
##        n  missing distinct     Info     Mean      Gmd      .05      .10
##       29       16       14    0.987    14.41    5.635      7.4      8.0
##      .25      .50      .75      .90      .95
##     11.0     14.0     16.0     21.2     23.8
##
## Value          7     8    11    12    13    14    15    16    18    19    21
## Frequency      2     2     6     2     2     2     2     4     2     1     1
## Proportion 0.069 0.069 0.207 0.069 0.069 0.069 0.069 0.138 0.069 0.034 0.034
##
## Value         22    25    27
## Frequency      1     1     1
## Proportion 0.034 0.034 0.034
##
## For the frequency table, variable is rounded to the nearest 0
```

```
describe(LowIQ_full$ADOS2_M4_Total)
```

```
## LowIQ_full$ADOS2_M4_Total
##        n  missing distinct     Info     Mean      Gmd      .05      .10
##       16       29       12    0.991     14.5    7.233     7.00     9.00
##      .25      .50      .75      .90      .95
##    10.00    15.00    18.25    21.00    23.50
##
## Value          1     9    10    11    13    15    17    18    19    20    22
## Frequency      1     2     2     1     1     3     1     1     1     1     1
## Proportion 0.062 0.125 0.125 0.062 0.062 0.188 0.062 0.062 0.062 0.062 0.062
##
## Value         28
## Frequency      1
## Proportion 0.062
##
## For the frequency table, variable is rounded to the nearest 0
```

```
ID <- subset(MotorDLS_ASD3, MotorDLS_ASD3$IQ<70) #2
ID80 <- subset(MotorDLS_ASD3, MotorDLS_ASD3$IQ<80) #12
```
